# Stress and salivary cortisol in emergency medical dispatchers: A randomized shifts control trial

**DOI:** 10.1371/journal.pone.0177094

**Published:** 2017-05-15

**Authors:** Sarah Bedini, François Braun, Laurence Weibel, Michel Aussedat, Bruno Pereira, Frédéric Dutheil

**Affiliations:** 1 The Mercy Regional Hospital Centre (CHR) of Metz-Thionville, Emergency department, Ars-Laquenexy, France; 2 SAMU-Urgences de France, Paris, France; 3 The CARSAT Alsace, Strasbourg, France; 4 University Hospital of Clermont-Ferrand, CHU Clermont-Ferrand, Clinical Research Direction, Clermont-Ferrand, France; 5 Université Clermont Auvergne, CNRS, LaPSCo, Physiological and Psychosocial Stress, University Hospital of Clermont-Ferrand, CHU Clermont-Ferrand, Preventive and Occupational Medicine, WittyFit, Clermont-Ferrand, France; 6 Australian Catholic University, Faculty of Health, School of Exercise Science, Melbourne, Victoria, Australia; Weill Cornell Medical College Qatar, QATAR

## Abstract

Stress at work is a public health concern. Phone operators in emergency medical dispatch centers are particularly at risk. We aimed to demonstrate that the most stressful time for emergency medical dispatchers is the shift when they receive emergency incoming calls, with cortisol as a biomarker of stress. For each emergency medical dispatcher, we measured outcomes over a control day and during three types of shift: *Incoming emergency call*, *Dispatch* and *Re-assessment*. The pattern of shifts was randomized. Saliva was sampled every 15 minutes for 2 hours, i.e. 6 consecutive times, starting 15 minutes after the first life-and-death incoming emergency call between 2 and 5 pm during three types of shift. We measured saliva cortisol every 2 hours over a control day, from 7am to 9pm. Perceived stress was assessed by a visual analog scale. We recruited 22 phone operators aged 36.4+/-10.8 years old (14 women and 8 men). Cortisol values were higher during the *Incoming emergency call* shift than during the *Dispatch* (p = .04) and *Re-assessment* (p = .04) shifts. The increase in cortisol levels was greater in men than in women (p = .009). There were no differences between control values and those of the three shifts. The kinetics of cortisol increased with greater perceived stress overall (p < .001) and for each type of shift (*Incoming emergency call*, p = .02; *Dispatch* p = .03; *Re-assessment*: p < .001). The kinetics of cortisol in response to incoming emergency calls was greater when the call was an absolute emergency (p = .03), and also tended to further increase when a subsequent absolute incoming emergency call was received (p = 0.07). In conclusion, the incoming emergency call shift carries particular risk for dispatchers, who have greater perceived stress and a greater increase in cortisol levels.

## Introduction

Stress at work is a public health concern [1,2,3,4]. Emergency medicine is a field where stress is constant [5,6,7]. An emergency, by definition, requires a rapid response with no possibility of predicting the workload [8,9]. Phone operators in emergency medical dispatch centers are particularly at risk because they must assess the gravity of the situation without seeing patients, a judgement that requires experience [10]. They must localize the victims as quickly as possible and deal with aggressive and violent behavior from patients and their relatives, excessive alcohol consumption and road accidents sometimes involving young children [11]. Stress in emergency medical dispatchers can lead to work-related exhaustion with various physical and psychological symptoms [12], and delayed decision-making due to misjudgment of the seriousness of the patient’s condition [13]. All these factors contribute to increased sick leave [14] and an increased turnover of phone operators owing to their premature departure to other less stressful professions [15]. Objective measurements of stress are therefore needed to develop evaluable preventive strategies of stress management [16,17,18]. To identify stress at a biological level, cortisol is the historical biomarker which can now be assessed easily and pain-free from saliva [19,20,21,22,23]. To our knowledge, only one study has used objective measurements to assess stress in emergency medical dispatchers [6]. The authors observed higher levels in the phone operators than in controls but did not compare levels at different shift times. In addition, although it is known that cortisol levels are related to perceived stress and acute mental stressful events [19,20,21,22,24], they did not investigate the specific effect of incoming emergency calls. Finally, the increase in cortisol levels in response to stress is greater in females [25] and in non-experienced workers/individuals [19,20,21,22].

From these observations, we hypothesized that 1) cortisol levels are a relevant biomarker of stress in emergency medical dispatchers during the *Incoming emergency call* shift 2) cortisol levels are related to perceived stress and 3) are able to identify stressful events (severity of incoming emergency call or effects of a subsequent absolute incoming emergency call) and 4) are related to sociodemographic variables such as gender and experience.

Thus, the aims of our study were to obtain objective assessments of stress by measuring saliva cortisol levels during the various shifts worked by emergency medical dispatchers, and to investigate the independent associations between perceived stress, severity of incoming calls and other variables.

## Methods

### Participants

Participants were phone operators working in the emergency dispatch center of the Mercy Regional Hospital Centre (CHR) of Metz-Thionville, France. Participants were recruited in May 2014 and we obtained complete data by October 2014. We recruited both men and women to better represent the whole team of medical dispatchers. Exclusion criteria were psychiatric disorders including depressive symptoms > 8 on the Hospital and Anxiety Scale [26], recent extraprofessional life stress event (such as death of a near relative, divorce) [27], alcohol consumption > 3 drinks per day for men and > 2 for women [28], endocrine disorder [29], oral contraceptive [25,30] and pregnancy [31], any medications used to modulate inflammatory diseases taken over the previous three months (corticosteroids, anti-inflammatory drugs, immunomodulatory drugs) [32], intensive sports > 3 hours per week of intense physical activity [33], and any current illness/infection [34] at the time of measurements (feeling feverish sensations or having hyperthermia above 38.5°C) [35]. The protocol was approved by the Human Ethics Committee of the University Hospital of Nancy, France (Clinical Trials identifier NCT02075424). The study was explained to the phone operators, who provided their written informed consent. We did not exclude non-menopausal women because basal cortisol levels do not differ during the menstrual cycle [31]. In addition, studies involving work stress and cortisol levels in acute care settings have mainly involved women [36,37,38,39].

### Work description of an emergency medical dispatcher

The same phone operator alternated three types of shifts.

**- Incoming emergency calls**: Dealing with emergency calls is considered to be the most stressful situation. The phone operator is the first to answer incoming calls and must evaluate the severity of the situation and choose from among the following courses of action: absolute emergency situation immediately requiring a mobile intensive care unit (MICU); relative emergency situation that needs to be first managed by phone by an emergency physician to decide the best option; relative emergency situation that can be managed on site by a primary care physician; no emergency situation and transfer the call to a general practitioner primary care physician for advice.

**- Dispatch**: The phone operator manages the deployment of the different intervention teams. She or he must organize transport by calling the MICU, private ambulances or the fire department depending on the severity of the case. She or he always follows the orders of the emergency physician or the general practitioner primary care physician of the emergency medical dispatch center.

**- Re-assessment**: The phone operator must re-assess the different interventions and list the management of each patient.

All phone operators do only one kind of shift per working day. All included EMDs had the same working schedule i.e. 7am-7pm. As the impact of night shifts on biomarkers of stress may last several days [40,41], we included in this study only shifts which did not follow a night shift (7pm-7am) over the three previous days.

### Shift randomization

For each phone operator, we measured outcomes during a control day and during the three types of shifts: *Incoming emergency call*, *Dispatch* and *Re-assessment*. Latin squares were used to randomize first the order between *Incoming emergency call* and *control day*. The *Incoming emergency call* shift was always before the two other types of shifts. However, after measurements were made during the *Incoming emergency call* shift, Latin squares were used to randomize the pattern of shifts between *Dispatch* and *Re-assessment*. All outcome data remained blinded until the end of the study.

### Main outcome: Saliva cortisol

During the *Incoming emergency call* shift, saliva was sampled every 15 minutes for 2 hours, i.e. 7 consecutive times (+15min, +30 min, +45min, +60 min, +75min, +90 min, +120 min), starting 15 minutes after the first life-and-death incoming emergency call between 2 and 5 pm. Sampling was then performed 7 consecutive times during the other two shifts. We began sampling at the same time for each shift. As fasting affects cortisol levels [42], we controlled that participants had lunch during shifts. On the other hand, to avoid contamination of salivary samples, participants were asked to fast and refrain from brushing teeth and chewing gum within the hour before the putative beginning of sampling (4pm).

As chronic stress may affect the nictemeral cycle of cortisol [26,27], we measured saliva cortisol levels every 2 hours during a control day, from 7am to 9pm [29]. The control day was a day-off after at least 8 days of vacation, without jet lag.

### Secondary outcomes

**Socio-demographic variables** (age, gender, and experience in the unit) were retrieved by self-reported questionnaires.

**Perceived stress** was assessed by a visual analog scale (VAS), which is a horizontal, non-calibrated line of 100 mm, ranging from very low (0) to very high (100) [43]. Perceived stress was measured at the same time as the first saliva sampling during the three types of shifts.

During the *Incoming emergency call* shift, the severity of the calls was expressed as absolute or relative. Any subsequent absolute incoming emergency call was also recorded.

Phone operators had to report any stressful events during the control day.

### Biochemical measurements

Assays were done on saliva samples, since saliva is a good alternative to steroid hormones, in particular stress hormones, including those of the HPA axis [44]. Cortisol levels were assayed by commercial ELISA kits (Salimetrics, State College, USA), according to the manufacturer’s recommendations (including sampling step). All samples were analyzed in duplicate. Mean values of duplicates were used to represent measured values. Sensitivity and intra- and inter-assay coefficients of variation were 0.05 ng/ml, 7% and 9.3%, respectively. The saliva kit was designed to avoid cortisone cross-reactivity [45].

### Statistics

The sample size calculation was estimated on the basis of a previous study by members of our team [6] which showed a 22.8% increase in cortisol levels in emergency medical dispatchers compared with control participants. Following the recommendations of Cohen [46], who has defined effect-size bounds as small (ES: 0.2), medium (ES: 0.5) and large (ES: 0.8, “‘grossly perceptible and therefore large”), we decided to include 22 participants to evidence an effect-size equals 0.7 at +120min for a two-sided type I-error at 0.017 (correction due to multiple comparisons), a statistical power of 80%, and within subject variability at 0.5 (due to the experimental design).

Statistical analyses were performed with Stata software (version 13, StataCorp, College Station, TX, US). Data were presented as mean ± standard deviation (SD). A Shapiro-Wilk test was used to test the assumption of distribution normality of cortisol. For paired comparisons (according to time of first life-and-death incoming emergency call, type of shift and control day values), standard statistical tests were performed, either paired Student t-test or Wilcoxon test when appropriate. To compare the longitudinal kinetics of this parameter in the three shifts, a zero-inflated Poisson random-effects model [47,48,49,50] was used to take into account (1) between- and within-subject variability and (2) statistical distribution characterized by frequent zero-valued observations (random event containing excess zero-count data in unit time). The zero-inflated Poisson model employs two components that correspond to two zero generating processes. The first process is governed by a binary distribution that generates structural zeros. The second process is governed by a Poisson distribution that generates counts some of which may be zero. The covariates were studied according to their clinical relevance, as fixed effects with a particular attention on interaction effects. All tests were two-sided, with a type I error set at α = 0.05, taking into account when appropriate correction of type I error due to multiple comparisons.

## Results

### Participants

**Fig 1:**
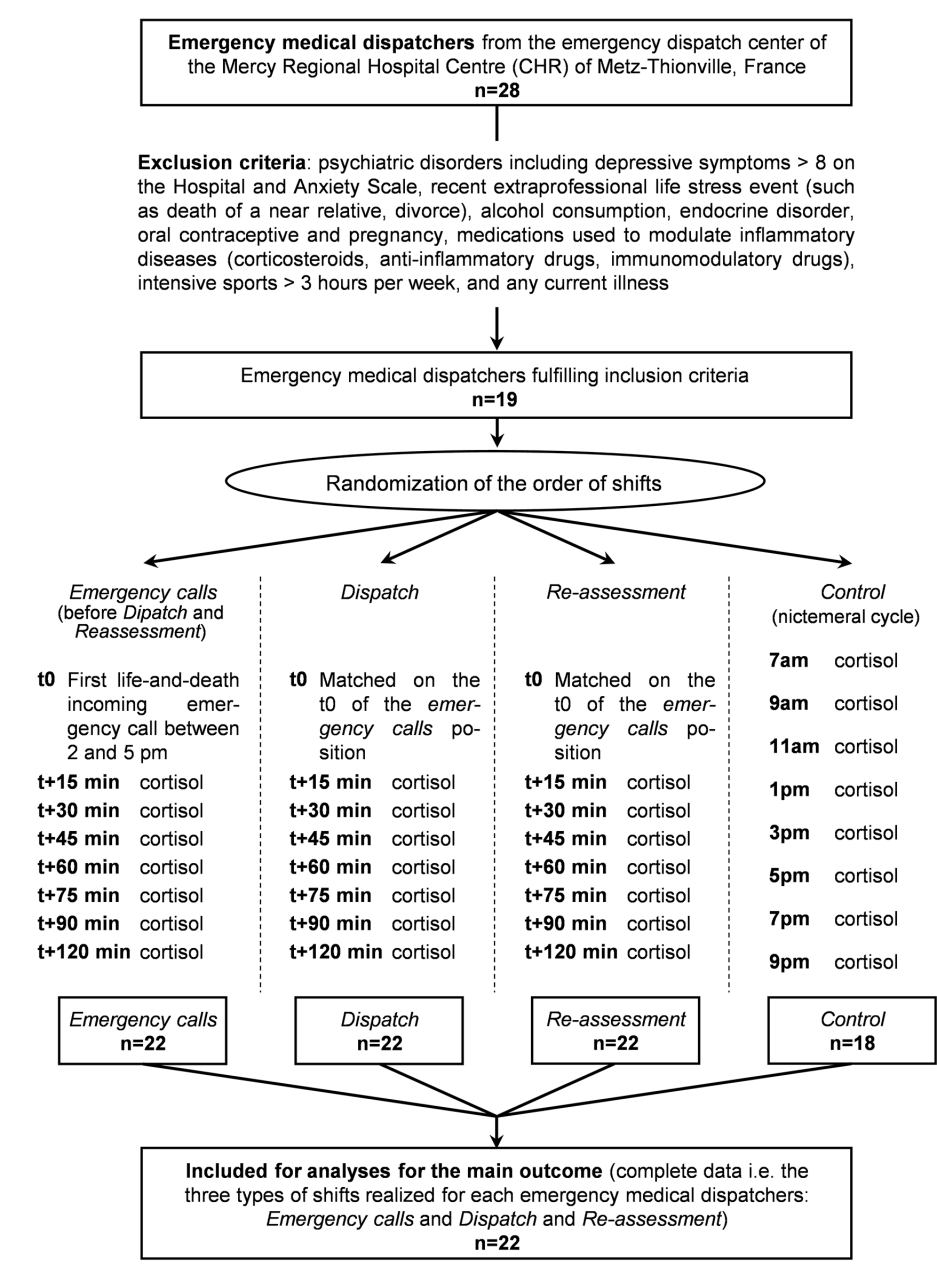
Flow chart and study design.

We recruited 22 phone operators, out of a potential pool of 28 (Fig 1). We obtained complete data for 22 of the 22 phone operators. They were aged 36.4+/-10.8 years (14 women aged 37.9+/-10.7 years, 8 men aged 33.8+/-11.2 years). Men and women did not differ in age (p = .35) but female emergency medical dispatchers had greater experience: 86% of the women had a >5 year experience as against 25% of the men, p = .004. None of the participants were taking routine medications and none took occasional medications during the study. No participant reported not having lunch before cortisol measurements during shifts.

### Cross-sectional analyses of cortisol

#### Endpoint (+120min) between shifts

**Fig 2:**
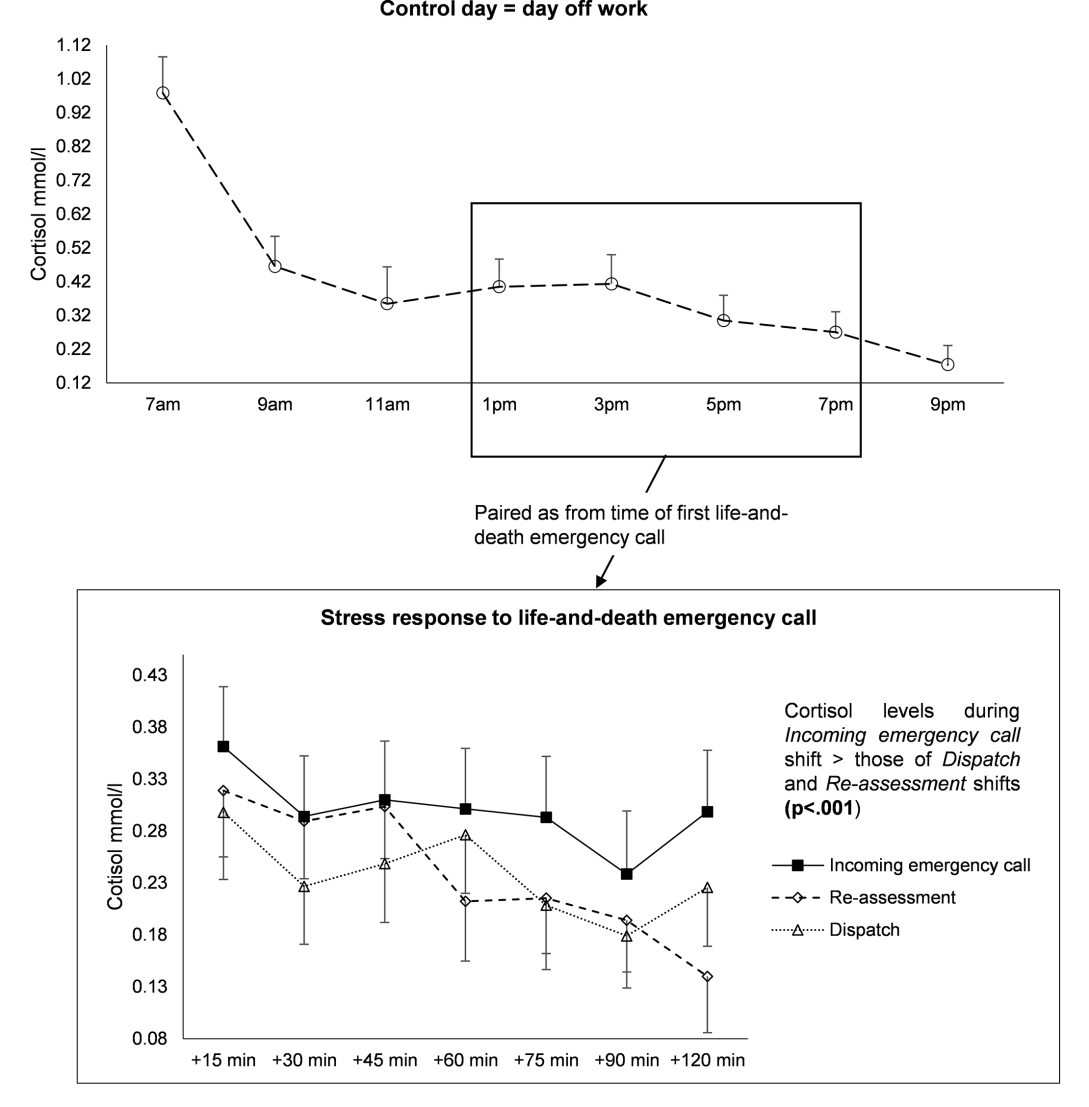
Cortisol levels during control day and paired cortisol levels as from time of first life-and-death incoming emergency call, and during *Dispatch* and *Re-assessment* shifts.

Cortisol levels at endpoint (+120 min) had a tendency (p = 0.11, effect-size 0.65) to be higher for *Incoming emergency call* shift (0.30±0.28 mmol/l) than for *Re-assessment* (0.14±0.23 mmol/l) and *Dispatch* shifts (0.23±0.23 mmol/l) (Fig 2).

#### Baseline and endpoint between control day and shifts

Paired tests showed no differences between control values and shifts (15 minutes after Incoming emergency call, Control day vs *Incoming emergency call*: p = .25; Control day vs *Dispatch* p = .66; Control day vs *Re-assessment*: p = .79; Control day vs *Incoming emergency call*: p = .25; Control day vs *Dispatch*: p = .66; Control day vs *Re-assessment*: p = .79; 2 hours after *Incoming emergency call*, Control day vs *Incoming emergency call*, p = .34; Control day vs *Dispatch* p = .18; Control day vs *Re-assessment*: p = .51).

### Longitudinal evolution of cortisol depending on shift

#### Comparison within shifts

For *Incoming emergency call* shift, cortisol values did not differ from baseline (+15min) except for +90min (p = 0.02). For *Dispatch* shift, the kinetic seemed comparable to *Incoming emergency call* shift with only significant difference at +90min (p = 0.02) and a tendency at +75min (p = 0.08). For *Re-assessment* shift, cortisol values differed from baseline after +60min (p = 0.02) (+75min, p = 0.05; +90min, p = 0.02; and +120min, p = 0.001).

#### Comparison between shifts

The study of interaction measurement-time x shift confirmed these results with no difference between *Incoming emergency call* and *Dispatch* shifts (p = 0.91) and between *Dispatch* and *Re-assessment* shifts (p = 0.20). However, there was a significant interaction between *Incoming emergency call* and *Re-assessment* shift (p = 0.02).

#### Zero-inflated Poisson random-effects model

A zero-inflated Poisson random-effects model [47,48,49,50] showed that kinetics did not differ between the three types of shift (interaction shift position x time, p = .78). However, when all measurements were taken into account, cortisol levels were higher during the *Incoming emergency call* shift than during the *Dispatch* (p = .04) and *Re-assessment* shifts (p = .04) (Fig 2). There was a tendency between the *Dispatch* and the *Re-assessment* shifts (p = .095).

**Therefore**, according to results of all those longitudinal analyses, *Incoming emergency call* and *Dispatch* shifts had a similar kinetic (p = 0.91) but with higher cortisol values *for Incoming emergency call* shift (p = 0.04). Moreover, *Incoming emergency call* and *Re-assessment* shifts had significant different kinetics (p = 0.02), with an increase of cortisol values for *Incoming emergency call* shift whereas cortisol values decreased for *Re-assessment* shift (p = 0.04).

### Longitudinal evolution of cortisol levels depending on participants’ characteristics

#### Gender

**Fig 3:**
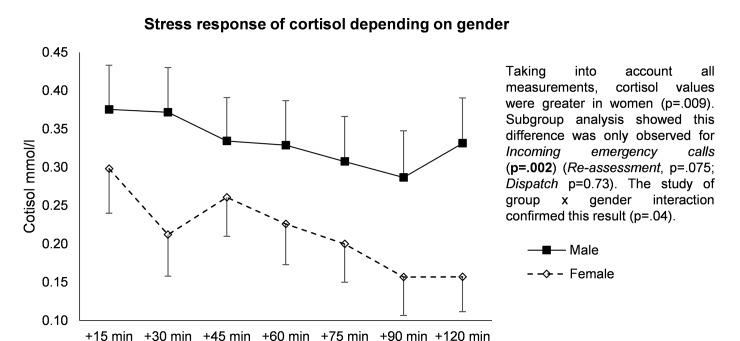
Stress response of cortisol depending on gender during the incoming call shift.

When all measurements were taken into account, cortisol values were greater for men (p = .009) (Fig 3). Subgroup analysis showed that this difference was only observed during the incoming emergency call shift (p = .002) (*Re-assessment*, p = .075; *Dispatch*, p = 0.73). The study of group x gender interaction confirmed this result (p = .04).

#### Age

There were no age-related differences in kinetics.

#### Experience in the EMD unit

An experience-related effect was observed (p = .01) but seemed to be dependent on gender. This was confirmed by multivariate analysis with experience and gender as covariates (p = .21).

#### Perceived stress

The kinetics of cortisol levels increased with greater perceived stress, overall (p < .001) and for each shift (*Incoming emergency call*, p = .02; *Dispatch*, p = .03; *Re-assessment*, p < .001).

#### Severity of incoming emergency call

**Fig 4:**
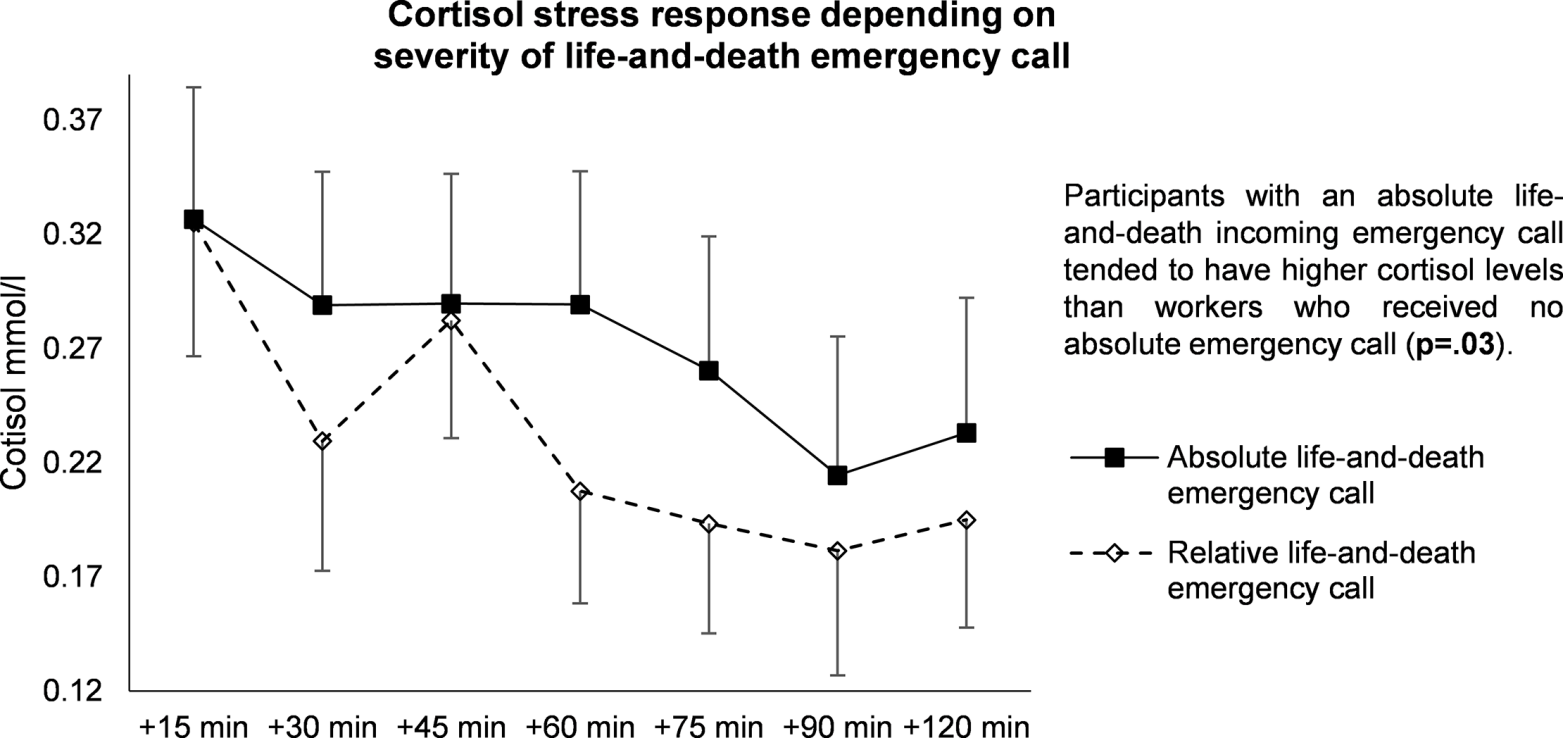
Stress response of cortisol depending on severity of life-and-death emergency oncoming call.

The kinetics of cortisol levels in response to incoming emergency calls was greater when the call was an absolute emergency (p = .03 vs relative absolute incoming emergency call). In particular, participants dealing with an absolute emergency tended to have greater values of cortisol during the last four measurement times compared to workers who had no absolute emergency calls (p = .09) (Fig 4).

#### Subsequent absolute incoming emergency call

**Fig 5:**
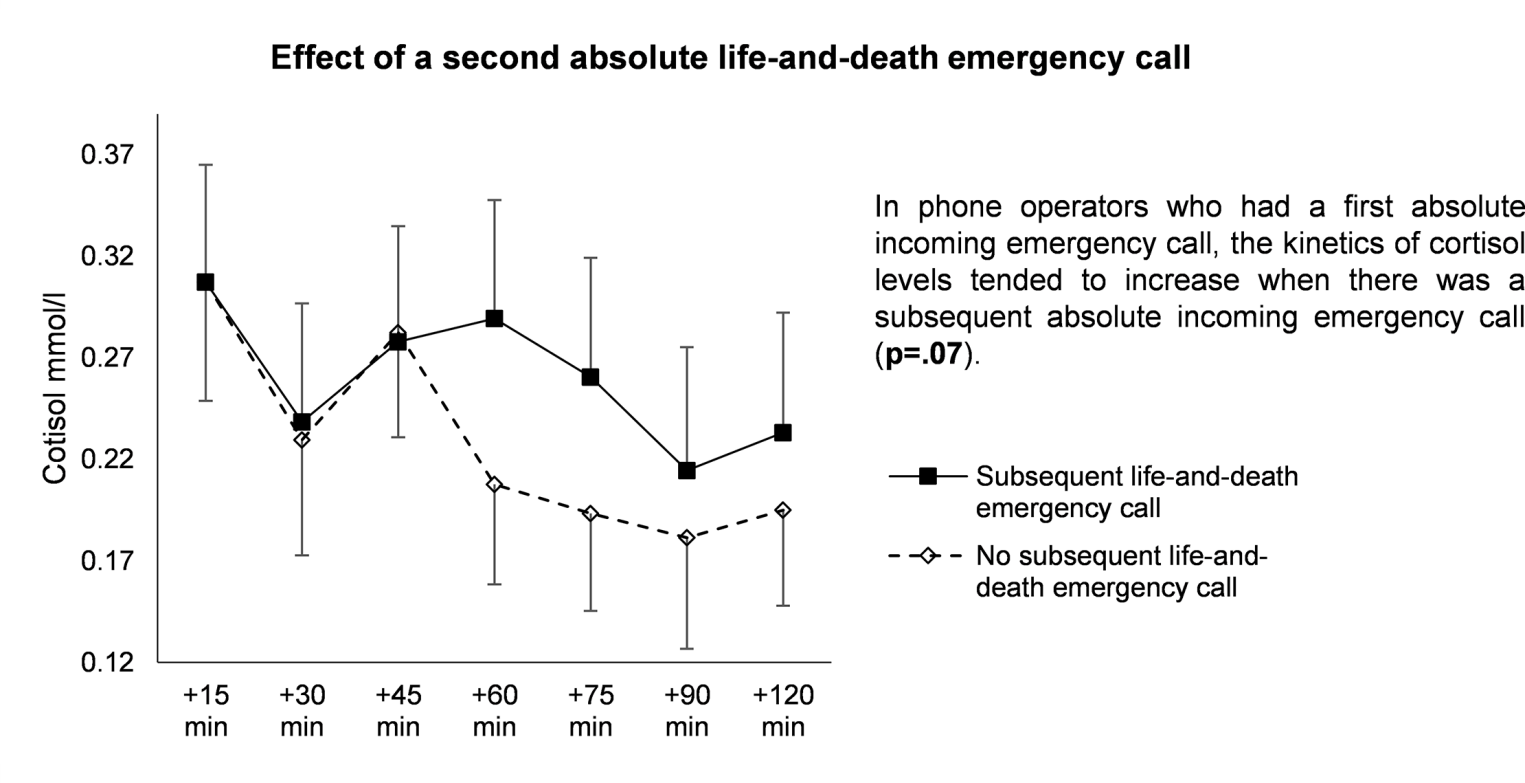
Effects of a subsequent absolute incoming emergency call after beginning of measurements.

In phone operators who had a first absolute incoming emergency call, the kinetics of cortisol values tended to increase when there was a subsequent absolute incoming emergency call (p = .07) (Fig 5).

## Discussion

The main findings to emerge from our study are that cortisol is a relevant biomarker of stress in emergency medical dispatchers, with higher values being recorded during the *Incoming emergency call* shift. Cortisol values were linked with perceived stress and identified stressful events (severity of incoming emergency calls or effects of a subsequent absolute incoming emergency call). We also observed relationships between cortisol levels and sociodemographic variables such as gender and experience.

### Cortisol is a relevant biomarker of stress

In our study, the relationships between cortisol levels and perceived stress confirmed that cortisol is a relevant biomarker of stress in the workplace [22]. Emergency medical dispatchers are particularly at risk of stress because of the combination of witnessing medical distress [6] and the constraints of a call center [51]. There are usually strenuous demands by the employers and not much flexibility allowed for the employees [51]. Chronic stress can lead to basal hypocortisolism characterized by a flattened cortisol rhythm with dramatically smoothed production of glucocorticoids [52]. In our study, the nictemeral cycle of cortisol on the control day was normal. Chronic stress at work can also lead to hyporeactivity [53]. However, we report here for the first time that the *Incoming emergency call* shift is the most stressful period, with phone operators having higher cortisol values than at other times when calls had already been dealt with and decisions already made without any direct contact with callers. In contrast, in some patients with post-traumatic stress disorders a stressful event induces hyporeactivity [54]. This confirms that our phone operators reacted to stressful stimuli but did not experience a biologically overloaded stressful experience. Identifying stressful events may be crucial for safety practice.

### Identification of stressful events

In our study, the *Incoming emergency call* shift induced elevated levels of cortisol, depending on the severity of the emergency call. We also observed a cumulative effect of a subsequent absolute emergency incoming call. Our results bear similarity to the increased morbidity rates described in subsequent patients after surgeons had experienced a patient’s death during emergency surgery [55]. In our study, the potential for residual effects of the *Incoming emergency call* shift need to be investigated, and the consequences of stressful events identified for both patients and phone operators. Phone operators often deal with many absolute emergencies during the same shift, which may produce substantial hypersecretion of cortisol. A succession of days with elevated levels of cortisol can lead to serious diseases such as metabolic disorders including type two diabetes, and also depression and psychiatric disorders [22]. The impact of night shifts can also contribute to stress and to the alteration of the nictemeral cycle of cortisol, which can also lead to an increased morbidity and mortality [16,22]. Thus, the possibility of long-term adverse effects raises the question of the management of stress at work. Further studies could investigate the length of shifts and introduce rotations during the day to reduce the impact of daily stress.

### Sociodemographic variables such as gender and experience

The phone operators included in this study were representative of the overall population of emergency medical dispatchers. There was a greater increase in cortisol levels in response to stress in the men than in the women, as reported elsewhere [56] Another report showed that experienced individuals had a lower increase in cortisol levels and faster recovery compared to those without experience [57]. Although we did not demonstrate an effect of experience, that the longer experience of the female emergency medical dispatchers may have affected the results. Preventive strategies at the workplace could therefore be personalized by taking into account gender specificity and experience.

### Limitations

According to the sample size estimation, the estimated size effect was around 0.7. It is interesting to observe that all results showed an effect size at 0.66 underlying the fact that we lack statistical power for the primary outcome. However, additional exploratory analyses demonstrated significant results. This study has certain limitations. The sample size was small but each individual was their own control, which limited inter-individual cortisol variations. In addition, the high participating rate (75%) limited selection bias. Cortisol values are reported to depend on season, but sampling was conducted in a limited timeframe to limit intra-individual variations. Although cortisol levels are influenced by several factors such as nutritional intake and physical activity [21,29], our study design, which included specific measurements at work, limited the potential effect of these major confounders. Saliva sampling every 15 minutes for 2 hours, starting 15 minutes after the first life-and-death incoming emergency call, were conducted only during the three types of shifts (*Incoming emergency call*, *Dispatch* and *Re-assessment*) and not during the at-home control day because eight measurements every two hours had already been performed to provide a nictemeral cycle of cortisol. Finally, our findings are not necessarily applicable elsewhere because organizational procedures may differ between emergency medical dispatchers. Further analyses should focus on relationships between cortisol secretion and personality traits [58] or other biomarkers of stress [41,59,60,61].

## Conclusion

The shift on which phone operators received incoming calls was the most stressful. Cortisol levels were highest during this period, varying with perceived stress, severity of the emergency call, and the occurrence of a subsequent absolute incoming emergency call. The increase in cortisol levels was greater in the men than in the women during this shift. Preventive strategies in the workplace could be personalized by taking into account gender specificity.

## Supporting information

S1 Appendix CONSORT checklist. (DOCX)

S1 Protocol Study protocol in French. (DOCX)

S2 Protocol Study protocol in English. (DOCX)

S1 Database Titles of columns are written without abbreviations. 999 = missing data.
(XLSX)

## References

[1] Bhui KS, Dinos S, Stansfeld SA, White PD (2012) A synthesis of the evidence for managing stress at work: a review of the reviews reporting on anxiety, depression, and absenteeism. J Environ Public Health 2012: 515874 doi: 10.1155/2012/515874 22496705 10.1155/2012/515874 PMC3306941

[2] Ronda E, Alvarez-Dardet C (2000) Work and health: a call for action. J Epidemiol Community Health 54: 481. 10.1136/jech.54.7.481 PMC1731705 10846188

[3] Bejean S, Sultan-Taieb H (2005) Modeling the economic burden of diseases imputable to stress at work. Eur J Health Econ 6: 16–23. doi: 10.1007/s10198-004-0251-4 15452742 10.1007/s10198-004-0251-4

[4] Choron G, Dutheil F, Lesage FX (2016) Are nurses burned out? Int J Nurs Stud 58: 80–81. doi: 10.1016/j.ijnurstu.2016.02.002 27087300 10.1016/j.ijnurstu.2016.02.002

[5] Nakajima Y, Takahashi T, Shetty V, Yamaguchi M (2012) Patterns of salivary cortisol levels can manifest work stress in emergency care providers. J Physiol Sci 62: 191–197. doi: 10.1007/s12576-012-0197-8 22350686 10.1007/s12576-012-0197-8 PMC5111549

[6] Weibel L, Gabrion I, Aussedat M, Kreutz G (2003) Work-related stress in an emergency medical dispatch center. Ann Emerg Med 41: 500–506. doi: 10.1067/mem.2003.109 12658250 10.1067/mem.2003.109

[7] Trousselard M, Dutheil F, Naughton G, Cosserant S, Amadon S, Duale C, et al (2016) Stress among nurses working in emergency, anesthesiology and intensive care units depends on qualification: a Job Demand-Control survey. Int Arch Occup Environ Health 89: 221–229. doi: 10.1007/s00420-015-1065-7 26112796 10.1007/s00420-015-1065-7

[8] Roth SG, Moore CD (2009) Work-family fit: the impact of emergency medical services work on the family system. Prehosp Emerg Care 13: 462–468. doi: 10.1080/10903120903144791 19731158 10.1080/10903120903144791

[9] Bellagamba G, Gionta G, Senergue J, Beque C, Lehucher-Michel MP (2015) Organizational factors impacting job strain and mental quality of life in emergency and critical care units. Int J Occup Med Environ Health 28: 357–367. doi: 10.13075/ijomeh.1896.00121 26182930 10.13075/ijomeh.1896.00121

[10] Vaillancourt C, Charette ML, Bohm K, Dunford J, Castren M (2011) In out-of-hospital cardiac arrest patients, does the description of any specific symptoms to the emergency medical dispatcher improve the accuracy of the diagnosis of cardiac arrest: a systematic review of the literature. Resuscitation 82: 1483–1489. doi: 10.1016/j.resuscitation.2011.05.020 21704442 10.1016/j.resuscitation.2011.05.020

[11] Moller TP, Ersboll AK, Tolstrup JS, Ostergaard D, Viereck S, Overton J, et al (2015) Why and when citizens call for emergency help: an observational study of 211,193 medical emergency calls. Scand J Trauma Resusc Emerg Med 23: 88 doi: 10.1186/s13049-015-0169-0 26530307 10.1186/s13049-015-0169-0 PMC4632270

[12] Shao MF, Chou YC, Yeh MY, Tzeng WC (2010) Sleep quality and quality of life in female shift-working nurses. Journal of Advanced Nursing 66: 1565–1572. doi: 10.1111/j.1365-2648.2010.05300.x 20492021 10.1111/j.1365-2648.2010.05300.x

[13] Montassier E, Labady J, Andre A, Potel G, Berthier F, Jenvrin J, et al (2015) The effect of work shift configurations on emergency medical dispatch center response. Prehosp Emerg Care 19: 254–259. doi: 10.3109/10903127.2014.959217 25295382 10.3109/10903127.2014.959217

[14] Rugless MJ, Taylor DM (2011) Sick leave in the emergency department: staff attitudes and the impact of job designation and psychosocial work conditions. Emerg Med Australas 23: 39–45. doi: 10.1111/j.1742-6723.2010.01372.x 21284812 10.1111/j.1742-6723.2010.01372.x

[15] Jones CB (2008) Revisiting nurse turnover costs: adjusting for inflation. Journal of Nursing Administration 38: 11–18. doi: 10.1097/01.NNA.0000295636.03216.6f 18157000 10.1097/01.NNA.0000295636.03216.6f

[16] Fekedulegn D, Burchfiel CM, Violanti JM, Hartley TA, Charles LE, Andrew ME, et al (2012) Associations of long-term shift work with waking salivary cortisol concentration and patterns among police officers. Ind Health 50: 476–486. 23047078 10.2486/indhealth.2012-0043 PMC4685453

[17] Ulhoa MA, Marqueze EC, Kantermann T, Skene D, Moreno C (2011) When does stress end? Evidence of a prolonged stress reaction in shiftworking truck drivers. Chronobiol Int 28: 810–818. doi: 10.3109/07420528.2011.613136 22080787 10.3109/07420528.2011.613136

[18] Hufnagel C, Chambres P, Bertrand PR, Dutheil F (2017) The Need for Objective Measures of Stress in Autism. Front Psychol 8: 64 doi: 10.3389/fpsyg.2017.00064 28191002 10.3389/fpsyg.2017.00064 PMC5269614

[19] Doom JR, Gunnar MR (2013) Stress physiology and developmental psychopathology: past, present, and future. Dev Psychopathol 25: 1359–1373. doi: 10.1017/S0954579413000667 24342845 10.1017/S0954579413000667 PMC3869040

[20] Hellhammer DH, Wust S, Kudielka BM (2009) Salivary cortisol as a biomarker in stress research. Psychoneuroendocrinology 34: 163–171. doi: 10.1016/j.psyneuen.2008.10.026 19095358 10.1016/j.psyneuen.2008.10.026

[21] Kirschbaum C, Hellhammer DH (1989) Salivary cortisol in psychobiological research: an overview. Neuropsychobiology 22: 150–169. doi: 118611 2485862 10.1159/000118611

[22] Bozovic D, Racic M, Ivkovic N (2013) Salivary cortisol levels as a biological marker of stress reaction. Med Arch 67: 374–377. 24601177 10.5455/medarh.2013.67.374-377

[23] Woda A, Picard P, Dutheil F (2016) Dysfunctional stress responses in chronic pain. Psychoneuroendocrinology 71: 127–135. doi: 10.1016/j.psyneuen.2016.05.017 27262345 10.1016/j.psyneuen.2016.05.017

[24] Dutheil F, Chambres P, Hufnagel C, Auxiette C, Chausse P, Ghozi R, et al (2015) 'Do Well B.': Design Of WELL Being monitoring systems. A study protocol for the application in autism. BMJ Open 5: e007716 doi: 10.1136/bmjopen-2015-007716 25710916 10.1136/bmjopen-2015-007716 PMC4336464

[25] Kirschbaum C, Kudielka BM, Gaab J, Schommer NC, Hellhammer DH (1999) Impact of gender, menstrual cycle phase, and oral contraceptives on the activity of the hypothalamus-pituitary-adrenal axis. Psychosom Med 61: 154–162. 10204967 10.1097/00006842-199903000-00006

[26] Knorr U, Vinberg M, Kessing LV, Wetterslev J (2010) Salivary cortisol in depressed patients versus control persons: a systematic review and meta-analysis. Psychoneuroendocrinology 35: 1275–1286. doi: 10.1016/j.psyneuen.2010.04.001 20447770 10.1016/j.psyneuen.2010.04.001

[27] Meinlschmidt G, Heim C (2005) Decreased cortisol awakening response after early loss experience. Psychoneuroendocrinology 30: 568–576. doi: 10.1016/j.psyneuen.2005.01.006 15808926 10.1016/j.psyneuen.2005.01.006

[28] Badrick E, Bobak M, Britton A, Kirschbaum C, Marmot M, Kumari M (2008) The relationship between alcohol consumption and cortisol secretion in an aging cohort. Journal of Clinical Endocrinology and Metabolism 93: 750–757. doi: 10.1210/jc.2007-0737 18073316 10.1210/jc.2007-0737 PMC2266962

[29] Weibel L (2003) [Methodological guidelines for the use of salivary cortisol as biological marker of stress]. Presse Med 32: 845–851. 12870390

[30] Liening SH, Stanton SJ, Saini EK, Schultheiss OC (2010) Salivary testosterone, cortisol, and progesterone: two-week stability, interhormone correlations, and effects of time of day, menstrual cycle, and oral contraceptive use on steroid hormone levels. Physiol Behav 99: 8–16. doi: 10.1016/j.physbeh.2009.10.001 19833145 10.1016/j.physbeh.2009.10.001

[31] Kudielka BM, Hellhammer DH, Wust S (2009) Why do we respond so differently? Reviewing determinants of human salivary cortisol responses to challenge. Psychoneuroendocrinology 34: 2–18. doi: 10.1016/j.psyneuen.2008.10.004 19041187 10.1016/j.psyneuen.2008.10.004

[32] Granger DA, Hibel LC, Fortunato CK, Kapelewski CH (2009) Medication effects on salivary cortisol: tactics and strategy to minimize impact in behavioral and developmental science. Psychoneuroendocrinology 34: 1437–1448. doi: 10.1016/j.psyneuen.2009.06.017 19632788 10.1016/j.psyneuen.2009.06.017

[33] Gatti R, De Palo EF (2011) An update: salivary hormones and physical exercise. Scand J Med Sci Sports 21: 157–169. doi: 10.1111/j.1600-0838.2010.01252.x 21129038 10.1111/j.1600-0838.2010.01252.x

[34] Torpy DJ, Ho JT (2007) Value of free cortisol measurement in systemic infection. Hormone and Metabolic Research 39: 439–444. doi: 10.1055/s-2007-980200 17578761 10.1055/s-2007-980200

[35] Collins KJ, Few JD (1979) Secretion and metabolism of cortisol and aldosterone during controlled hyperthermia. J Physiol 292: 1–14. 490331 10.1113/jphysiol.1979.sp012834 PMC1280841

[36] Yang Y, Koh D, Ng V, Lee FC, Chan G, Dong F, et al (2001) Salivary cortisol levels and work-related stress among emergency department nurses. Journal of Occupational and Environmental Medicine 43: 1011–1018. 11765672 10.1097/00043764-200112000-00003

[37] Looser RR, Metzenthin P, Helfricht S, Kudielka BM, Loerbroks A, Thayer JF, et al (2010) Cortisol is significantly correlated with cardiovascular responses during high levels of stress in critical care personnel. Psychosomatic Medicine 72: 281–289. doi: 10.1097/PSY.0b013e3181d35065 20190125 10.1097/PSY.0b013e3181d35065

[38] Metzenthin P, Helfricht S, Loerbroks A, Terris DD, Haug HJ, Subramanian SV, et al (2009) A one-item subjective work stress assessment tool is associated with cortisol secretion levels in critical care nurses. Preventive Medicine 48: 462–466. doi: 10.1016/j.ypmed.2009.02.001 19457727 10.1016/j.ypmed.2009.02.001

[39] Backe EM, Kaul G, Klussmann A, Liebers F, Thim C, Massbeck P, et al (2009) Assessment of salivary cortisol as stress marker in ambulance service personnel: comparison between shifts working on mobile intensive care unit and patient transport ambulance. International Archives of Occupational and Environmental Health 82: 1057–1064. doi: 10.1007/s00420-009-0428-3 19533163 10.1007/s00420-009-0428-3

[40] Dutheil F, Boudet G, Perrier C, Lac G, Ouchchane L, Chamoux A, et al (2012) JOBSTRESS study: comparison of heart rate variability in emergency physicians working a 24-hour shift or a 14-hour night shift—a randomized trial. Int J Cardiol 158: 322–325. doi: 10.1016/j.ijcard.2012.04.141 22608270 10.1016/j.ijcard.2012.04.141

[41] Dutheil F, Trousselard M, Perrier C, Lac G, Chamoux A, Duclos M, et al (2013) Urinary Interleukin-8 Is a Biomarker of Stress in Emergency Physicians, Especially with Advancing Age—The JOBSTRESS* Randomized Trial. PLoS One 8: e71658 doi: 10.1371/journal.pone.0071658 23977105 10.1371/journal.pone.0071658 PMC3747272

[42] Nakamura Y, Walker BR, Ikuta T (2016) Systematic review and meta-analysis reveals acutely elevated plasma cortisol following fasting but not less severe calorie restriction. Stress 19: 151–157. doi: 10.3109/10253890.2015.1121984 26586092 10.3109/10253890.2015.1121984

[43] Lesage FX, Berjot S (2011) Validity of occupational stress assessment using a visual analogue scale. Occup Med 61: 434–436. 10.1093/occmed/kqr037 21505089

[44] Lac G, Dutheil F, Brousse G, Triboulet-Kelly C, Chamoux A (2012) Saliva DHEAS changes in patients suffering from psychopathological disorders arising from bullying at work. Brain and Cognition 80: 277–281. doi: 10.1016/j.bandc.2012.07.007 22940752 10.1016/j.bandc.2012.07.007

[45] Miller R, Plessow F, Rauh M, Groschl M, Kirschbaum C (2013) Comparison of salivary cortisol as measured by different immunoassays and tandem mass spectrometry. Psychoneuroendocrinology 38: 50–57. doi: 10.1016/j.psyneuen.2012.04.019 22641005 10.1016/j.psyneuen.2012.04.019

[46] Cohen J (1988) Statistical power analysis for the behavioral sciences New Jersey: Lawrence Erlbaum.

[47] Hall DB (2000) Zero-inflated Poisson and binomial regression with random effects: a case study. Biometrics 56: 1030–1039. 11129458 10.1111/j.0006-341x.2000.01030.x

[48] Hasan MT, Sneddon G, Ma R (2009) Pattern-mixture zero-inflated mixed models for longitudinal unbalanced count data with excessive zeros. Biom J 51: 946–960. doi: 10.1002/bimj.200900093 20029895 10.1002/bimj.200900093

[49] Min Y, Agresti A (2005) Random effect models for repeated measures of zero-inflated count data. Statistical Modelling 5: 1–19.

[50] Lambert D (1992) Zero-Inflated Poisson Regression, With an Application to Defects in Manufacturing. Technometrics 34: 1–14.

[51] Charbotel B, Croidieu S, Vohito M, Guerin AC, Renaud L, Jaussaud J, et al (2009) Working conditions in call-centers, the impact on employee health: a transversal study. Part II. Int Arch Occup Environ Health 82: 747–756. doi: 10.1007/s00420-008-0351-z 18704480 10.1007/s00420-008-0351-z

[52] Heuser I, Lammers CH (2003) Stress and the brain. Neurobiology of Aging 24 Suppl 1: S69–76; discussion S81-62. 12829112 10.1016/s0197-4580(03)00048-4

[53] Teixeira RR, Diaz MM, Santos TV, Bernardes JT, Peixoto LG, Bocanegra OL, et al (2015) Chronic stress induces a hyporeactivity of the autonomic nervous system in response to acute mental stressor and impairs cognitive performance in business executives. PLoS One 10: e0119025 doi: 10.1371/journal.pone.0119025 25807003 10.1371/journal.pone.0119025 PMC4373764

[54] Meewisse ML, Reitsma JB, de Vries GJ, Gersons BP, Olff M (2007) Cortisol and post-traumatic stress disorder in adults: systematic review and meta-analysis. Br J Psychiatry 191: 387–392. doi: 10.1192/bjp.bp.106.024877 17978317 10.1192/bjp.bp.106.024877

[55] Goldstone AR, Callaghan CJ, Mackay J, Charman S, Nashef SA (2004) Should surgeons take a break after an intraoperative death? Attitude survey and outcome evaluation. BMJ 328: 379 doi: 10.1136/bmj.37985.371343.EE 14734519 10.1136/bmj.37985.371343.EE PMC341385

[56] Lovallo WR, Farag NH, Vincent AS (2010) Use of a resting control day in measuring the cortisol response to mental stress: diurnal patterns, time of day, and gender effects. Psychoneuroendocrinology 35: 1253–1258. doi: 10.1016/j.psyneuen.2010.02.015 20233640 10.1016/j.psyneuen.2010.02.015 PMC2896983

[57] Meyer VJ, Lee Y, Bottger C, Leonbacher U, Allison AL, Shirtcliff EA (2015) Experience, cortisol reactivity, and the coordination of emotional responses to skydiving. Front Hum Neurosci 9: 138 doi: 10.3389/fnhum.2015.00138 25859199 10.3389/fnhum.2015.00138 PMC4373275

[58] Costa PT Jr., McCrae RR (1997) Stability and change in personality assessment: the revised NEO Personality Inventory in the year 2000. J Pers Assess 68: 86–94. doi: 10.1207/s15327752jpa6801_7 9018844 10.1207/s15327752jpa6801_7

[59] Boudet G, Walther G, Courteix D, Obert P, Lesourd B, Pereira B, et al (2016) Paradoxical dissociation between heart rate and heart rate variability following different modalities of exercise in individuals with metabolic syndrome: The RESOLVE study. Eur J Prev Cardiol. 10.1177/2047487316679523 27856807

[60] Dutheil F, Perrier C, Boudet G, Lac G, Chamoux A, Duclos M, et al Poster: Comparison of heart rate variability of two systems of shifts: 14 hours versus 24 hours among emergency physicians.; 2011 6, 2011; Paris, France.

[61] Lac G, Dutheil F, Brousse G, Triboulet-Kelly C, Chamoux A (2012) Saliva DHEAS changes in patients suffering from psychopathological disorders arising from bullying at work. Brain Cogn 80: 277–281. doi: 10.1016/j.bandc.2012.07.007 22940752 10.1016/j.bandc.2012.07.007

