# Supplementary material for: Stress and salivary cortisol in emergency medical dispatchers: A randomized shifts control trial
Source: PLoS One. 2017 May 15;12(5):e0177094. doi: 10.1371/journal.pone.0177094 (PMC5432068; doi:10.1371/journal.pone.0177094)
Supplement: S1 Protocol — (DOCX) [file pone.0177094.s002.docx]

**Protocole de Recherche Clinique**

Avis favorable du CPP le…/…/…

Autorisation de l’ANSM le …/…/…

Version N**°2.3 du**  13/03/2014

N° IDRCB : 2013-A01725-40

**Etude descriptive du stress biologique et du stress percu chez les assistants de regulation médicale et les medecins lors du traitement d’un appel au centre 15**

**Promoteur :**

CHR Metz-Thionville

Hôpital de Mercy

1, allée du château - CS 45001

57085 Metz - cedex 03

**Investigateur principal :**

Dr Michel AUSSEDAT

Centre 15

Hôpital de Mercy

1, allée du château - CS 45001

57085 Metz - cedex 03

Téléphone : 03 87 55 36 22

Adresse électronique : m.aussedat@chr-metz-thionville.fr

**PRINCIPAUX CORRESPONDANTS**

**Investigateur principal :**

Dr Michel AUSSEDAT

Centre 15 - Hôpital de Mercy

1, allée du château - CS 45001

57085 Metz - cedex 03

Téléphone : 03 87 55 36 22

Adresse électronique : m.aussedat@chr-metz-thionville.fr

**Chef de projet :**

Sarah BEDINI

6 rue des cygnes

57360 Hagondange

Téléphone : 07 77 28 28 36

Adresse électronique : sarah.bedini3@gmail.com

**Responsable de la recherche agissant pour le compte du promoteur et autorisé à signer le protocole et ses modifications éventuelles au nom du promoteur :**

Véronique ANATOLE TOUZET

Directrice Générale

CHR Metz-Thionville

Hôpital de Mercy

1, allée du château - CS 45001

57085 Metz - cedex 03

**Plateforme d’Appui à la Recherche Clinique (PARC) du CHR Metz-Thionville :**

Médecin coordonnateur :

Docteur Christophe GOETZ

Hôpital de Mercy,

1 allée du Château - CS 45001

57085 METZ cedex 03

Tél : 03 87 55 37 46

Mail : c.goetz@chr-metz-thionville.fr

Historique des mises à jour du protocole

| Version | Date | Raison de la mise à jour |
| --- | --- | --- |
|  |  |  |
|  |  |  |
|  |  |  |
|  |  |  |

**SOMMAIRE**

**1. RESUME DU PROTOCOLE**

**2. Justification scientifique et description générale de la recherche**

**3. Objectifs de la recherche**

**4. Conception de la recherche**

**5. Sélection et exclusion des personnes de la recherche :**

**6. DEScription de l'exposition**

**7. Evaluation de la sécurité**

**8. Statistiques**

**9. Droit d'accès aux données et documents source.**

**10. Contrôle et assurance de la qualité.**

**11. Considérations éthiques ET REGLEMENTAIRES**

**12. Traitement des données et conservation des documents et des données relatives à la recherche.**

**13. Financement**

**14. Règles relatives à la publication.**

**15. Liste des annexes**

**16. REFERENCES BIBLIOGRAPHIQUES**

**1. RESUME DU PROTOCOLE**

| Titre | *Etude descriptive du stress biologique et du stress perçu chez les assistants de régulation médical et les médecins lors du traitement d’un appel au centre 15* |
| --- | --- |
| Promoteur | CHR Metz-Thionville  Hôpital de Mercy  1, allée du château - CS 45001  57085 Metz - cedex 03 |
| Investigateur principal | *Dr Michel AUSSEDAT* |
| Version du protocole | *version N° 2.2 du 28/01/2014* |
| Justification / contexte | *Il est maintenant prouvé que le stress au travail est en cause dans bon nombre de pathologies allant du syndrome métabolique, responsable de complications cardiovasculaires, à la dépression.*  *Les personnels exerçant dans les services d’accueil des urgences sont particulièrement exposés au stress dans le cadre du travail. A ce jour, quelques études ont été réalisées dans le cadre du stress aux urgences chez les médecins et les infirmiers, mais très peu se sont penchées sur le stress en régulation. Après notre revue de la littérature, il s’avère qu’une seule étude à été réalisée, et porte sur le stress lors d’une journée, et du stress aigu lors d’un appel (1).* |
| Objectif Principal | *Décrire le stress biologique (cortisolémie) et perçu (EVA) chez les ARM (assistants de régulation médicale) et les médecins lors du traitement d’un appel au centre 15* |
| Objectifs Secondaires | *Comparer le stress des ARM et objectiver une différence entre les différents postes*  *Etudier une corrélation entre stress perçu et stress biologique*  *Etudier la corrélation entre gravité de la situation téléphonique et stress* |
| Critère de Jugement Principal | *Stress biologique : Sécrétion du cortisol salivaire (aire sous la courbe)* |
| Critères de Jugement Secondaires | *Stress perçu : questionnaire SPPN (Stress Professionnel Positif et Négatif) et EVA* |
| Méthodologie / Schéma de l’etude | *Etude descriptive interventionnelle longitudinale monocentrique* |
| Critères d’Inclusion des Sujets | - *Personnel médical et paramédical du centre 15 (permanenciers, médecins urgentistes, médecins libéraux)* - *Ayant reçu une information sur l’étude* - *Ayant signé un consentement de participation à l’étude* - *Affiliation obligatoire à un régime de sécurité sociale* |
| Critères de Non-Inclusion des Sujets | - *Ethylisme chronique* - *Les femmes sous traitement oestroprogestatifs* - *Femmes enceintes* - *Les sportifs de haut niveau* - *Les personnes sous traitement médicamenteux à base de stéroïdes* - *Les personnes suivies pour désordres psychiatriques (en particulier la dépression)* - *Fièvre le jour du prélèvement* - *Participants volontaires porteurs de pathologies endocriniennes* |
| Procédures | *Recueil salivaire par salivette Biomnis ou Sarstedt. Le premier prélèvement (P0) sera réalisé lors du premier appel dans le créneau horaire 10h-13h, puis une série de prélèvements sera réalisée ensuite chez les deux autres permanenciers, puis chez le médecin généraliste ou urgentiste à qui sera passé l’appel.*  *Chez les ARM, trois séries de prélèvements seront réalisées dans le cadre de leur travail. Chaque permanencier aura une série de prélèvements quand il sera en poste appel, une série de prélèvements en poste déploiement de moyen, puis une série de prélèvements en poste dit bilan.*  *Les médecins étant toujours au même poste, une seule série de prélèvements sera réalisée au travail.*  *Les prélèvements seront réalisés toutes les 15 minutes pendant 1h30 à partir de T0 puis un dernier prélèvement sera effectué 2h après l’appel.*  *Afin de pouvoir améliorer l'interprétation des résultats, une série de prélèvements témoins sera réalisée chez les ARM et les médecins lors d'un jour de repos en dehors des lendemains de garde.*  *Soit quatre fois 8 prélèvements par ARM et deux fois 8 prélèvements par médecin.*  *Ils seront ensuite congelés à une température de – 4 degrés puis envoyés au laboratoire du CHR de l’hôpital de Mercy.* |
| Nombre de Participants volontaires | *Il s’agit d’une étude dont l’objectif principal est descriptif et qui peut servir d’étude de faisabilité concernant les objectifs comparatifs (secondaires). Il est prévu d’inclure 20 ARM et 20 médecins.* |
| Durée de la Recherche | *Durée de la période d’inclusion : 1 mois*  *Durée de participation de chaque sujet : quatre fois deux heures pour les ARM et deux fois deux heures pour les médecins.*  *ARM = 4 fois 8 prélèvements / Médecins : 2 fois 8 prélèvements*  *Durée totale de l’étude:6 mois* |
| analyse statistique | *Description de l’échantillon : les variables qualitatives seront décrites en pourcentage, effectif et intervalle de confiance à 95%, les variables quantitatives seront décrites en moyennes et écarts-types ou médiane, étendue et intervalle interquartiles.*  *Description du stress biologique : il sera décrit en calculant la différence entre le pic ou les pics de cortisol observés et le taux de cortisol mesuré au repos (moyennes et écarts-types ou médiane, étendue et intervalle interquartiles).*  *Description du stress perçu : le score de l’EVA sera décrit en termes de moyenne et écart type.*  *A titre exploratoire : comparaison du delta pic/taux de base de cortisol selon les 3 postes des ARM avec un test non paramétrique de Kruskal Wallis, de même selon l’ancienneté des ARM, la gravité de l’appel et le statut du médecin (urgentiste ou libéral).*  *Le score à l’EVA (stress perçu) sera comparé au delta pic/taux de base de cortisol (stress biologique) à l’aide du coefficient de corrélation de Spearmann.* |
| Retombées attendues | *Mesurer de manière objective le stress en service de régulation pour permettre la mise en place de moyens de prévention efficaces.* |

**Liste des abréviations**

|  |  |
| --- | --- |
|  |  |
| ANSM | Agence Nationale de Sécurité des Médicaments et produits de santé |
| ARC | attaché de Recherche Clinique |
| ARM | Assistant de régulation médicale |
| AVC | Accident Vasculaire Cérébral |
| BPC | Bonnes Pratiques Cliniques |
| CNIL | Commission Nationale de l’Informatique et des Libertés |
| CPP | Comité de Protection des Personnes |
| CRF | Case Report Form (cahier d’observation) |
| EIG | Evènement Indésirable Grave |
| EIGI | Effet Indésirable Grave Inattendu |
| ICH | International Conference on Harmonization (Conférence internationale pour l'harmonisation) |
| MCE | Massage cardiaque externe |
| MR | Méthodologie de Référence |
| OAP | Œdème Aigu Pulmonaire |
| SMUR | Service Mobile d'Urgence et de Réanimation |
| SPPN (questionnaire) | Stress Professionnel Positif et Négatif |
| SUSAR | Suspected Unexpected Serious Adverse Reaction |
|  |  |
|  |  |
|  |  |

**2. Justification scientifique et description générale de la recherche :**

**Présentation des données actuelles de la science sur le stress en centre de régulation**

Le stress au travail n’est maintenant plus un concept abstrait. Nous allons voir que de nombreuses études portent sur le sujet, mais que peu ont évalué le stress en centre 15, ou régulation.

Nous ferons ensuite un bref rappel de l’historique du stress et de sa physiopathologie, puis l’intérêt du cortisol dans les études concernant le stress, et enfin le lien entre physiologie et physiopathologie.

**2.1 Le stress au travail**

Depuis maintenant quelques années, le stress devient une préoccupation constante. Notamment dans le monde du travail, car celui-ci prend de plus en plus de place et d’importance dans nos vies. Les entreprises commencent à se rendre compte des pertes engendrées par le stress : maladie, absentéisme, chute de productivité (2). D’après l’agence européenne de sécurité et de santé au travail, le stress au travail toucherait 22 % des travailleurs (chiffres de l’année 2005) et serait responsable de 50 à 60 % de l’absentéisme au travail (3). La prise en charge du stress au travail est aussi un impératif budgétaire, car actuellement en Europe, le coût du stress représenterait 20 milliards d’euros.

L’extension du concept de stress biologique à stress psychique a permis la réalisation d’études sur les conditions de travail (4, 5, 6, 7, 8). Le stress professionnel et ses répercutions sont encore peu explorés, notamment parmi le personnel médical et paramédical (9, 10, 11, 12, 13)

**2.1.1 Le stress au travail dans le milieu des urgences**

Le milieu médical est un milieu socio-professionnel où le stress est permanent, en particulier au service d’accueil des urgences (14, 15, 16, 17, 18, 19, 20, 21, 22, 23) où les médecins et paramédicaux ont une charge de travail importante, représentée par des gardes de 24 heures pour les médecins et des gardes le plus souvent de douze heures pour les paramédicaux (24).

Deux caractéristiques intrinsèques à la tâche de travail sont à l’origine du caractère stressant du métier d’urgentiste : d’une part la dimension d’urgence, c’est-à-dire la pression temporelle qui est stressante en soi et d’autre part, l’impossibilité de prévoir et de planifier la charge de travail (25). Un des aspects du travail d’urgentiste et du personnel paramédical le plus générateur de stress concerne la gestion des appels en régulation au centre 15.

Lors des appels au centre 15, les médecins et les paramédicaux sont confrontés à un stress aigu. Ils doivent mobiliser des ressources psychiques pour recueillir les informations importantes de la prise en charge pour décider ensuite de la nature de l’intervention et des moyens qui seront déployés, de l’ambulance privée à l’envoi d’une équipe médicale de SMUR (service mobile urgences et réanimation).

Des études ayant pour but d’évaluer le niveau de stress professionnel chez des praticiens hospitaliers existent (12, 13, 14). De nombreuses études se sont intéressées depuis quelques années au stress parmi les personnels médicaux et paramédicaux des urgences (17, 18). Ces études se servent maintenant fréquemment du dosage de cortisol salivaire pour objectiver le niveau de stress (22, 25, 26, 27).

**2.1.2 Le stress au travail en régulation**

Les études concernant le stress en régulation sont plus rares. A notre connaissance une seule étude a été réalisée sur le stress des personnels paramédicaux ou permanenciers. Cette étude portait plutôt sur le stress au cours d’une journée, avec une comparaison d’un dosage de cortisol réalisé au cours d’une journée de travail comparé à un dosage de cortisol lors d’une journée de repos (1).

On ne dispose à notre connaissance d’aucune étude sur le stress aigu en régulation chez les permanenciers recevant les appels en régulation. Aucune étude n’a été réalisée sur le stress aigu ou chronique chez les médecins régulateurs.

Aucune étude n’a permis un recueil subjectif et objectif du stress en régulation. Or le stress génère à la fois une réaction physiologique et psychologique de l’organisme. Les approches psychologiques et physiologiques paraissent indissociables et complémentaires à l’évaluation fiable du niveau de réponse au stress.

L’objectif de cette étude est d’évaluer la réponse biologique et psychologique au stress professionnel chez les paramédicaux et les médecins du centre 15. L’évaluation est objective et subjective, reposant sur des mesures biologiques et des questionnaires. L’âge est introduit comme variable explicative en faisant l’hypothèse que l’ancienneté et l’expérience peuvent influencer la réponse au stress.

**2.2 Introduction à la compréhension des mécanismes du stress**

**2.2.1 Historique et définition du stress**

**Historique du stress🡪🡪**

Le stress est un concept ancien, déjà décrit dans le monde antique dans les péripéties des chroniqueurs grecs et latins.

Au XVIIème siècle, stress signifiait en anglais « état de détresse » et renvoyait à l’idée de souffrance, d’oppression, de dureté de la vie, de privation, de fatigue et d’une façon plus générale à la notion d’adversité.

Ce n’est qu’après le XVIIIème siècle que le mot prend son sens moderne de force ou de pression ou encore de forte influence qui agit sur un objet physique ou une personne.

Au cours du XIXème siècle, le raisonnement ajoute l’idée que des conditions de vie agressives (stress) peuvent entraîner des maux physiques et mentaux (strain).

Dans les années 1850, le physiologiste français Claude Bernard, précurseur de la médecine expérimentale, démontre qu’une des propriétés essentielles des êtres vivants est la faculté de maintenir la stabilité de leur milieu interne quels que soient l’environnement extérieur et les modifications qui s’y produisent. (29)

En 1910, un cardiologue rapporte qu’un travail pénible, associé à une lourde responsabilité, est à l’origine de tourments et d’anxiété dont la persistance peut entraîner des problèmes médicaux (Angina pectoris-The Lancet, 1910).

Le neuro-physiologiste américain Walter Bradford Cannon (1871-1945) insiste en 1928 sur le rôle du facteur émotionnel dans le développement des maladies et envisage alors le terme « stress » dans le sens à la fois physiologique et psychologique. Il attire le premier l’attention sur l’importance de la durée de l’agression en différenciant ce qu’il appelle le « fixed stress » et le « variable stress » (30).

Mais c’est Hans Selye qui introduit le premier le mot stress en médecine avec la mise en évidence du syndrome général d’adaptation (31). L’émergence du thème « stress professionnel » dans la littérature est beaucoup plus récent : 1965 (32).

**Définition du stress**

Etymologiquement, le mot stress provient du latin « stringer » : mettre en tension. Le stress répond à plusieurs définitions selon les ouvrages consultés :

Encyclopédie universalis :

Réaction de l’organisme à un agent d’agression ou à un traumatisme quelconque. En fait, « stress » tend, progressivement, à devenir synonyme d’émotion, le sens de ce dernier s’étant très affaibli. Il est employé aussi, dans un sens plus spécifique, pour désigner les tensions engendrées par des conflits internes non résolus ou des situations insolubles et provoquant des états d’anxiété, voire des états névrotiques.

Dictionnaire Larousse :

Réponse de l’organisme aux facteurs d’agression physiologiques et psychologiques ainsi qu’aux émotions (agréables ou désagréables) qui nécessite une adaptation.

Dictionnaire de médecine Flammarion :

Agression contre un organisme vivant ; par extension : réaction biologique et psychologique d’un organisme face à une situation nouvelle de quelque origine qu’elle soit, dangereuse ou agréable.

Dictionnaire Wikipédia :

Le **stress** (issu par l'anglais de l'ancien français *déstresse*) est, en biologie, l'ensemble des réponses d'un organisme soumis à des pressions ou contraintes de la part de son environnement. Ces réponses dépendent toujours de la perception qu'a l'individu des pressions qu'il ressent. Selon la définition médicale, il s'agit d'une séquence complexe d’événements provoquant des réponses physiologiques, psychosomatiques. Par extension tous ces événements sont également qualifiés de stress. Dans le langage courant, on parle de stress positif (*eustress* en anglais) ou négatif (*distress*). Le stress est différent de l'anxiété, celle-ci est une émotion alors que le stress est un mécanisme de réponse pouvant amener différentes émotions, dont l'anxiété.

1. **Défintion selon H. Selye**

Ce concept est introduit dans les années 1930 en individualisant des réactions physiologiques communes à toutes les agressions. L’ensemble des réponses au stress est, d’après Hans Selye, une réponse non spécifique provoquée par un agent agressif physique. Ces réactions sont stéréotypées quel que soit l’agent.

Cette réponse de l’organisme est appelée le syndrome général d’adaptation ou SGA qui évolue dans le temps en trois phases :

- La réaction d’alarme ou d’alerte,
- Le stade de résistance ou d’adaptation
- Le stade d’épuisement.

Selon la définition de Selye, le stress est la « réponse de l’organisme à toute stimulation qui lui est faite. ». Il se caractérise par une réaction physiologique linéaire non spécifique du stresseur.

Le concept de stress a donc évolué au cours des années, de cause déclenchante avec Cannon, il devient effet consécutif avec Selye.

Depuis le début des années 1990, la théorie du stress de Hans Selye a été revue et approfondie par de nombreux scientifiques. La non spécificité de la réponse au stress a été remise en cause avec la démonstration des grandes variations dans les réponses au stress en fonction de l’agent stressant, mais surtout du vécu et de l’expérience de l’individu. Le premier à contredire Hans Selye fut John Mason qui a démontré l’importance de l’activation émotionnelle dans l’intensité des réponses de stress. (34)

Les théories de coping élaborées par les neuropsychologues ont ensuite démontré que l’évaluation cognitive est cruciale dans l’activation émotionnelle et physiologique qui suit. Ces avancées ont permis d’aboutir à la définition actuelle du stress (35) qui prend en compte la forte variabilité interindividuelle observée dans les réponses de stress (36), relevant à la fois du patrimoine génétique (38) et de l’histoire personnelle. (39, 40, 41)

Ainsi le stress peut être considéré comme un concept fondamentalement psychobiologique, les stresseurs agissant par l’intermédiaire de processus cognitifs et émotionnels, tout phénomène mental ayant par essence une correspondance cérébrale et biologique (42).

1. **Définition actuelle**

La définition du stress la plus utilisée est la suivante : « le stress est un état dynamique traduisant un déséquilibre psychophysiologique entre les ressources estimées et les exigences perçues lors de situations contraignantes »(42).

1. **Définition du stress au travail**

Le 8 octobre 2004, un accord européen a donné une définition du stress au travail afin de mieux sensibiliser le monde du travail, de développer des mesures anti-stress et définir la responsabilité de chacun, employeurs ou salariés.

La définition est la suivante : « le stress est un déséquilibre entre les contraintes imposées par l’environnement et la perception des ressources pour y faire face. »

Cette définition complète celle énoncée par Schaufeli en 1998 (43) : « le stress est un état transitoire de désadaptation fonctionnelle dont les symptômes psychiques, physiologiques et comportementaux manifestent une tentative d’adaptation non encore réussie et se révélant à court/moyen terme, le « burn-out » pouvant en être  une étape ultime, à la symptomatologie très diversifiée et se traduisant à long terme par une incapacité totale à agir.

**2.3 Physiologie de la réponse au stress**

Chaque individu réagit de manière différente à un stimulus stressant, selon l’intensité des agents stresseurs et sa capacité à y faire face. Le stress intervient quand existe un déséquilibre entre les exigences perçues et les ressources disponibles. La réponse physiologique à un stress est une réponse d’adaptation permettant une mobilisation accrue des ressources. On parle de pathologie quand il y a rupture d’équilibre entre les capacités d’adaptation du sujet et les exigences de l’environnement.

On distingue deux types de stress : le stress aigu et le stress chronique, qui abouti à la pathologie.

**2.3.1 Le stress aigu :**

C’est la composante adaptative.

De nombreux médiateurs biologiques sont impliqués. (37)

Un évènement isolé, ressenti comme une menace, une situation d’incertitude et d’imprévisibilité, conduit à un stress aigu et à la recherche d’un nouvel équilibre. On distingue trois phases : la phase d’alarme, l’adaptation puis la phase de résistance.

**La phase d’alarme**

Le cerveau est soumis dans cette phase à un élément perturbateur stressant qui stimule l’hypothalamus et qui est responsable d’une double activation des hormones et neuromédiateurs de l’axe du stress, les catécholamines.

- **Périphérique :**

Cette action se fait par l’intermédiaire du système nerveux autonome (SNA) sympathique, les médullosurrénales synthétisent adrénaline et noradrénaline, messagers hormonaux responsables des effets stimulants cardiovasculaires de la réaction de l’organisme soumis à un stress.

Ces catécholamines périphériques activent ensuite le tractus solitaire via le nerf vague puis le locus coeruleus de la région médiane rostrale de la protubérance.

Les neurotransmetteurs (noradrénaline, sérotonine, dopamine) et les peptides (CRH, AVP) agissent dans les minutes qui suivent l’apparition de l’évènement stresseur et cessent aussi rapidement, ce pour favoriser la vigilance, l’évaluation de la situation et la prise de décision.

- **Centrale :**

C’est le locus coeruleus qui active l’hypothalamus en stimulant les régions limbiques et préfrontales riches en récepteurs alpha et béta adrénergiques.

Le locus coeruleus, en interaction avec l’amygdale, a un rôle clé dans l’orchestration des réponses comportementales et biologiques de stress. L’activation de l’amygdale est modulée par ses connexions neuronales, d’une part avec le cortex préfrontal (région fondamentale pour l’évaluation cognitive des informations reçues) et d’autre part avec l’hippocampe (structure clé dans la mémoire des expériences similaires déjà vécues).(44)

La stimulation du locus coeruleus active en particulier le cortex préfrontal par le biais de ses nombreuses projections noradrénergiques. La stimulation de l’amygdale entraîne la libération rapide de neurotransmetteurs tels que la dopamine, l’acétylcholine, la sérotonine et l’adrénaline, et de peptides tels que la corticolibérine (ou CRH : cortisol releasing hormone). (36)


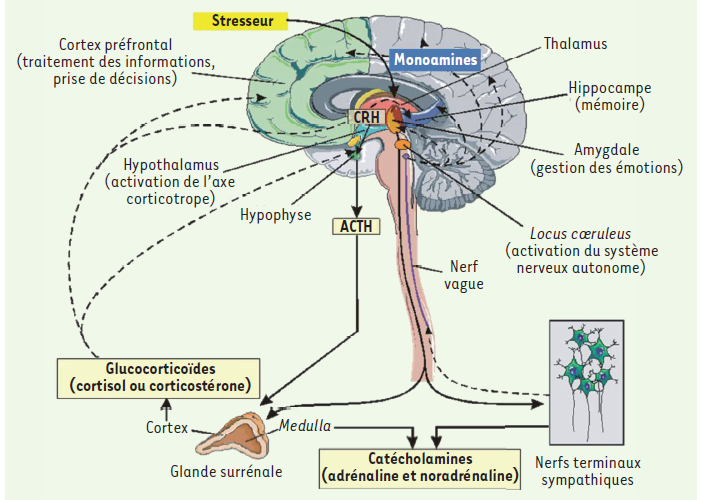


**Figure 1 : Médiateurs biologiques de la réponse au stress.**

**Phase d’adaptation**:

Si le stimulus dure trop longtemps, la mobilisation de l’énergie de l’ensemble du corps est nécessaire ; c’est alors le système endocrinien qui prend le relais.

L’axe corticotrope est activé par la synthèse de CRH dans l’hypothalamus pour conduire ensuite à la production de d’adrénocorticotrophine (ACTH) dans le sang. L’ACTH va stimuler ensuite la synthèse et la libération des hormones glucocorticoïdes, notamment le cortisol, à partir du cortex des glandes surrénales.(50)

L’action des glucocorticoïdes est plus tardive. Ces hormones agissent dans la demi-heure et leurs effets perdurent quelques heures pour une réponse adaptative prolongée permettant la consolidation de la mémoire des informations liées au stresseur. (46)

La réponse physiologique des catécholamines est la suivante :

-augmentation de la fréquence cardiaque

-augmentation de la fréquence respiratoire

-augmentation de la fixation et du transport en oxygène

-augmentation de la glycémie

-augmentation de la quantité d’acides gras sanguins.

Les effets des glucocorticoïdes sont les suivants :

-diminution de la croissance

-affaiblissement du système immunitaire

-diminution de la reproduction

-augmentation de la glycémie

-augmentation de la quantité d’acides gras dans le sang.

Ces effets physiologiques permettent une économie importante de l’énergie (diminution de la croissance, du système immunitaire et de la reproduction) et augmentent la disponibilité des substrats énergétiques.

On observe une redistribution adaptative pour faire face aux situations stressantes.

**Phase de résistance**

Ces modifications biologiques doivent être limitées dans le temps afin de ne pas affecter l’organisme.

**2.4 Les glandes surrénales**

Ces glandes se trouvent au-dessus des reins. Elles sont composées d’une partie corticale et d’une partie médullaire.

**2.4.1.La médullo-surrénale**

Elle est peuplée de cellules chromaffines. Leur fonction est de stocker et de sécréter les catécholamines. Chaque cellule stocke et fabrique un seul type d’hormones : 80 % font de l’adrénaline, 16 % de la noradrénaline et 4 % de la dopamine. Ces cellules sont contrôlées par le système nerveux sympathique.

Les catécholamines ont une très faible demi-vie d’environ une minute, une fois libérées. Elles sont régulées par le foie qui est capable de sécréter une substance inhibitrice de l'adrénaline et/ou de recapturer la noradrénaline.

**2.4.2 La cortico-surrénale**

Ces cellules fabriquent les glucocorticoïdes. Elles ont toutes pour précurseur le cholestérol, ces hormones font donc partie des stéroïdes. Elles circulent dans le sang grâce à des transportines. Le mécanisme du cortisol sera détaillé plus loin.

Ces différentes hormones sont principalement régulées par le foie.

Il existe un rétrocontrôle dans le mécanisme du cortisol, indispensable dans la réponse au stress.


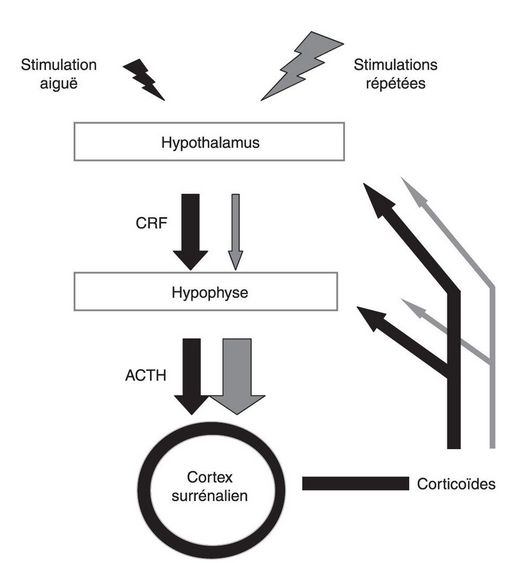


Fig 2 : le mécanisme de rétrocontrôle du cortisol

Nous avons vu qu’il y avait deux catégories d’hormones impliquées dans la réponse au stress, nous allons maintenant étudier plus en détail l’axe corticotrope, puisque les dosages de notre étude sont réalisés à partir du cortisol salivaire.

**2.4.3 L’axe corticotrope et le cortisol**

1. **Physiologie de l’axe corticotrope**

La stimulation de l’axe corticotrope (axe hypothalamo-hypophyso-corticosurrénalien) permet la synthèse par l’hypothalamus d’une hormone, la CRH (corticolibérine), donnant l’ordre à l’hypophyse de sécréter, entre autres, l’ACTH (adrénocorticotropine). Le cortisol, glucocorticoïde secrété par les glandes surrénales, est sous le contrôle de l’ACTH. Le cortisol régule sa sécrétion par des mécanismes de « feed back », essentiels dans l’adaptation de la réponse au stress. (48)

La synthèse du cortisol se produit dans la zone fasciculée du cortex surrénalien et s’effectue à partir du cholestérol endogène et exogène (49). Le cholestérol est transformé en prégnénolone puis en progestérone. La progestérone subit deux hydroxylations successives pour être transformée en corticostérone ou trois hydroxylations successives pour donner de l’hydrocortisone ou cortisol (51). Cette biosynthèse des hormones du cortex surrénal fait intervenir des enzymes tantôt mitochondriales (desmolase et 11béta hydroxylase), tantôt du réticulum endoplasmique (3 béta-deshydrogénase, 17 et 21 hydroxylases).


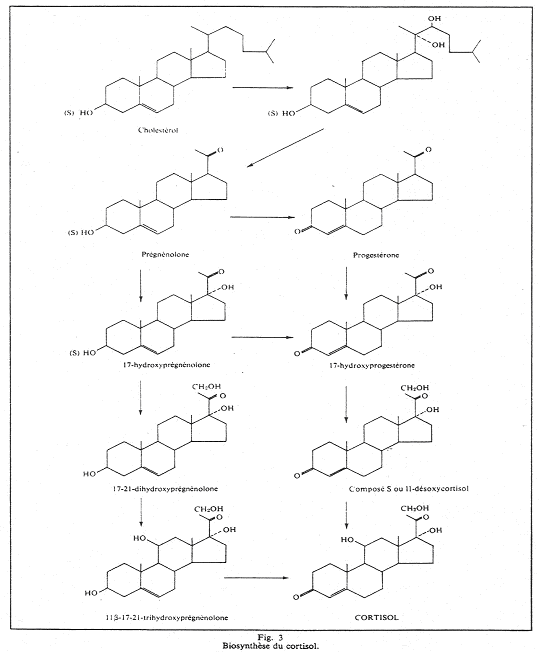


90 % du cortisol plasmatique est lié à la CBG (corticosteroid binding globulin). Cette liaison est réversible et la part active du cortisol est la part non liée.

On peut mesurer la part libre et liée dans le plasma, alors que dans la salive on ne mesure que la part libre.(50, 52, 53, 54, 55, 56, 57, 58).

Cette corrélation constitue un avantage non négligeable pour les études menées sur le terrain (59).

1. **Applications et utilisation du cortisol**

La principale fonction des corticoïdes est d’intervenir dans la régulation des réponses induites par le stress. De nombreuses études ont maintenant démontré que le cortisol, et en particulier le cortisol salivaire, est un marqueur biologique très fiable des niveaux de stress physiologiques et physiques. (54, 57, 58, 59, 60)

La variation spontanée du cortisol est de l’ordre de 15 minutes (61).

Pour une utilisation optimale du cortisol salivaire (62, 63), il faut cependant connaître les variations spontanées ou provoquées au cours des 24 heures. Comme bon nombre de variable biologique, le cortisol fluctue de manière régulière au cours des 24 heures.

La cortisolémie répond à un processus circadien (61). Le rythme du cortisol est le suivant : concentrations plasmatiques très faibles en soirée, suivies d’une augmentation progressives des concentrations situant l’acrophase dans la matinée, avec un pic de cortisol 45 minutes après la phase de réveil ou CAR (cortisol awakening response) (64, 65, 67) puis diminution tout au long de la journée (66, 68).

D’autres facteurs de variation jouent un rôle sur le taux de cortisol comme :

-la station debout (69, 78)

-sexe (70, 74, 75, 84, 87, 88, 89, 90, 91)

-la prise de repas (72, 80)

-la consommation de tabac (73, 76, 84)

-la consommation de café (74, 75)

-la consommation d’alcool (84)

-la lumière (79, 77)

-la prise de médicaments (84, 92, 90)

-le cycle menstruel (70, 92, 90)

-la grossesse et l’allaitement (84)

-l’âge (84, 85)

-la fatigue (86)

-les pathologies psychiatriques (82, 93)

-la pratique du sport (71, 81, 94).

Il apparaît donc évident que le contrôle précis des facteurs environnementaux, comportementaux ou endogènes impliqués dans la régulation de la sécrétion du cortisol, est un pré-requis méthodologique indispensable lors d’études sur le cortisol salivaire. La mesure du taux de cortisol à un moment de la journée ne constitue pas une valeur de référence absolue puisque ce taux peut doubler ou tripler à un autre moment de la journée sous l’influence de composantes circadiennes ou comportementales.

**2.4.4 Du stress chronique à la pathologie**

**A Physiopathologie de la genèse du stress chronique**

Le stress chronique est associé à une production accrue de cortisol. Le premier à travailler sur cette hypothèse fut R.M. Sapolsky (95) qui a étudié la cascade des glucocorticoïdes pour expliquer l’altération des fonctions cérébrales due à une exposition prolongée aux glucocorticoïdes.

La sécrétion prolongée d’hormone glucocorticoïde endommage en particulier les neurones hippocampiques et conduit à une levée de l’inhibition tonique qu’exerce l’hippocampe sur l’activation de l’axe corticotrope.


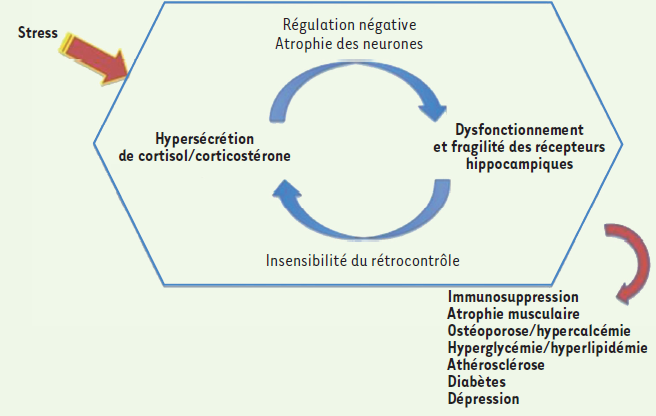


**Figure 3 : Mécanisme des effets délétères du stress chronique**

Cela aboutit à des changements morphologiques dans le cerveau, comme dans le cortex préfrontal, à une altération de l’attention, de la mémoire de travail et de la flexibilité comportementale. Les modifications de l’hippocampe sont associées à des troubles de la mémoire spatiale et de l’apprentissage alors que l’amygdale est associée à une augmentation de la mémoire de peur, à l’anxiété et à l’agressivité. (96, 97)

**B La fonction corticotrope et les pathologies**

L’axe corticotrope apparaît comme un système majeur pour l’élaboration des processus comportementaux et physiologiques adaptatifs. Cependant, la mise en évidence d’effets toxiques provoqués au niveau cérébral par une libération prolongée de glucocorticoïdes (98, 99) augmentée des effets imprévisibles d’une dépression prolongée des défenses immunitaires, conduit à supposer qu’une sécrétion anormalement élevée de glucocorticoïdes pourrait rendre leur fonction non adaptative.

Nous allons maintenant détailler les pathologies liées à des dysfonctionnements de l’axe corticotrope et donc supposées liées au stress.

1. **Sur le plan psychiatrique**

La dépression majeure serait due à une anomalie de la contre-régulation des réponses généralisées au stress résultant en une hypersécrétion de CRH et/ou de catécholamines centrales (100, 101).

Les symptômes majeurs de la dépression mélancolique indiquent que cette pathologie n’est pas associée à un état d’inactivation comme son nom pourrait le laisser supposer mais plutôt à un état d’hypervigilance pathologique (102, 103, 104).

Le stress favoriserait également les troubles du sommeil (111, 112, 113).

1. **Sur le plan cardiovasculaire**

Le stress est un facteur de risque cardiovasculaire secondaire à part entière (107).

Le stress, par le biais de l’hyperactivité de l’axe corticotrope, serait responsable de la mise en place du syndrome métabolique (103, 109, 110). Il jouerait également un rôle dans la formation des calcifications coronaires par le biais du même mécanisme (104,105) et favoriserait la survenue de syndrome coronarien aigu (108). Le cortisol favoriserait également la survenue de l’hypertension (114) par un mécanisme de rétention sodée et d’expansion du volume.

1. **Sur le plan digestif**

On note un important rôle du stress dans les maladies inflammatoires chroniques de l’intestin, le stress altérerait la motricité, la sensibilité, et la fonction sécrétrice du tube digestif (115).

Le stress est connu depuis bien longtemps en réanimation pour être responsable d’ulcères que l’on dit « de stress » (116).

1. **Sur le plan endocrinien**

Des liens ont été mis en évidence entre le stress et une augmentation de la consommation alimentaire (117), ainsi qu’entre stress et diminution de l’activité physique (121).

Le stress pourrait être également un facteur d’infertilité (118).

Il joue un rôle aisément compréhensible dans la perturbation de la fonction glycémique et la genèse du diabète (119). Il peut également perturber la fonction thyroïdienne.

Le stress jouerait également un rôle dans le vieillissement cellulaire (122).

1. **Sur le plan rhumatologique**

Le stress jouerait un rôle dans les poussées de rhumatisme articulaire (123). Le stress peut également avoir un impact sur la fibromyalgie (125, 126).

1. **Sur le plan dermatologique**

Le stress jouerait un rôle dans la genèse de troubles tels que l’eczéma, le prurit, l’urticaire (124).

1. **Sur le plan immunologique**

Le stress, par le biais de l’élévation du cortisol plasmatique, jouerait un rôle dans la diminution des défenses immunitaires et par conséquence directe ou indirecte la formation de pathologies virales ou tumorales (127, 128, 129).

1. **Sur le plan professionnel :**

Dans le monde du travail et en particulier celui des soins, le stress chronique peut conduire au syndrome d’épuisement professionnel ou burn-out (130, 131, 132) : il s’agit d’un état d’épuisement émotionnel, physique et psychique.

**2.5 Population étudiée dans notre étude**

La présente étude aura pour but d’étudier le stress en régulation.

La population étudiée sera constituée du personnel du centre 15 du CHR Metz Thionville (Hôpital de Mercy) : ARM, médecins urgentistes et généralistes répondant aux critères d'éligibilité de l’étude.

**2.5.1 Mission générale des ARM :**

Les ARM sont des agents hospitaliers qui exercent un rôle propre dans la gestion des appels d'aide médicale urgente et de permanence des soins qui parviennent au centre de régulation des appels du SAMU-centre 15.

Ils exercent l'ensemble des missions qui leurs sont attribuées dans le cadre législatif et réglementaire, sous le contrôle opérationnel du ou des médecins régulateurs, hospitaliers et généralistes. Les ARM leur rendent compte en permanence de leurs actions.
Ils entretiennent le matériel en salle de régulation et tiennent à jour la documentation du SAMU centre 15 : patients répertoriés, plan de secours, listes de garde, annuaires opérationnels...

**2.5.2 Compétences générales des ARM**

- Connaître l'organisation et les missions de l'aide médicale urgente
- réceptionner les appels
- trier, par un interrogatoire initial succinct mais précis, les appels non médicaux et les traiter en les hiérarchisant selon leur degré d'urgence
- déceler une urgence vitale
- gérer la communication et l'adapter à la réaction et au stress des appelants
- transmettre les informations pertinentes aux médecins régulateurs
- appliquer les décisions des médecins régulateurs et les procédures réflexes
- connaître l'ensemble des procédures et des documents opérationnels d'aide à la régulation en situation quotidienne ou de crise
- connaître les ressources en moyen pré-hospitaliers et hospitaliers du département, de la région et de la zone de défense
- connaître les critères de gravité des grandes pathologies d'urgence et les gestes et soins d'urgence
- maîtriser les divers outils de l'information, des communications et de l'informatique
- renseigner les documents opérationnels et administratifs liés aux affaires traitées et à la maintenance opérationnelle du centre de régulation

**2.5.3 Rôle des médecins régulateurs**

**Rôle du médecin urgentiste :**

- régulation identifiées P0 et P1
- bilans identifiés B
- demande de transfert inter-hospitalier
- demande des autres SAMU
- demande de SMUR
- interventions sur la voie publique
- bilan SMUR

**Rôle du médecin généraliste ou hospitalier non urgentistes**

- régulations identifiées P2
- demande de conseils médicaux
- appels relevant de la permanence des soins ambulatoires

**3. Objectifs de la recherche :**

**3.1. Objectif Principal :**

Décrire le stress biologique à travers le dosage de la cortisolémie ainsi que le stress perçu par une évaluation visuelle analogique de stress chez les ARM et les médecins lors du traitement d'un appel au centre 15.

|  |
| --- |
|  |

**3.2. Objectifs secondaires :**

- Comparer le stress perçu et biologique chez les ARM en fonction des différents postes

- Etudier la pénibilité des différents postes chez les ARM

- Etudier la corrélation entre stress perçu et stress biologique

- Etudier la corrélation entre gravité de la situation téléphonique et stress

**4. Conception de la recherche**

Etude physiologique contrôlée chez l’homme sain, chaque sujet est son propre témoin.

Quatre conditions expérimentales chez les ARM et deux conditions expérimentales chez les médecins urgentistes et libéraux.

**4.1. Enoncé précis du** **critère d’évaluation principal ET, le cas échéant, des critères d’évaluation** **secondaires**

- Critère d’évaluation principal

**Mesure objective du stress : dosage du cortisol salivaire dans différentes conditions expérimentales.**

4 conditions expérimentales pour les ARM : « appel, effecteur, bilan, témoin »

2 conditions expérimentales pour les médecins : « appel, témoin ».

- Critères d’évaluation secondaires

**Mesures subjectives du stress**

**- EVA : évaluation du stress aigu**

L’évaluation du stress aigu par EVA sera réalisée de façon concomitante à chaque série de prélèvements de cortisol salivaire. Il sera demandé à chacun des sujets de coter son stress sur une échelle de 0 à 10. L’EVA a été validée dans les études sur le stress. (133, 134, 135, 136, 137)

**- Questionnaire SPPN : Stress Professionnel Positif et Négatif : évaluation du stress chronique**

Le questionnaire de stress chronique SPPN sera complété une seule fois par chaque sujet. Il sera distribué au centre 15, le matin à 10 heures (au moment de la prise de fonction) chez l’ensemble des sujets en activité ce jour là et avant de commencer les prélèvements de cortisol salivaire (138, 139, 140).

**- Test de personnalité NEO PI R : (NEO personality inventory revised) (141) : évaluation des composantes de la personnalité.**

Le test de personnalité sera complété une seule fois par chaque sujet. Il sera distribué lors du temps de travail, le sujet bénéficiera d’un temps libre pour répondre à l’étude.

Le sujet répond à un questionnaire de 180 questions sous la supervision du psychologue du travail du CHR de Mercy. Les questionnaires seront ensuite analysés par ce même psychologue.

## 4.2. Description de la méthodologie de la recherche, accompagnée de sa présentation schématique précisant notamment les visites et les examens prévus.

- **Visite de pré inclusion**

La visite de pré-inclusion aura lieu entre *30 jours* et au plus tard *15 jours* avant la visite d’inclusion.

Une information sur le déroulement et les objectifs de l’étude sera effectuée par l’investigateur.

Tous les PARM et les médecins participeront à cette réunion.

- **Visite d’inclusion**

Un questionnaire contenant les critères d’éligibilité de l’étude sera distribué à chaque ARM et à chaque médecin du service, ainsi qu’une note d’information sur l’étude et un formulaire de consentement éclairé.

Suite à cette visite, seuls les sujets éligibles ayant signé un consentement éclairé pourront participer à l’étude.

- Visites de suivi

**Lors de ces visites, les dosages de cortisol salivaire seront réalisés et les questionnaires (EVA et SPPN) seront complétés.**

**4.2.1 Organisation du centre 15 au CHR METZ-THIONVILLE et conditions expérimentales évaluées**

Les ARM travaillent en poste de 12 heures avec des roulements de deux jours, une nuit, puis trois jours de repos pour un total de 36 heures par semaine.

Les médecins urgentistes travaillent également en poste de 12 heures, pour une durée totale de 48 heures par semaine, en faisant des rotations en service de médecine, régulation et poste SMUR/déchoquage.

Les médecins libéraux travaillent le plus souvent en poste de 12 heures.

Le fonctionnement du centre 15 de Metz est le suivant : les assistants de régulation ou ARM se répartissent en trois postes :

- l'**ARM appel** est en première ligne. C’est lui qui reçoit les appels provenant de l’extérieur. Il évalue la gravité de la situation et, selon la gravité du cas, transmet l’appel au médecin urgentiste ou au médecin généraliste. C’est le poste ressenti le plus stressant.

- l'**ARM effecteur** déploie les différents moyens d’intervention. C’est lui qui organise les transports et qui est chargé d’appeler les ambulances, les pompiers ou les équipes de SMUR selon la gravité de l’intervention. Il intervient après avoir reçu les consignes soit du médecin généraliste, soit du médecin urgentiste.

- l'**ARM bilan**  est chargé de recueillir le bilan des différentes interventions.

Le fonctionnement des médecins est différent. L'ARM appel transmet au médecin libéral (le plus souvent un médecin généraliste) les appels peu graves nécessitant soit un conseil médical, soit une simple ambulance. Le médecin urgentiste gère quant à lui les situations plus graves nécessitant une prise en charge plus lourde, c’est le seul qui peut déclencher un départ du SMUR.

Cette étude sera réalisée en incluant le même nombre d'ARM expérimentés (plus de cinq ans d’expérience) que d'ARM jeunes (moins de cinq ans d’expérience). On étudiera également le même assistant de régulation sur les trois postes.

Lors de notre étude, un appel sera étudié. C’est cet appel qui servira de référence. Une série de prélèvements de cortisol salivaire sera débutée chez l'ARM appel en poste à ce moment. Un premier prélèvement de cortisol salivaire sera effectué lorsque l'ARM aura raccroché le téléphone. Ce premier prélèvement sera considéré comme le prélèvement T0. Les autres prélèvements seront ensuite réalisés toutes les 15 minutes pendant une heure et demie, puis un dernier prélèvement sera effectué à deux heures de T0. Dès que l'appel est transmis au médecin, que ce soit le médecin urgentiste ou le médecin smuriste, les prélèvements débutent pour le médecin. T0 est le moment où le médecin raccroche et commence à mâcher le coton. Puis le même appel est étudié de la même manière par l'ARM effecteur puis par l'ARM bilan.

Tous les appels seront étudiés de cette manière.

**4.2.2 Organisation pratique pour l’étude**

.

***DOSAGES DU CORTISOL SALIVAIRE***

***Matériel***

Ce dosage sera réalisé à l’aide de salivettes  Biomnis  ou Sarstedt et sera couplé aux mesures subjectives du stress par EVA.

Les salivettes seront fournies par le laboratoire Biomnis ou le laboratoie Sarsetedt. Elles sont constituées d’un coton que l’on doit imbiber de salive, à maintenir dans la bouche et mâcher deux à trois minutes (cf annexes). Les prélèvements seront ensuite anonymisés à l’aide de numéros, dans un souci de confidentialité. Les salivettes seront ensuite conservées au congélateur à -4 degrés dans les congélateurs du laboratoire de l’hôpital de Mercy pour ensuite être analysées sur place. Les échantillons peuvent se conserver un an à une température de - 4 degrés.

***Conditions expérimentales***

Le cortisol salivaire sera recueilli chez chaque ARM, pendant 3 jours de travail, sur trois postes différents : appel, déploiement de moyens ou effecteur, et bilan.

Les participants seront informés des bonnes conditions de prélèvements du cortisol salivaire. Ils s'engagent une fois intégrés dans l'étude à ne pas consommer de boisson ou d'aliments dans la demi-heure précédant le recueil de salive et également durant les deux heures de prélèvements. Les sujets auront aussi comme consigne de ne pas fumer trente minutes avant les prélèvements et pendant toute la durée des prélèvements. En ce qui concerne les autres facteurs de variation du cortisol salivaire, la luminosité ne varie pas au cours de la journée, les sujets travaillent en position assise, la station debout ne sera pas prise en compte.

Les appels pendant lesquels les dosages auront lieu seront des appels intervenant tous sur la même plage horaire afin d’éviter les biais dus à la variation circadienne inter et intra individuelle de cortisol salivaire.

Le premier appel survenant chez l’ARM appel sera notre appel de référence. Cependant les appels par erreur seront exclus de l'étude. Lors de l’appel, un dosage de cortisol salivaire sera réalisé chez l’ARM appel. Le premier dosage sera noté T0. Un autre dosage de cortisol salivaire sera réalisé chez l’ARM appel toutes les 15 minutes à partir de T0 et ce pendant 1h30, puis un dernier dosage sera effectué 2h après l’appel, soit huit dosages en tout pour réaliser une courbe de stress aigu. Les dosages de cortisol seront tous réalisés à la même heure pour éviter les variations du nycthémère. Le début de chaque prélèvement débutera après que le sujet a raccroché le téléphone.

Le même dosage de cortisol salivaire suivant le même principe sera ensuite réalisé chez le médecin à qui est transmis l’appel. Puis le même dosage sera réalisé chez l'ARM effecteur puis l'ARM bilan. Le jour suivant, le dosage de cortisol salivaire sera réalisé chez les mêmes ARM à des postes différents.

Trois assistants de régulation médicale effectueront donc les dosages de cortisol salivaire chaque jour, ainsi qu'un médecin, soit urgentiste, soit libéral selon la gravité de l'appel.

Chaque appel intercurrent sera noté durant les mesures de cortisol salivaire. Chaque événement, durant les deux heures de prélèvement, sera également répertorié afin d'éviter les biais et de permettre une meilleure analyse des courbes obtenues.

Un dosage de cortisol salivaire sera également réalisé lors d’une journée de repos (le lendemain d’une nuit de travail n’est pas considéré comme un jour de repos) pour obtenir une valeur de référence et permettre de limiter les variations intra individuelles. Ce dosage suivra une méthodologie différente puisque le but n'est plus d'étudier un événement aigu mais d'avoir une courbe de référence. Huit prélèvements seront réalisés à intervalle régulier pendant la journée. Un premier prélèvement sera réalisé au réveil et sera considéré comme T0, puis il y aura un prélèvement toutes les deux heures. Les échantillons seront conservés à l’air ambiant par le sujet puis ramenés le lendemain au CHR. Une fiche explicative sera remise à chaque participant.

Les échantillons de salive seront anonymisés. Chaque sujet portera un numéro associé au temps de prélèvement pour chaque échantillon.

Les échantillons seront conservés à -4 degrés dans un congélateur dédié, prêté gracieusement par le laboratoire de l’hôpital de Mercy. Ils seront ensuite analysés au laboratoire du CHR de Mercy.

**4.2.3 Remplissage des questionnaires d’évaluation du stress**

* SPPN (stress chronique) : il sera complété une seule fois par chaque sujet, au centre 15, le matin à 10 heures (au moment de la prise de fonction) et avant de commencer les prélèvements de cortisol salivaire.

* EVA (stress aigu) : cotation réalisée de façon concomitante à chaque dosage de cortisol. Chaque sujet devra coter son stress sur une échelle de 1 à 10.

* NEO PI R (test de personnalité) : il sera complété une seule fois par chaque sujet, au centre 15, sous la surveillance du psychologue du travail du CHR de Mercy.

**4.2.4 Autres données recueillies**

Lors des périodes de réalisation des dosages de cortisol salivaire (soit pendant 2h par sujet), les événements intercurrents seront répertoriés (autres appels, contextes particuliers, …)

Par ailleurs, lors de chaque appel, la gravité de celui-ci sera évaluée selon la classification suivante pour rechercher une corrélation entre niveau de stress physiologique et gravité de l’appel.

Pour les ARM :

Ils appliquent trois niveaux de priorité dans la transmission au médecin régulateur :

P0 : déclenchement réflexe d'une équipe SMUR, suivi d'une régulation prioritaire

P1 : régulation prioritaire

P2 : régulation qui peut être mise en attente, sans risque pour le patient, d'autres régulations étant en cours

Pour les médecins régulateurs :

Les médecins régulateurs assument des décisions réparties en quatre niveaux d'urgence

R1 : urgence vitale patente ou latente imposant l'envoi d'un moyen de réanimation (SMUR)

R2 : urgence vraie sans urgence vitale nécessitant l'envoi d'un médecin de proximité, d'une ambulance ou d'un VSAV dans un délai contractualisé entre le régulateur, l'effecteur et l'appelant.

R3 : recours à la permanence des soins, le délai ne constituant pas un risque en soi ; une prescription médicamenteuse d'attente peut être proposée

R4 : conseil médical ou prescription médicamenteuse par téléphone

Pour les ARM bilan :

Les ARM appliquent trois niveaux de priorité dans la transmission d'un bilan au médecin régulateur :

B0 : bilan avec des signes de détresse qui n'apparaissent pas lors de l'appel. Cet appel doit être pris en priorité par le médecin régulateur urgentiste

B1 : bilan apportant des informations complémentaires non présentes lors de la régulation initiale mais sans signe de détresse ou de gravité

B2 : bilan conforme aux données recueillies lors de la régulation

## 4.3. Description des mesures prises pour réduire et éviter les biais incluant notamment le tirage au sort et les méthodes de mise en insu

Cette étude est réalisée en ouvert et ne nécessite pas de tirage au sort.

Chaque sujet sera son propre témoin. Les sujets seront évalués dans des conditions expérimentales identiques et propres à chaque corps de métier (ARM ou personnel médical).

Cf point 4.2.

## 4.4. Durée prévue de participation des personnes

Durée de la période d’inclusion : 1 mois

Durée de participation de chaque sujet : quatre fois deux heures pour les ARM et deux fois deux heures pour les médecins.

ARM : 4 fois 8 prélèvements / Médecins : 2 fois 8 prélèvements

Durée totale de l’étude: 6 mois

**4.5. Identification de toutes les données à recueillir directement dans les cahiers d'observation, qui seront considérées comme des données source.**

Non applicable

**5. Sélection et exclusion des personnes de la recherche :**

**5.1. Critères d'inclusion**

- Personnel médical et paramédical du centre 15 (assistants de régulation médicale, médecins urgentistes, médecins libéraux)
- Ayant reçu une information sur l’étude
- Ayant signé un consentement de participation à l’étude
- Affiliation obligatoire à un régime de sécurité sociale

**5.2. Critères de non-inclusion**

- Ethylisme chronique
- Les femmes sous traitement oestroprogestatifs
- Femmes enceintes
- Les sportifs de haut niveau
- Les personnes sous traitement médicamenteux à base de stéroïdes
- Les personnes suivies pour désordres psychiatriques (en particulier la dépression)
- Fièvre le jour du prélèvement
- Participants volontaires porteurs de pathologies endocriniennes

**5.3. Procédure d'arrêt prématuré de la recherche ou d'exclusion (ARRÊT DU SUIVI)**

**Critères et modalités d’arrêt du suivi**

- *d'arrêt définitif*

A la demande du sujet

- *d'arrêt temporaire*

Non applicable

**5.4. Modalités de recrutement**

Le personnel médical et paramédical du centre 15 du CHR METZ-THIONVILLE (permanenciers, médecins urgentistes, médecins libéraux) sera informé de la réalisation de cette étude. Chaque personnel sera sollicité pour participer à cette étude dans la mesure où il remplit les critères d’éligibilité.

*S’il y a plus de 20 volontaires pour participer à notre étude, un tirage au sort sera réalisé pour désigner les participants.*

**6. DESCRIPTION DE L’EXPOSITION**

*Voir point 4.2*

**7. Evaluation de la sécurité :**

**7.1. Description des paramètres d’évaluation de la sécurité**

Les seules contraintes pour les sujets participants à cette étude sont les dosages de cortisol salivaire qui sont effectués via une salivette, à savoir un petit coton à mâcher par les sujets afin de l’imbiber de salive (cf. Annexe 1).

Dans le cadre de cette étude, les seuls risques envisageables sont ceux liés à l’utilisation des salivettes, à savoir une éventuelle intolérance au coton utilisé ou un mésusage de la salivette.

**7.2. Méthodes et calendrier prévus pour mesurer, recueillir et analyser les paramètres d’évaluation de la sécurité**

Les sujets participants à l’étude seront interrogés sur les risques cités au point 7.1

**7.3 Procédures mises en place en vue de l’enregistrement et de la notification des évènements indésirables**

**7.3.1 Définitions**

Un **événement indésirable** est une manifestation nocive survenant chez une personne qui se prête à une recherche biomédicale, que cette manifestation soit liée ou non à la recherche ou au produit sur lequel porte cette recherche.

Un **effet indésirable** est une réaction nocive et non voulue liée à un médicament expérimental, se produisant à la posologie normalement utilisée chez l’homme.

**Un évènement indésirable grave ou effet indésirable grave** **(EIG)** est un évènement ou un effet ayant pu contribuer au décès du patient, à la mise en jeu de son pronostic vital, à son hospitalisation ou à la prolongation de son hospitalisation, à une incapacité ou à un handicap important ou durable, ou se traduire par une anomalie ou une malformation congénitale

**La liste des effets indésirables attendus** liés à l’étude est établie à partir du document de référence.

Un EIG est inattendu (EIGI) lorsqu’il ne figure pas sur cette liste.

**Un fait nouveau** peut être : fréquence inattendue d’un EIG attendu, événement indésirable grave lié à la procédure de l’essai, efficacité insuffisante dans les maladies à pronostic vital, données non cliniques.

**7.3.2 Document de référence permettant de définir le caractère attendu d’un EIG**

*Non applicable dans le cadre de cette étude*

**7.3.3 Liste des effets indésirables attendus**

*Non applicable dans le cadre de cette étude*

**7.3.4 Transmission des EIG et faits nouveaux**

Dès qu’un investigateur prend connaissance d’un EIG ou d’un fait nouveau, il le déclare sans délai au promoteur en faxant la fiche de déclaration d’EIG.

- S’il s’agit d’un effet indésirable grave inattendu (EIGI) ou si c’est un fait nouveau, le promoteur entre en contact avec l’investigateur pour rédiger un rapport initial qui sera transmis à l’ANSM, au CPP, à l’investigateur principal dans les 7 jours en cas de décès ou de mise en jeu du pronostic vital, sinon dans les 15 jours.

Lorsque l’évènement n’est pas résolu à la date d’envoi du fax, l’investigateur est tenu d’envoyer un rapport complémentaire afin de documenter l’évolution ou de réactualiser les données manquantes.

- S’il s’agit d’un effet indésirable grave attendu, il sera colligé par le promoteur en vue de la rédaction des rapports annuels de sécurité.

**7.3.5 Transmission des effets indésirables non graves**

Ils seront décrits succinctement par l’investigateur sur la fiche récapitulative dédiée à cet effet dans le cahier d’observation.

**7.4. Modalités et durée de suivi des personnes suite à la survenue d’évènements indésirables**

Lorsque un évènement indésirable grave persiste, y compris après la fin de l’étude, l’investigateur suivra le patient jusqu’à ce que l’évènement soit considéré comme résolu et transmettra les données de suivi au promoteur**.**

**7.5. Comités spécifiques de la recherche (variable selon les protocoles)**

**Comité de pilotage**

Il sera constitué des initiateurs cliniciens du projet, du biostatisticien en charge du projet, des représentants du promoteur.

Il définira l'organisation générale et le déroulement de la recherche et coordonnera les informations.

Il déterminera initialement la méthodologie et décidera en cours de recherche des conduites à tenir dans les cas imprévus, surveillera le déroulement de la recherche en particulier sur le plan de la tolérance et des évènements indésirables.

**7.6 Rapports de sécurité**

- Rapports annuels de sécurité : le promoteur rédige les rapports annuels de sécurité et les transmet à l’ANSM, au CPP et à l’investigateur principal. L’investigateur principal transmettra au promoteur toutes les données nécessaires à la rédaction de ce rapport.
- Rapport final : il est rédigé par le promoteur et l’investigateur principal dans un délai d’un an après la fin de l’étude. Tous les investigateurs sont informés des résultats de l’étude. Un résumé est adressé à l’ANSM par le promoteur.

**8. Statistiques**

**8.1. description des méthodes statistiques prévues, y compris du calendrier**

**des analyses intermédiaires prévues**

**Description de l’échantillon** : les variables qualitatives seront décrites en pourcentage, effectif et intervalle de confiance à 95%, les variables quantitatives seront décrites en moyennes et écarts-types ou médiane, étendue et intervalle interquartiles.

**Description du stress biologique** : il sera décrit en calculant la différence entre le pic ou les pics de cortisol observés et le taux de cortisol mesuré au repos (moyennes et écarts-types ou médiane, étendue et intervalle interquartiles).

**Description du stress perçu** : le score de l’EVA sera décrit en termes de moyenne et écart type.

**A titre exploratoire** : comparaison du delta pic/taux de base de cortisol selon les 3 postes des ARM avec un test non paramétrique de Kruskal Wallis, de même selon l’ancienneté des ARM, la gravité de l’appel et le statut du médecin (urgentiste ou libéral) ; comparaison du delta pic/taux de base de cortisol selon le stress chronique (questionnaire SPPN) et la personnalité (questionnaire NEO PI-R).

Le score à l’EVA (stress perçu) sera comparé au delta pic/taux de base de cortisol (stress biologique) à l’aide du coefficient de corrélation de Spearmann.

**8.2. nombre prévu de personnes à inclure dans la recherche, avec sa justification statistique**

Il s’agit d’une étude dont l’objectif principal est descriptif, et qui peut servir d’étude de faisabilité concernant les objectifs comparatifs (secondaires). Il est prévu d’inclure 20 ARM et 20 médecins (urgentiste ou libéral).

**8.3. degré de signification statistique prévu**

Etude descriptive. Les tests statistiques seront réalisés à visée exploratoire, avec un risque alpha fixé à 5%

- 1. **critères statistiques d'arrêt de la recherche**

Non Applicable.

**8.5. méthode de prise en compte des données manquantes, inutilisées ou non valides**

Pas d’extrapolation des données manquantes. Les sujets pour lesquels plus de la moitié des données sont manquantes seront exclus des analyses.

**9. Droit d'accès aux données et documents source**

Toutes les données et informations concernant le participant volontaire resteront strictement confidentielles. Les personnes ayant un accès direct conformément aux dispositions législatives et réglementaires en vigueur, notamment les articles L.1121-3 et R.5121-13 du code de la santé publique (par exemple, les investigateurs, les personnes chargées du contrôle de qualité, les moniteurs, les assistants de recherche clinique, les auditeurs et toutes personnes appelées à collaborer aux essais) prennent toutes les précautions nécessaires en vue d'assurer la confidentialité des informations relatives à l’étude et aux personnes qui s'y prêtent et notamment en ce qui concerne leur identité ainsi qu’aux résultats obtenus. Les données collectées par ces personnes au cours des contrôles de qualité ou des audits sont alors rendues anonymes.

Les sujets seront identifiés par un numéro attribué chronologiquement selon l'ordre d'inclusion dans l'étude. Les données seront recueillies de sorte à empêcher l'identification indirecte des participants : sexe, expérience dans le service (plus ou moins de 5 ans), âge en classes de 10 ans.

**10. Contrôle et assurance de la qualité**

**10.1 Monitoring**

Les ARC représentants du promoteur effectueront des visites du centre investigateur au rythme correspondant au schéma de suivi des sujets dans le protocole :

- Visite d’ouverture du centre : avant inclusion, pour une mise en place du protocole et prise de connaissance avec les différents intervenants de la recherche biomédicale.

- Lors des visites suivantes, les cahiers d'observation seront revus au fur et à mesure de l'état d'avancement de la recherche par les ARC. L’ensemble des investigateurs qui incluent les sujets dans l’étude ou qui assurent leur suivi s’engagent à recevoir les ARC à intervalles réguliers.

Lors de ces visites sur site et en accord avec les Bonnes Pratiques Cliniques, les éléments suivants seront revus :

- Respect du protocole et des procédures définies pour la recherche,
- Vérification des consentements éclairés des sujets
- Examen des documents source et confrontation avec les données reportées dans le cahier d’observation quant à l’exactitude, les données manquantes, et la cohérence des données.

- Visite de fermeture : dernière mise en conformité réglementaire le cas échéant et préparation à l’archivage des documents de l’étude.

A ce titre, l’investigateur s’engage à mettre à la disposition de l’ARC lors de ses visites de monitoring :

⮚ Les dossiers source des sujets (questionnaires, dosages salivaires, …)

⮚ Les cahiers de recueil de données (cahiers d’observation)

⮚ Les formulaires de consentement des sujets inclus

**10.2 Transcription des données dans le cahier d’observation**

Toutes les informations requises par le protocole doivent être fournies dans le cahier d’observation et une explication donnée par l’investigateur pour chaque donnée manquante.

Les données devront être transférées dans les cahiers d'observation au fur et à mesure qu'elles sont obtenues qu'il s'agisse de données cliniques ou para-cliniques. Les données devront être copiées de façon nette et lisible à l'encre noire dans ces cahiers (ceci afin de faciliter la duplication et la saisie informatique).

Les données erronées dépistées sur les cahiers d'observation seront clairement barrées et les nouvelles données seront copiées sur le cahier avec les initiales et la date par le membre de l'équipe de l'investigateur qui aura fait la correction.

L'anonymat des sujets sera assuré par un numéro de code et les initiales de la personne qui se prête à la recherche sur tous les documents nécessaires à la recherche, ou par effacement par les moyens appropriés des données nominatives sur les copies des documents source, destinés à la documentation de la recherche.

Les données informatisées sur un fichier seront déclarées à la CNIL selon la procédure adaptée au cas (voir point 11.4).

**11. Considérations éthiques ET REGLEMENTAIRES**

Le promoteur et les investigateurs s’engagent à ce que cette recherche soit réalisée en conformité avec la loi n°2004-806 du 9 août 2004, ainsi qu’en accord avec les Bonnes Pratiques Cliniques (I.C.H. version 4 du 1^er^ mai 1996 et décision du 24 novembre 2006) et la déclaration d’Helsinki (Principes éthiques applicables aux recherches médicales sur des sujets humains, Séoul 2008).

La recherche est conduite conformément au présent protocole. Les investigateurs s’engagent à respecter le protocole en tous points en particulier en ce qui concerne le recueil du consentement et la notification et le suivi des événements indésirables graves.

**11.1 Demande d’autorisation auprès de l’ANSM**

Pour pouvoir démarrer la recherche, le promoteur doit soumettre un dossier de demande d’autorisation auprès de l'autorité compétente l’ANSM. L'autorité compétente, définie à l'article L. 1123-12, se prononce au regard de la sécurité des personnes qui se prêtent à une recherche biomédicale, en considérant notamment la sécurité et la qualité des produits utilisés au cours de la recherche conformément, le cas échéant, aux référentiels en vigueur, leur condition d'utilisation et la sécurité des personnes au regard des actes pratiqués et des méthodes utilisées ainsi que les modalités prévues pour le suivi des personnes.

**11.2 Demande d’avis au Comité de Protection des Personnes**

En accord avec l'article L.1123-6 du Code de Santé Publique, le protocole de recherche doit être soumis par le promoteur à un Comité de Protection des Personnes. L'avis de ce comité est notifié à l’autorité compétente par le promoteur avant le démarrage de la recherche.

**11.3 Modifications**

Les modifications apportées au protocole de l’étude devront être qualifiées en substantielles ou non.

Une modification substantielle est une modification susceptible, d'une manière ou d'une autre, de modifier les garanties apportées aux personnes qui se prêtent à la recherche biomédicale (modification d’un critère d’inclusion, prolongation d’une durée d’inclusion, participation de nouveaux centres,…).

Après le commencement de la recherche, toute modification substantielle de celle-ci à l’initiative du promoteur doit obtenir, préalablement à sa mise en œuvre, un avis favorable du comité de protection des personnes et une autorisation de l’autorité compétente. Dans ce cas, si cela est nécessaire, le comité s’assure qu’un nouveau consentement des personnes participant à la recherche est bien recueilli.

Toute modification substantielle devra faire l’objet par le promoteur d’une demande d’autorisation auprès de l’ANSM et/ou d’une demande d’avis du CPP.

**11.4 Déclaration CNIL**

Cette recherche entre dans le cadre de la « Méthodologie de référence » (MR-001) en application des dispositions de l’article 54 alinéa 5 de la loi du 6 janvier 1978 modifiée relative à l’informatique, aux fichiers et aux libertés. Ce changement a été homologué par décision du 5 janvier 2006. Le CHR de Metz-Thionville a signé un engagement de conformité à cette « Méthodologie de référence ».

Seules les données nécessaires à la recherche seront recueillies. Le sujet a néanmoins le droit de s’opposer à ce que les données le concernant fassent l’objet d’un traitement automatisé. Le sujet aura à tout moment le droit d’accéder aux données le concernant. Il aura également le droit de demander à ce que les données inexactes ou devenues inexactes soient rectifiées. Il pourra à tout moment exercer ses droits auprès du Dr Michel AUSSEDAT investigateur principal.

Pour toutes les informations de nature médicale, ses droits pourront être exercés directement ou par l’intermédiaire du médecin de son choix.

**11.5 Note d’information et Consentement éclairé**

Avant de recueillir le consentement du sujet, l’investigateur s’engage lui à délivrer une information claire et la plus complète possible sur l'étude envisagée ; il lui remet également la notice d’information.

Le formulaire de consentement sera signé en deux exemplaires par le sujet et le médecin investigateur :

⮚ Un exemplaire sera remis à la personne participant à la recherche

⮚ Un exemplaire sera conservé et archivé par l'investigateur

Les modalités d’information et de recueil du consentement dans le cadre de cette étude sont décrites au point 4.2

**11.6 Rapport final de la recherche**

Le rapport final de la recherche sera écrit en collaboration par l’investigateur principal et le biostatisticien pour cette recherche. Ce rapport sera soumis à chacun des investigateurs pour avis. Une fois qu'un consensus aura été obtenu, la version finale devra être avalisée par la signature de chacun des investigateurs et adressée au promoteur dans les meilleurs délais après la fin effective de la recherche. Un rapport rédigé selon le plan de référence de l’autorité compétente doit être transmis à l’autorité compétente ainsi qu’au CPP dans un délai de un an, après la fin de la recherche, s’entendant comme la dernière visite de suivi du dernier sujet inclus. Ce délai est rapporté à 90 jours en cas d’arrêt prématuré de la recherche.

**12. TRAITEMENT DES DONNEES ET CONSERVATION DES DOCUMENTS ET DES DONNEES RELATIVES A LA RECHERCHE**

Les documents d’une recherche entrant dans le cadre de la loi sur les recherches biomédicales doivent être archivés par toutes les parties pendant une durée de 15 ans après la fin de la recherche*.*

*(voir BPC, chapitre 8 : documents essentiels)*

cet archivage indexé comporte :

- Les copies de courrier d’autorisation de l’ANSM et de l’avis obligatoire du CPP
- Les versions successives du protocole (identifiées par le n° de version et la date de version),
- Les courriers de correspondance avec le promoteur,
- Les consentements signés des sujets sous pli cacheté avec la liste ou registre d’inclusion en correspondance,
- Le cahier d’observation complété et validé de chaque sujet inclus,
- Toutes les annexes spécifiques à l’étude,
- Le rapport final de l’étude provenant de l’analyse statistique et du contrôle qualité de l’étude (double transmis au promoteur).
- Les certificats d’audit éventuels réalisés au cours de la recherche

La base de données ayant donné lieu à l’analyse statistique doit aussi faire l’objet d’archivage par le responsable de l’analyse (support papier ou informatique).

**13. Financement et assurance**

**13.1 Assurance**

Le promoteur a souscrit auprès de la SHAM pour toute la durée de l'étude une assurance garantissant sa propre responsabilité civile ainsi que celle de tout intervenant impliqué dans la réalisation de l'étude, indépendamment de la nature des liens existant entre les intervenants et le promoteur.

### 13. 2 financement

Cette étude est soumise à différents appels à projets afin de couvrir les surcoûts spécifiques engendrés par sa mise en œuvre.

**14. Règles relatives à la publication.**

Le CHR de Metz-Thionville est propriétaire des données et aucune utilisation ou transmission à un tiers ne peut être effectuée sans son accord préalable.

Le CHR de Metz-Thionville doit être mentionné comme étant le promoteur de la recherche biomédicale et comme soutien financier le cas échéant.

Les publications incluront dans la liste des auteurs *Sarah Bedini, Laurence Weibel, Michel Aussedat selon leur implication dans la rédaction des articles.*

**15. Liste des annexes.**

*Annexe 1 : notices d'utilisation de la salivette*

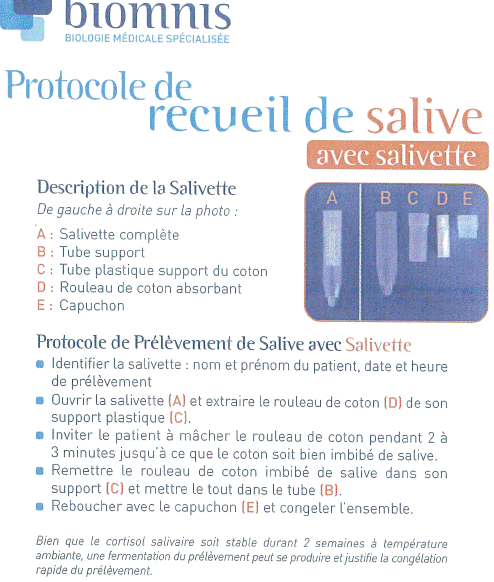


Annexe 2 : Echelle EVA


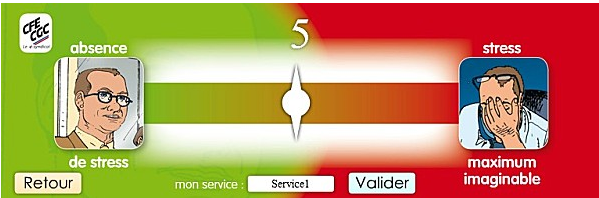


Annexe 3 : questionnaire SPPN


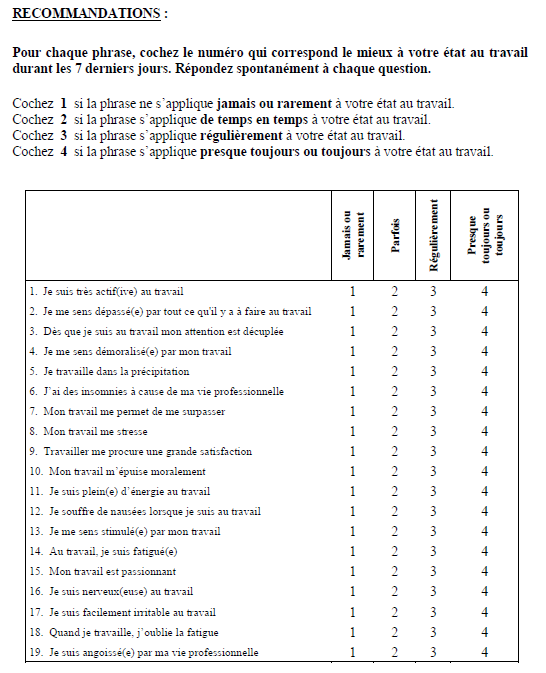


**16. REFERENCES BIBLIOGRAPHIQUES**

# Weibel L, Gabrion I, Aussedat M, Kreutz G. [Work-related stress in an emergency medical dispatch center.](http://www.ncbi.nlm.nih.gov/pubmed/12658250) Ann Emerg Med. 2003 Apr;41(4):500-6.

# [Bhui KS](http://www.ncbi.nlm.nih.gov/pubmed?term=Bhui%20KS%5BAuthor%5D&cauthor=true&cauthor_uid=22496705), [Dinos S](http://www.ncbi.nlm.nih.gov/pubmed?term=Dinos%20S%5BAuthor%5D&cauthor=true&cauthor_uid=22496705), [Stansfeld SA](http://www.ncbi.nlm.nih.gov/pubmed?term=Stansfeld%20SA%5BAuthor%5D&cauthor=true&cauthor_uid=22496705), [White PD](http://www.ncbi.nlm.nih.gov/pubmed?term=White%20PD%5BAuthor%5D&cauthor=true&cauthor_uid=22496705). A synthesis of the evidence for managing stress at work: a review of the reviews reporting on anxiety, depression, and absenteeism. [J Environ Public Health.](http://www.ncbi.nlm.nih.gov/pubmed/?term=A+synthesis+of+the+evidence+for+managing+stress+at+work%3A+a+review+of+the+reviews+reporting+on+anxiety%2C+depression%2C+and+absenteeism.) 2012

# [Grayham DA](http://www.ncbi.nlm.nih.gov/pubmed?term=Grayham%20DA%5BAuthor%5D&cauthor=true&cauthor_uid=9223846). Work-related stress: implications for the employer . [J R Soc Health.](http://www.ncbi.nlm.nih.gov/pubmed/9223846) 1997 Apr;117(2):81-7.

# [Cooper CL](http://www.ncbi.nlm.nih.gov/pubmed?term=Cooper%20CL%5BAuthor%5D&cauthor=true&cauthor_uid=4026744). The stress of work: an overview. [Aviat Space Environ Med.](http://www.ncbi.nlm.nih.gov/pubmed/4026744) 1985 Jul;56(7):627-32.

# [Ronda E](http://www.ncbi.nlm.nih.gov/pubmed?term=Ronda%20E%5BAuthor%5D&cauthor=true&cauthor_uid=10846188), [Alvarez-Dardet C](http://www.ncbi.nlm.nih.gov/pubmed?term=Alvarez-Dardet%20C%5BAuthor%5D&cauthor=true&cauthor_uid=10846188). Work and health: a call for action J Epidemiol community health 2000 Jul;54(7):481.

# [Ulhôa MA](http://www.ncbi.nlm.nih.gov/pubmed?term=Ulhôa%20MA%5BAuthor%5D&cauthor=true&cauthor_uid=22080787), [Marqueze EC](http://www.ncbi.nlm.nih.gov/pubmed?term=Marqueze%20EC%5BAuthor%5D&cauthor=true&cauthor_uid=22080787), [Kantermann T](http://www.ncbi.nlm.nih.gov/pubmed?term=Kantermann%20T%5BAuthor%5D&cauthor=true&cauthor_uid=22080787), [Skene D](http://www.ncbi.nlm.nih.gov/pubmed?term=Skene%20D%5BAuthor%5D&cauthor=true&cauthor_uid=22080787), [Moreno C](http://www.ncbi.nlm.nih.gov/pubmed?term=Moreno%20C%5BAuthor%5D&cauthor=true&cauthor_uid=22080787) When does stress end? Evidence of a prolonged stress reaction in shiftworking truck drivers. [Chronobiol Int.](http://www.ncbi.nlm.nih.gov/pubmed/22080787) 2011 Nov;28(9):810-8

# Bellingrath S, Weigl T, Kudielka BM. [Cortisol dysregulation in school teachers in relation to burnout, vital exhaustion, and effort-reward-imbalance.](http://www.ncbi.nlm.nih.gov/pubmed/18325655)Biol Psychol. 2008 Apr;78(1):104-13

# Zefferino R, Facciorusso A, Lasalvia M, Narciso M, Nuzzaco A, Lucchini R, L'Abbate N. [Salivary markers of work stress in an emergency team of urban police (1 degree step).](http://www.ncbi.nlm.nih.gov/pubmed/17380949) G Ital Med Lav Ergon. 2006 Oct-Dec;28(4):472-7.

# Ricou B, Merlani P. [Burnout in intensive care units.](http://www.ncbi.nlm.nih.gov/pubmed/23346676) Rev Med Suisse. 2012 Dec 12;8(366):2400-2

# Peters L, Cant R, Sellick K, O'Connor M, Lee S, Burney S. Work stress in palliative care nurses a cause for concern? A literature review Int J Palliat Nurs. 2012 Nov;18(11):561-7

# Toh SG, Ang E, Devi MK. Systematic review on the realtionship between the nursing shortage and job satisfaction, stress and burnout levels among nurses in oncology/haematology settings.Int J Evid Based Healthc. 2012 Jun;10(2):126-41

# Vicentic S, Gasic MJ, Milovanovic A, Tosevski DL, Nenadovic M, Damjanovic A, Kostic BD, Jovanovic AA [Burnout, quality of life and emotional profile in general practitioners and psychiatrists.](http://www.ncbi.nlm.nih.gov/pubmed/23324671) Work. 2013 Jan 2. [Epub ahead of print]

# [Vićentić S](http://www.ncbi.nlm.nih.gov/pubmed?term=Vićentić%20S%5BAuthor%5D&cauthor=true&cauthor_uid=20954413), [Jovanović A](http://www.ncbi.nlm.nih.gov/pubmed?term=Jovanović%20A%5BAuthor%5D&cauthor=true&cauthor_uid=20954413), [Dunjić B](http://www.ncbi.nlm.nih.gov/pubmed?term=Dunjić%20B%5BAuthor%5D&cauthor=true&cauthor_uid=20954413), [Pavlović Z](http://www.ncbi.nlm.nih.gov/pubmed?term=Pavlović%20Z%5BAuthor%5D&cauthor=true&cauthor_uid=20954413), [Nenadović M](http://www.ncbi.nlm.nih.gov/pubmed?term=Nenadović%20M%5BAuthor%5D&cauthor=true&cauthor_uid=20954413), [Nenadović N](http://www.ncbi.nlm.nih.gov/pubmed?term=Nenadović%20N%5BAuthor%5D&cauthor=true&cauthor_uid=20954413) Professional stress in general practitioners and psychiatrists--the level of psycologic distress and burnout risk [Vojnosanit Pregl.](http://www.ncbi.nlm.nih.gov/pubmed/20954413) 2010 Sep;67(9):741-6.

# Weibel L, Gabrion I, Aussedat M, Kreutz G. [Work-related stress in an emergency medical dispatch center.](http://www.ncbi.nlm.nih.gov/pubmed/12658250) Ann Emerg Med. 2003 Apr;41(4):500-6.

# [Frey R](http://www.ncbi.nlm.nih.gov/pubmed?term=Frey%20R%5BAuthor%5D&cauthor=true&cauthor_uid=12394962), [Decker K](http://www.ncbi.nlm.nih.gov/pubmed?term=Decker%20K%5BAuthor%5D&cauthor=true&cauthor_uid=12394962), [Reinfried L](http://www.ncbi.nlm.nih.gov/pubmed?term=Reinfried%20L%5BAuthor%5D&cauthor=true&cauthor_uid=12394962), [Klösch G](http://www.ncbi.nlm.nih.gov/pubmed?term=Klösch%20G%5BAuthor%5D&cauthor=true&cauthor_uid=12394962), [Saletu B](http://www.ncbi.nlm.nih.gov/pubmed?term=Saletu%20B%5BAuthor%5D&cauthor=true&cauthor_uid=12394962), [Anderer P](http://www.ncbi.nlm.nih.gov/pubmed?term=Anderer%20P%5BAuthor%5D&cauthor=true&cauthor_uid=12394962), [Semlitsch HV](http://www.ncbi.nlm.nih.gov/pubmed?term=Semlitsch%20HV%5BAuthor%5D&cauthor=true&cauthor_uid=12394962), [Seidler D](http://www.ncbi.nlm.nih.gov/pubmed?term=Seidler%20D%5BAuthor%5D&cauthor=true&cauthor_uid=12394962), [Laggner AN](http://www.ncbi.nlm.nih.gov/pubmed?term=Laggner%20AN%5BAuthor%5D&cauthor=true&cauthor_uid=12394962) Effect of rest on physicians' performance in an emergency department, objectified by electroencephalographic analyses and psychometric tests. [Crit Care Med.](http://www.ncbi.nlm.nih.gov/pubmed/12394962) 2002 Oct;30(10):2322-9.

# Yang Y, Koh D, Ng V, Lee FC, Chan G, Dong F, Chia SE.. [Salivary cortisol levels and work-related stress among emergency department nurses.](http://www.ncbi.nlm.nih.gov/pubmed/11765672) J Occup Environ Med. 2001 Dec;43(12):1011-8

# Bentley MA, Crawford JM, Wilkins JR, Fernandez AR, Studnek JR. [An Assessment of Depression, Anxiety, and Stress Among Nationally Certified EMS Professionals.](http://www.ncbi.nlm.nih.gov/pubmed/23414106) Prehosp Emerg Care. 2013 Feb 15.

# Nakajima Y, Takahashi T, Shetty V, Yamaguchi M. [Patterns of salivary cortisol levels can manifest work stress in emergency care providers.](http://www.ncbi.nlm.nih.gov/pubmed/22350686) J Physiol Sci. 2012 May;62(3):191-7

# Estryn-Behar M, Doppia MA, Guetarni K, Fry C, Machet G, Pelloux P, Aune I, Muster D, Lassaunière JM, Prudhomme C. [Emergency physicians accumulate more stress factors than other physicians-results from the French SESMAT study.](http://www.ncbi.nlm.nih.gov/pubmed/21123828) Emerg Med J. 2011 May;28(5):397-410

# Yang Y, Koh D, Ng V, Lee FC, Chan G, Dong F, Chia SE [Salivary cortisol levels and work-related stress among emergency department nurses.](http://www.ncbi.nlm.nih.gov/pubmed/11765672) J Occup Environ Med. 2001 Dec;43(12):1011-8

# Roth SG, Moore CD. [Work-family fit: the impact of emergency medical services work on the family system.](http://www.ncbi.nlm.nih.gov/pubmed/19731158) Prehosp Emerg Care. 2009 Oct-Dec;13(4):462-8

# Bentley MA, Crawford JM, Wilkins JR, Fernandez AR, Studnek JR. [An Assessment of Depression, Anxiety, and Stress Among Nationally Certified EMS Professionals.](http://www.ncbi.nlm.nih.gov/pubmed/23414106) Prehosp Emerg Care. 2013 Feb 15

# Donnelly E. [Work-related stress and posttraumatic stress in emergency medical services.](http://www.ncbi.nlm.nih.gov/pubmed/22128908) Prehosp Emerg Care. 2012 Jan-Mar;16(1):76-85

# Härmä M. Workhours in relation to work stress, recovery and health. Scand J Work Environ Health. 2006 Dec;32(6):502-14

# Steptoe A, Ussher M. .[Smoking, cortisol and nicotine.](http://www.ncbi.nlm.nih.gov/pubmed/16337291) Int J Psychophysiol. 2006 Mar;59(3):228-35

# Nakajima Y, Takahashi T, Shetty V, Yamaguchi M. [Patterns of salivary cortisol levels can manifest work stress in emergency care providers.](http://www.ncbi.nlm.nih.gov/pubmed/22350686) J Physiol Sci. 2012 May;62(3):191-7

# Backé EM, Kaul G, Klussmann A, Liebers F, Thim C, Massbeck P, Steinberg U. Assessment of salivary cortisol as stress marker in ambulance service personnel: comparison between shifts working on mobile intensive care unit and patient transport ambulance. Int Arch Occup Environ Health. 2009 Oct;82(9):1057-64.

# Baig A, Siddiqui I, Naqvi H, Sabir S, Jabbar J, Shahid M[Correlation of serum cortisol levels and stress among medical doctors working in emergency departments.](http://www.ncbi.nlm.nih.gov/pubmed/16945229) J Coll Physicians Surg Pak. 2006 Sep;16(9):576-80.

# POUGET R. histoire et stress. Psychologie médicale 1992 ; 24 (3) 240-242

# CANNON W.B. (1929) Bodily changes in apin, hunger, fear and rage : An account of recent researches into the functional of emotional excitement. 2nd ed ; New-York : Appleton-Century.

# SELYE H. The stress of life. McGraw-Hill ed. New York, 1976

# Rosch PJ[Reminiscences of Hans Selye, and the birth of "stress".](http://www.ncbi.nlm.nih.gov/pubmed/11227756).Int J Emerg Ment Health. 1999 Winter;1(1):59-66.

# NEBOIT.M. Le stress au travail, 1ère conference internatonale « facteurs psychosociaux dans le travail » 24-26 Août 1998 Copenhague. Hygiène et sécurité au travail, Congrès (compte-rendu). N°172, 3^ème^ trimestre 1998.

# [Mason JW](http://www.ncbi.nlm.nih.gov/pubmed?term=Mason%20JW%5BAuthor%5D&cauthor=true&cauthor_uid=4331538).A re-evaluation of the concept of "non-specificity" in stress theory. [J Psychiatr Res.](http://www.ncbi.nlm.nih.gov/pubmed?cmd=historysearch&querykey=19) 1971 Aug;8(3):323-33.

# Lazarus.Coping theory and research: past, present, and future.[Psychosom Med.](http://www.ncbi.nlm.nih.gov/pubmed?cmd=historysearch&querykey=24) 1993 May-Jun;55(3):234-47.

# Stress, memory and the amygdala.[Roozendaal B](http://www.ncbi.nlm.nih.gov/pubmed?term=Roozendaal%20B%5BAuthor%5D&cauthor=true&cauthor_uid=19469026), [McEwen BS](http://www.ncbi.nlm.nih.gov/pubmed?term=McEwen%20BS%5BAuthor%5D&cauthor=true&cauthor_uid=19469026), [Chattarji S](http://www.ncbi.nlm.nih.gov/pubmed?term=Chattarji%20S%5BAuthor%5D&cauthor=true&cauthor_uid=19469026).[Nat Rev Neurosci.](http://www.ncbi.nlm.nih.gov/pubmed?cmd=historysearch&querykey=2) 2009 Jun;10(6):459-66. doi: 10.1038/nrn2632.

# [Eller NH](http://www.ncbi.nlm.nih.gov/pubmed?term=Eller%20NH%5BAuthor%5D&cauthor=true&cauthor_uid=16824664), [Netterstrøm B](http://www.ncbi.nlm.nih.gov/pubmed?term=Netterstrøm%20B%5BAuthor%5D&cauthor=true&cauthor_uid=16824664), [Hansen AM](http://www.ncbi.nlm.nih.gov/pubmed?term=Hansen%20AM%5BAuthor%5D&cauthor=true&cauthor_uid=16824664) Psychosocial factors at home and at work and levels of salivary cortisol[Biol Psychol.](http://www.ncbi.nlm.nih.gov/pubmed/16824664) 2006 Oct;73(3):280-7

# Feder A, Nestler EJ, Charney DS[Psychobiology and molecular genetics of resilience.](http://www.ncbi.nlm.nih.gov/pubmed/19455174)Nat Rev Neurosci. 2009 Jun;10(6):446-57

# [Miller GE](http://www.ncbi.nlm.nih.gov/pubmed?term=Miller%20GE%5BAuthor%5D&cauthor=true&cauthor_uid=17201569), [Chen E](http://www.ncbi.nlm.nih.gov/pubmed?term=Chen%20E%5BAuthor%5D&cauthor=true&cauthor_uid=17201569), [Zhou ES](http://www.ncbi.nlm.nih.gov/pubmed?term=Zhou%20ES%5BAuthor%5D&cauthor=true&cauthor_uid=17201569). If it goes up, must it come down? Chronic stress and the hypothalamic-pituitary-adrenocortical axis in humans[Psychol Bull.](http://www.ncbi.nlm.nih.gov/pubmed?term=if%20it%20goes%20up%2C%20must%20it%20come%20down%20%3F%20chronic%20stress%20) 2007 Jan;133(1):25-45.

# [Levine S](http://www.ncbi.nlm.nih.gov/pubmed?term=Levine%20S%5BAuthor%5D&cauthor=true&cauthor_uid=15958281).Developmental determinants of sensitivity and resistance to stress[Psychoneuroendocrinology.](http://www.ncbi.nlm.nih.gov/pubmed/15958281) 2005 Nov;30(10):939-46

# Franklin TB, Saab BJ, Mansuy IM [Neural mechanisms of stress resilience and vulnerability.](http://www.ncbi.nlm.nih.gov/pubmed/22958817)Neuron. 2012 Sep 6;75(5):747-61

# [Koolhaas JM](http://www.ncbi.nlm.nih.gov/pubmed?term=Koolhaas%20JM%5BAuthor%5D&cauthor=true&cauthor_uid=21316391), [Bartolomucci A](http://www.ncbi.nlm.nih.gov/pubmed?term=Bartolomucci%20A%5BAuthor%5D&cauthor=true&cauthor_uid=21316391), [Buwalda B](http://www.ncbi.nlm.nih.gov/pubmed?term=Buwalda%20B%5BAuthor%5D&cauthor=true&cauthor_uid=21316391), [de Boer SF](http://www.ncbi.nlm.nih.gov/pubmed?term=de%20Boer%20SF%5BAuthor%5D&cauthor=true&cauthor_uid=21316391), [Flügge G](http://www.ncbi.nlm.nih.gov/pubmed?term=Flügge%20G%5BAuthor%5D&cauthor=true&cauthor_uid=21316391), [Korte SM](http://www.ncbi.nlm.nih.gov/pubmed?term=Korte%20SM%5BAuthor%5D&cauthor=true&cauthor_uid=21316391), [Meerlo P](http://www.ncbi.nlm.nih.gov/pubmed?term=Meerlo%20P%5BAuthor%5D&cauthor=true&cauthor_uid=21316391), [Murison R](http://www.ncbi.nlm.nih.gov/pubmed?term=Murison%20R%5BAuthor%5D&cauthor=true&cauthor_uid=21316391), [Olivier B](http://www.ncbi.nlm.nih.gov/pubmed?term=Olivier%20B%5BAuthor%5D&cauthor=true&cauthor_uid=21316391), [Palanza P](http://www.ncbi.nlm.nih.gov/pubmed?term=Palanza%20P%5BAuthor%5D&cauthor=true&cauthor_uid=21316391), [Richter-Levin G](http://www.ncbi.nlm.nih.gov/pubmed?term=Richter-Levin%20G%5BAuthor%5D&cauthor=true&cauthor_uid=21316391), [Sgoifo A](http://www.ncbi.nlm.nih.gov/pubmed?term=Sgoifo%20A%5BAuthor%5D&cauthor=true&cauthor_uid=21316391), [Steimer T](http://www.ncbi.nlm.nih.gov/pubmed?term=Steimer%20T%5BAuthor%5D&cauthor=true&cauthor_uid=21316391), [Stiedl O](http://www.ncbi.nlm.nih.gov/pubmed?term=Stiedl%20O%5BAuthor%5D&cauthor=true&cauthor_uid=21316391), [van Dijk G](http://www.ncbi.nlm.nih.gov/pubmed?term=van%20Dijk%20G%5BAuthor%5D&cauthor=true&cauthor_uid=21316391), [Wöhr M](http://www.ncbi.nlm.nih.gov/pubmed?term=Wöhr%20M%5BAuthor%5D&cauthor=true&cauthor_uid=21316391), [Fuchs E](http://www.ncbi.nlm.nih.gov/pubmed?term=Fuchs%20E%5BAuthor%5D&cauthor=true&cauthor_uid=21316391) Stress revisited: a critical evaluation of the stress concept[Neurosci Biobehav Rev.](http://www.ncbi.nlm.nih.gov/pubmed/21316391) 2011 Apr;35(5):1291-301

# Hill EE, Zack E, Battaglini C, Viru M, Viru A, Hackney AC[Exercise and circulating cortisol levels: the intensity threshold effect.](http://www.ncbi.nlm.nih.gov/pubmed/18787373)J Endocrinol Invest. 2008 Jul;31(7):587-91.

# [Ulrich-Lai YM](http://www.ncbi.nlm.nih.gov/pubmed?term=Ulrich-Lai%20YM%5BAuthor%5D&cauthor=true&cauthor_uid=19469025), [Herman JP](http://www.ncbi.nlm.nih.gov/pubmed?term=Herman%20JP%5BAuthor%5D&cauthor=true&cauthor_uid=19469025). Neural regulation of endocrine and autonomic stress responses[Nat Rev Neurosci.](http://www.ncbi.nlm.nih.gov/pubmed/19469025) 2009 Jun;10(6):397-409

# Buijs RM, Van Eden CG. The integration of stress by the hypothalamus, amygdala and prefrontal cortex: balance between the autonomic nervous system and the neuroendocrine systemProg Brain Res. 2000;126:117-32

# [Joëls M](http://www.ncbi.nlm.nih.gov/pubmed?term=Joëls%20M%5BAuthor%5D&cauthor=true&cauthor_uid=19339973), [Baram TZ](http://www.ncbi.nlm.nih.gov/pubmed?term=Baram%20TZ%5BAuthor%5D&cauthor=true&cauthor_uid=19339973).The neuro-symphony of stress.

# [Sapolsky RM](http://www.ncbi.nlm.nih.gov/pubmed?term=Sapolsky%20RM%5BAuthor%5D&cauthor=true&cauthor_uid=3527687), [Krey LC](http://www.ncbi.nlm.nih.gov/pubmed?term=Krey%20LC%5BAuthor%5D&cauthor=true&cauthor_uid=3527687), [McEwen BS](http://www.ncbi.nlm.nih.gov/pubmed?term=McEwen%20BS%5BAuthor%5D&cauthor=true&cauthor_uid=3527687).The neuroendocrinology of stress and aging: the glucocorticoid cascade hypothesis.[Endocr Rev.](http://www.ncbi.nlm.nih.gov/pubmed?cmd=historysearch&querykey=5) 1986 Aug;7(3):284-301.

# [Herman JP](http://www.ncbi.nlm.nih.gov/pubmed?term=Herman%20JP%5BAuthor%5D&cauthor=true&cauthor_uid=22450375), [McKlveen JM](http://www.ncbi.nlm.nih.gov/pubmed?term=McKlveen%20JM%5BAuthor%5D&cauthor=true&cauthor_uid=22450375), [Solomon MB](http://www.ncbi.nlm.nih.gov/pubmed?term=Solomon%20MB%5BAuthor%5D&cauthor=true&cauthor_uid=22450375), [Carvalho-Netto E](http://www.ncbi.nlm.nih.gov/pubmed?term=Carvalho-Netto%20E%5BAuthor%5D&cauthor=true&cauthor_uid=22450375), [Myers B](http://www.ncbi.nlm.nih.gov/pubmed?term=Myers%20B%5BAuthor%5D&cauthor=true&cauthor_uid=22450375).Neural regulation of the stress response: glucocorticoid feedback mechanisms.[Braz J Med Biol Res.](http://www.ncbi.nlm.nih.gov/pubmed/22450375) 2012 Apr;45(4):292-8. Epub 2012 Mar 29.

# [HECHTER O](http://www.ncbi.nlm.nih.gov/pubmed?term=HECHTER%20O%5BAuthor%5D&cauthor=true&cauthor_uid=13092959), [SOLOMON MM](http://www.ncbi.nlm.nih.gov/pubmed?term=SOLOMON%20MM%5BAuthor%5D&cauthor=true&cauthor_uid=13092959), [ZAFFARONI A](http://www.ncbi.nlm.nih.gov/pubmed?term=ZAFFARONI%20A%5BAuthor%5D&cauthor=true&cauthor_uid=13092959), [PINCUS G](http://www.ncbi.nlm.nih.gov/pubmed?term=PINCUS%20G%5BAuthor%5D&cauthor=true&cauthor_uid=13092959).Transformation of cholesterol and acetate to adrenal cortical hormones.[Arch Biochem Biophys.](http://www.ncbi.nlm.nih.gov/pubmed?cmd=historysearch&querykey=17) 1953 Sep;46(1):201-14.

# [Kirschbaum C](http://www.ncbi.nlm.nih.gov/pubmed?term=Kirschbaum%20C%5BAuthor%5D&cauthor=true&cauthor_uid=2485862), [Hellhammer DH](http://www.ncbi.nlm.nih.gov/pubmed?term=Hellhammer%20DH%5BAuthor%5D&cauthor=true&cauthor_uid=2485862).Salivary cortisol in psychobiological research: an overview.[Neuropsychobiology.](http://www.ncbi.nlm.nih.gov/pubmed?cmd=historysearch&querykey=20) 1989;22(3):150-69.

# HECHTER O, SOLOMON MM, ZAFFARONI A, PINCUS G[Transformation of cholesterol and acetate to adrenal cortical hormones.](http://www.ncbi.nlm.nih.gov/pubmed/13092959)Arch Biochem Biophys. 1953 Sep;46(1):201-14. No abstract available

# [Adam EK](http://www.ncbi.nlm.nih.gov/pubmed?term=Adam%20EK%5BAuthor%5D&cauthor=true&cauthor_uid=19647372), [Kumari M](http://www.ncbi.nlm.nih.gov/pubmed?term=Kumari%20M%5BAuthor%5D&cauthor=true&cauthor_uid=19647372)Assessing salivary cortisol in large-scale, epidemiological research.[Psychoneuroendocrinology.](http://www.ncbi.nlm.nih.gov/pubmed/19647372) 2009 Nov;34(10):1423-36. doi: 10.1016/j.psyneuen.2009.06.011. Epub 2009 Jul 31

.

# Hellhammer DH, Wüst S, Kudielka BM. [Salivary cortisol as a biomarker in stress research.](http://www.ncbi.nlm.nih.gov/pubmed/19095358)Psychoneuroendocrinology. 2009 Feb;34(2):163-71. doi: 10.1016/j.psyneuen.2008.10.026. Epub 2008 Dec 18. Review.

.

# Castro M, Elias PC, Martinelli CE Jr, Antonini SR, Santiago L, Moreira AC. [Salivary cortisol as a biomarker in stress research.](http://www.ncbi.nlm.nih.gov/pubmed/19095358)Braz J Med Biol Res. 2000 Oct;33(10):1171-5. Review.

# Castagnola M, Picciotti PM, Messana I, Fanali C, Fiorita A, Cabras T, Calò L, Pisano E, Passali GC, Iavarone F, Paludetti G, Scarano E. [Potential applications of human saliva as diagnostic fluid.](http://www.ncbi.nlm.nih.gov/pubmed/22323845)Acta Otorhinolaryngol Ital. 2011 Dec;31(6):347-57. Review.

# [Kaufman E](http://www.ncbi.nlm.nih.gov/pubmed?term=Kaufman%20E%5BAuthor%5D&cauthor=true&cauthor_uid=12097361), [Lamster IB](http://www.ncbi.nlm.nih.gov/pubmed?term=Lamster%20IB%5BAuthor%5D&cauthor=true&cauthor_uid=12097361).The diagnostic applications of saliva--a review. [Crit Rev Oral Biol Med.](http://www.ncbi.nlm.nih.gov/pubmed/12097361) 2002;13(2):197-212.

# Shirtcliff EA, Granger DA, Schwartz E, Curran MJ[Use of salivary biomarkers in biobehavioral research: cotton-based sample collection methods can interfere with salivary immunoassay results.](http://www.ncbi.nlm.nih.gov/pubmed/11087962)

.

# de Weerth C, Graat G, Buitelaar JK, Thijssen JH [Measurement of cortisol in small quantities of saliva.](http://www.ncbi.nlm.nih.gov/pubmed/12651822)Clin Chem. 2003 Apr;49(4):658-60. No abstract available.

# Jessop DS, Turner-Cobb JM[Measurement and meaning of salivary cortisol: a focus on health and disease in children.](http://www.ncbi.nlm.nih.gov/pubmed/17853059)

Stress. 2008 Jan;11(1):1-14. Epub 2007 Jul 16. Review

# Marques AH, Silverman MN, Sternberg EM. [Evaluation of stress systems by applying noninvasive methodologies: measurements of neuroimmune biomarkers in the sweat, heart rate variability and salivary cortisol.](http://www.ncbi.nlm.nih.gov/pubmed/20134204) Neuroimmunomodulation. 2010;17(3):205-8. doi: 10.1159/000258725. Epub 2010 Feb 4. Review. Erratum in: Neuroimmunomodulation. 2010;17(6):395.

# [Kirschbaum C](http://www.ncbi.nlm.nih.gov/pubmed?term=Kirschbaum%20C%5BAuthor%5D&cauthor=true&cauthor_uid=8047637), [Hellhammer DH](http://www.ncbi.nlm.nih.gov/pubmed?term=Hellhammer%20DH%5BAuthor%5D&cauthor=true&cauthor_uid=8047637). Salivary cortisol in psychoneuroendocrine research: recent developments and applications [Psychoneuroendocrinology.](http://www.ncbi.nlm.nih.gov/pubmed?cmd=historysearch&querykey=7) 1994;19(4):313-33

# Levine A, Zagoory-Sharon O, Feldman R, Lewis JG, Weller A [Measuring cortisol in human psychobiological studies.](http://www.ncbi.nlm.nih.gov/pubmed/17055006) Physiol Behav. 2007 Jan 30;90(1):43-53. Epub 2006 Oct 19. Review

# Castagnola M, Picciotti PM, Messana I, Fanali C, Fiorita A, Cabras T, Calò L, Pisano E, Passali GC, Iavarone F, Paludetti G, Scarano E Potential applications of human saliva as diagnostic fluid Acta Otorhinolaryngol Ital. 2011 Dec;31(6):347-57. Review

# [Kirschbaum C](http://www.ncbi.nlm.nih.gov/pubmed?term=Kirschbaum%20C%5BAuthor%5D&cauthor=true&cauthor_uid=8047637), [Hellhammer DH](http://www.ncbi.nlm.nih.gov/pubmed?term=Hellhammer%20DH%5BAuthor%5D&cauthor=true&cauthor_uid=8047637). Salivary cortisol in psychoneuroendocrine research: recent developments and applications.[Psychoneuroendocrinology.](http://www.ncbi.nlm.nih.gov/pubmed/?term=Salivary+cortisol+in+psychoneuroendocrine+research%3A+recent+developments+and+applications.) 1994;19(4):313-33

# [Weibel L](http://www.ncbi.nlm.nih.gov/pubmed?term=Weibel%20L%5BAuthor%5D&cauthor=true&cauthor_uid=8928766), [Spiegel K](http://www.ncbi.nlm.nih.gov/pubmed?term=Spiegel%20K%5BAuthor%5D&cauthor=true&cauthor_uid=8928766), [Follenius M](http://www.ncbi.nlm.nih.gov/pubmed?term=Follenius%20M%5BAuthor%5D&cauthor=true&cauthor_uid=8928766), [Ehrhart J](http://www.ncbi.nlm.nih.gov/pubmed?term=Ehrhart%20J%5BAuthor%5D&cauthor=true&cauthor_uid=8928766), [Brandenberger G](http://www.ncbi.nlm.nih.gov/pubmed?term=Brandenberger%20G%5BAuthor%5D&cauthor=true&cauthor_uid=8928766).Internal dissociation of the circadian markers of the cortisol rhythm in night workers.Source[Am J Physiol.](http://www.ncbi.nlm.nih.gov/pubmed?cmd=historysearch&querykey=11) 1996 Apr;270(4 Pt 1):E608-13.

# KRIEGER DT.in : Endocrine rythms. Comprehensive Endocrinology Series, Krieger DT, ed.NY, Raven Press, 1979.

# Clow A, Thorn L, Evans P, Hucklebridge F. [The awakening cortisol response: methodological issues and significance.](http://www.ncbi.nlm.nih.gov/pubmed/15204030)Stress. 2004 Mar;7(1):29-37. Review.

# Edwards S, Evans P, Hucklebridge F, Clow A. [Association between time of awakening and diurnal cortisol secretory activity.](http://www.ncbi.nlm.nih.gov/pubmed/11403981)Psychoneuroendocrinology. 2001 Aug;26(6):613-22.

# Hennig J, Friebe J, Ryl I, Krämer B, Böttcher J, Netter P. [Upright posture influences salivary cortisol.](http://www.ncbi.nlm.nih.gov/pubmed/10633536)Psychoneuroendocrinology. 2000 Jan;25(1):69-83.

# Bouma EM, Riese H, Ormel J, Verhulst FC, Oldehinkel AJ.[Adolescents' cortisol responses to awakening and social stress; effects of gender, menstrual phase and oral contraceptives. The TRAILS study.](http://www.ncbi.nlm.nih.gov/pubmed/19195792)Psychoneuroendocrinology. 2009 Jul;34(6):884-93. doi: 10.1016/j.psyneuen.2009.01.003. Epub 2009 Feb 4.

# Hill EE, Zack E, Battaglini C, Viru M, Viru A, Hackney AC.[Exercise and circulating cortisol levels: the intensity threshold effect.](http://www.ncbi.nlm.nih.gov/pubmed/18787373)J Endocrinol Invest. 2008 Jul;31(7):587-91.

# . Nonino-Borges CB, Martins Borges R, Bavaresco M, Suen VM, Moreira AC, Marchini JS.[Influence of meal time on salivary circadian cortisol rhythms and weight loss in obese women.](http://www.ncbi.nlm.nih.gov/pubmed/17483007)Nutrition. 2007 May;23(5):385-91.

# . Badrick E, Kirschbaum C, Kumari M.[The relationship between smoking status and cortisol secretion.](http://www.ncbi.nlm.nih.gov/pubmed/17179195)J Clin Endocrinol Metab. 2007 Mar;92(3):819-24. Epub 2006 Dec 19.

# Farag NH, Vincent AS, McKey BS, Al'Absi M, Whitsett TL, Lovallo WR.[Sex differences in the hemodynamic responses to mental stress: Effect of caffeine consumption.](http://www.ncbi.nlm.nih.gov/pubmed/16916429).Psychophysiology. 2006 Jul;43(4):337-43.

# Lovallo WR, Farag NH, Vincent AS, Thomas TL, Wilson MF.[Cortisol responses to mental stress, exercise, and meals following caffeine intake in men and women.](http://www.ncbi.nlm.nih.gov/pubmed/16631247)Pharmacol Biochem Behav. 2006 Mar;83(3):441-7. Epub 2006 May 2.

# Steptoe A, Ussher M.[Smoking, cortisol and nicotine.](http://www.ncbi.nlm.nih.gov/pubmed/16337291)Int J Psychophysiol. 2006 Mar;59(3):228-35. Epub 2005 Dec 6.

# Leproult R, Colecchia EF, L'Hermite-Balériaux M, Van Cauter E.[Transition from dim to bright light in the morning induces an immediate elevation of cortisol levels.](http://www.ncbi.nlm.nih.gov/pubmed/11231993)J Clin Endocrinol Metab. 2001 Jan;86(1):151-7.

# Hennig J, Friebe J, Ryl I, Krämer B, Böttcher J, Netter P.[Upright posture influences salivary cortisol.](http://www.ncbi.nlm.nih.gov/pubmed/10633536)Psychoneuroendocrinology. 2000 Jan;25(1):69-83.

# . Scheer FA, Buijs RM.[Light affects morning salivary cortisol in humans.](http://www.ncbi.nlm.nih.gov/pubmed/10487717)J Clin Endocrinol Metab. 1999 Sep;84(9):3395-8.

# Follenius M, Brandenberger G, Hietter B.[Diurnal cortisol peaks and their relationships to meals.](http://www.ncbi.nlm.nih.gov/pubmed/7202017)J Clin Endocrinol Metab. 1982 Oct;55(4):757-61.

# Gatti R, De Palo EF[An update: salivary hormones and physical exercise.](http://www.ncbi.nlm.nih.gov/pubmed/21129038)Scand J Med Sci Sports. 2011 Apr;21(2):157-69. doi: 10.1111/j.1600-0838.2010.01252.x. Epub 2010 Dec 3. Review.

# Knorr U, Vinberg M, Kessing LV, Wetterslev J.[Salivary cortisol in depressed patients versus control persons: a systematic review and meta-analysis.](http://www.ncbi.nlm.nih.gov/pubmed/20447770)Psychoneuroendocrinology. 2010 Oct;35(9):1275-86.

# Granger DA, Hibel LC, Fortunato CK, Kapelewski CH..[Medication effects on salivary cortisol: tactics and strategy to minimize impact in behavioral and developmental science.](http://www.ncbi.nlm.nih.gov/pubmed/19632788)Psychoneuroendocrinology. 2009 Nov;34(10):1437-48.

# [Kudielka BM](http://www.ncbi.nlm.nih.gov/pubmed?term=Kudielka%20BM%5BAuthor%5D&cauthor=true&cauthor_uid=19041187), [Hellhammer DH](http://www.ncbi.nlm.nih.gov/pubmed?term=Hellhammer%20DH%5BAuthor%5D&cauthor=true&cauthor_uid=19041187), [Wüst S](http://www.ncbi.nlm.nih.gov/pubmed?term=Wüst%20S%5BAuthor%5D&cauthor=true&cauthor_uid=19041187).Why do we respond so differently? Reviewing determinants of human salivary cortisol responses to challenge.[Psychoneuroendocrinology.](http://www.ncbi.nlm.nih.gov/pubmed/19041187) 2009 Jan;34(1):2-18. doi: 10.1016/j.psyneuen.2008.10.004. Epub 2008 Nov 28.

# Kumari M, Badrick E, Chandola T, Adler NE, Epel E, Seeman T, Kirschbaum C, Marmot MG.[Measures of social position and cortisol secretion in an aging population: findings from the Whitehall II study.](http://www.ncbi.nlm.nih.gov/pubmed/19995885)Psychosom Med. 2010 Jan;72(1):27-34. doi: 10.1097/PSY.0b013e3181c85712. Epub 2009 Dec 7.

# Kumari M, Badrick E, Chandola T, Adam EK, Stafford M, Marmot MG, Kirschbaum C, Kivimaki M.[Cortisol secretion and fatigue: associations in a community based cohort.](http://www.ncbi.nlm.nih.gov/pubmed/19497676)Psychoneuroendocrinology. 2009 Nov;34(10):1476-85. doi: 10.1016/j.psyneuen.2009.05.001. Epub 2009 Jun 3.

# Kudielka BM, Kirschbaum C.[Sex differences in HPA axis responses to stress: a review.](http://www.ncbi.nlm.nih.gov/pubmed/15740829)Biol Psychol. 2005 Apr;69(1):113-32. Epub 2004 Dec 25. Review. No abstract available.

# Kudielka BM, Kirschbaum C. [Sex differences in HPA axis responses to stress: a review.](http://www.ncbi.nlm.nih.gov/pubmed/15740829)Biol Psychol. 2005 Apr;69(1):113-32. Epub 2004 Dec 25. Review

# Paris JJ, Franco C, Sodano R, Freidenberg B, Gordis E, Anderson DA, Forsyth JP, Wulfert E, Frye CA. [Sex differences in salivary cortisol in response to acute stressors among healthy participants, in recreational or pathological gamblers, and in those with posttraumatic stress disorder.](http://www.ncbi.nlm.nih.gov/pubmed/19538960)Horm Behav. 2010 Jan;57(1):35-45. doi: 10.1016/j.yhbeh.2009.06.003. Epub 2009 Jun 16. Review.

# Kirschbaum C, Kudielka BM, Gaab J, Schommer NC, Hellhammer DH. [Impact of gender, menstrual cycle phase, and oral contraceptives on the activity of the hypothalamus-pituitary-adrenal axis.](http://www.ncbi.nlm.nih.gov/pubmed/10204967)Psychosom Med. 1999 Mar-Apr;61(2):154-62

# [Swanson NG](http://www.ncbi.nlm.nih.gov/pubmed?term=Swanson%20NG%5BAuthor%5D&cauthor=true&cauthor_uid=10808656) Working women and stress[J Am Med Womens Assoc.](http://www.ncbi.nlm.nih.gov/pubmed/10808656) 2000 Spring;55(2):76-9.

# Liening SH, Stanton SJ, Saini EK, Schultheiss OC. [Salivary testosterone, cortisol, and progesterone: two-week stability, interhormone correlations, and effects of time of day, menstrual cycle, and oral contraceptive use on steroid hormone levels.](http://www.ncbi.nlm.nih.gov/pubmed/19833145)Physiol Behav. 2010 Jan 12;99(1):8-16

# Gatti R, De Palo EF. [An update: salivary hormones and physical exercise.](http://www.ncbi.nlm.nih.gov/pubmed/21129038)Scand J Med Sci Sports. 2011 Apr;21(2):157-69

# Knorr U, Vinberg M, Kessing LV, Wetterslev J[Salivary cortisol in depressed patients versus control persons: a systematic review and meta-analysis.](http://www.ncbi.nlm.nih.gov/pubmed/20447770)Psychoneuroendocrinology. 2010 Oct;35(9):1275-86

# [Sapolsky RM](http://www.ncbi.nlm.nih.gov/pubmed?term=Sapolsky%20RM%5BAuthor%5D&cauthor=true&cauthor_uid=3527687), [Krey LC](http://www.ncbi.nlm.nih.gov/pubmed?term=Krey%20LC%5BAuthor%5D&cauthor=true&cauthor_uid=3527687), [McEwen BS](http://www.ncbi.nlm.nih.gov/pubmed?term=McEwen%20BS%5BAuthor%5D&cauthor=true&cauthor_uid=3527687). The neuroendocrinology of stress and aging: the glucocorticoid cascade hypothesis[Endocr Rev.](http://www.ncbi.nlm.nih.gov/pubmed?cmd=historysearch&querykey=5) 1986 Aug;7(3):284-301

# [Herman JP](http://www.ncbi.nlm.nih.gov/pubmed?term=Herman%20JP%5BAuthor%5D&cauthor=true&cauthor_uid=14596810), [Figueiredo H](http://www.ncbi.nlm.nih.gov/pubmed?term=Figueiredo%20H%5BAuthor%5D&cauthor=true&cauthor_uid=14596810), [Mueller NK](http://www.ncbi.nlm.nih.gov/pubmed?term=Mueller%20NK%5BAuthor%5D&cauthor=true&cauthor_uid=14596810), [Ulrich-Lai Y](http://www.ncbi.nlm.nih.gov/pubmed?term=Ulrich-Lai%20Y%5BAuthor%5D&cauthor=true&cauthor_uid=14596810), [Ostrander MM](http://www.ncbi.nlm.nih.gov/pubmed?term=Ostrander%20MM%5BAuthor%5D&cauthor=true&cauthor_uid=14596810), [Choi DC](http://www.ncbi.nlm.nih.gov/pubmed?term=Choi%20DC%5BAuthor%5D&cauthor=true&cauthor_uid=14596810), [Cullinan WE](http://www.ncbi.nlm.nih.gov/pubmed?term=Cullinan%20WE%5BAuthor%5D&cauthor=true&cauthor_uid=14596810) Central mechanisms of stress integration: hierarchical circuitry controlling hypothalamo-pituitary-adrenocortical responsiveness[Front Neuroendocrinol.](http://www.ncbi.nlm.nih.gov/pubmed/14596810) 2003 Jul;24(3):151-80

# [Buijs RM](http://www.ncbi.nlm.nih.gov/pubmed?term=Buijs%20RM%5BAuthor%5D&cauthor=true&cauthor_uid=11105644), [Van Eden CG](http://www.ncbi.nlm.nih.gov/pubmed?term=Van%20Eden%20CG%5BAuthor%5D&cauthor=true&cauthor_uid=11105644). The integration of stress by the hypothalamus, amygdala and prefrontal cortex: balance between the autonomic nervous system and the neuroendocrine system[Prog Brain Res.](http://www.ncbi.nlm.nih.gov/pubmed/11105644) 2000;126:117-32.

# [Sapolsky RM](http://www.ncbi.nlm.nih.gov/pubmed?term=Sapolsky%20RM%5BAuthor%5D&cauthor=true&cauthor_uid=8701325).Why stress is bad for your brain.[Science.](http://www.ncbi.nlm.nih.gov/pubmed/?term=why+stress+is+bad+for+your+brain) 1996 Aug 9;273(5276):749-50.

# [Lupien SJ](http://www.ncbi.nlm.nih.gov/pubmed?term=Lupien%20SJ%5BAuthor%5D&cauthor=true&cauthor_uid=19401723), [McEwen BS](http://www.ncbi.nlm.nih.gov/pubmed?term=McEwen%20BS%5BAuthor%5D&cauthor=true&cauthor_uid=19401723), [Gunnar MR](http://www.ncbi.nlm.nih.gov/pubmed?term=Gunnar%20MR%5BAuthor%5D&cauthor=true&cauthor_uid=19401723), [Heim C](http://www.ncbi.nlm.nih.gov/pubmed?term=Heim%20C%5BAuthor%5D&cauthor=true&cauthor_uid=19401723).Effects of stress throughout the lifespan on the brain, behaviour and cognition.[Nat Rev Neurosci.](http://www.ncbi.nlm.nih.gov/pubmed/19401723) 2009 Jun;10(6):434-45

# Viinamäki H, Lehto SM, Palvimo JJ, Harvima I, Valkonen-Korhonen M, Koivumaa-Honkanen H, Hintikka J, Kirsi H, Niskanen L.[[Glucocorticoids and aetiopathogenesis of depression].](http://www.ncbi.nlm.nih.gov/pubmed/22724317)Duodecim. 2012;128(10):1022-9. Review. Finnish.

# Gold PW, Chrousos GP.[Organization of the stress system and its dysregulation in melancholic and atypical depression: high vs low CRH/NE states.](http://www.ncbi.nlm.nih.gov/pubmed/11920153)Mol Psychiatry. 2002;7(3):254-75. Review.

# O'Keane V, Frodl T, Dinan TG.[A review of Atypical depression in relation to the course of depression and changes in HPA axis organization.](http://www.ncbi.nlm.nih.gov/pubmed/22497986)Psychoneuroendocrinology. 2012 Oct;37(10):1589-99.

# Maina G.[[Psychosocial factors and cardiovascular diseases: the neuroendocrine pathways].](http://www.ncbi.nlm.nih.gov/pubmed/23405611).G Ital Med Lav Ergon. 2012 Jul-Sep;34(3 Suppl):170-3. Italian.

# Hamer M, O'Donnell K, Lahiri A, Steptoe A.[Salivary cortisol responses to mental stress are associated with coronary artery calcification in healthy men and women.](http://www.ncbi.nlm.nih.gov/pubmed/19744954)Eur Heart J. 2010 Feb;31(4):424-9.

# Das S, O'Keefe JH.[Behavioral cardiology: recognizing and addressing the profound impact of psychosocial stress on cardiovascular health.](http://www.ncbi.nlm.nih.gov/pubmed/18775114)Curr Hypertens Rep. 2008 Oct;10(5):374-81.

# Kivimäki M, Virtanen M, Elovainio M, Kouvonen A, Väänänen A, Vahtera J [Work stress in the etiology of coronary heart disease--a meta-analysis.](http://www.ncbi.nlm.nih.gov/pubmed/17173200)Scand J Work Environ Health. 2006 Dec;32(6):431-42.

# Saner H.[[Stress as a cardiovascular risk factor].](http://www.ncbi.nlm.nih.gov/pubmed/16218493)Ther Umsch. 2005 Sep;62(9):597-602. Review. German.

# Csef H, Hefner J.[[Stress and myocardial infarction].](http://www.ncbi.nlm.nih.gov/pubmed/15832759)MMW Fortschr Med. 2005 Mar 31;147(13):33-5. Review. German.

# Chandola T, Brunner E, Marmot M.[Chronic stress at work and the metabolic syndrome: prospective study.](http://www.ncbi.nlm.nih.gov/pubmed/16428252)BMJ. 2006 Mar 4;332(7540):521-5. Epub 2006 Jan 20.

# [Anagnostis P](http://www.ncbi.nlm.nih.gov/pubmed?term=Anagnostis%20P%5BAuthor%5D&cauthor=true&cauthor_uid=19470627), [Athyros VG](http://www.ncbi.nlm.nih.gov/pubmed?term=Athyros%20VG%5BAuthor%5D&cauthor=true&cauthor_uid=19470627), [Tziomalos K](http://www.ncbi.nlm.nih.gov/pubmed?term=Tziomalos%20K%5BAuthor%5D&cauthor=true&cauthor_uid=19470627), [Karagiannis A](http://www.ncbi.nlm.nih.gov/pubmed?term=Karagiannis%20A%5BAuthor%5D&cauthor=true&cauthor_uid=19470627), [Mikhailidis DP](http://www.ncbi.nlm.nih.gov/pubmed?term=Mikhailidis%20DP%5BAuthor%5D&cauthor=true&cauthor_uid=19470627) Clinical review: The pathogenetic role of cortisol in the metabolic syndrome: a hypothesis.. . [J Clin Endocrinol Metab.](http://www.ncbi.nlm.nih.gov/pubmed?db=pubmed&cmd=historysearch&querykey=1) 2009 Aug;94(8):2692-701.

# Petersen H, Kecklund G, D'Onofrio P, Nilsson J, Åkerstedt T.[Stress vulnerability and the effects of moderate daily stress on sleep polysomnography and subjective sleepiness.](http://www.ncbi.nlm.nih.gov/pubmed/22835074)J Sleep Res. 2013 Feb;22(1):50-7. doi: 10.1111/j.1365-2869.2012.01034.x. Epub 2012 Jul 27.

# Dahlgren A, Kecklund G, Akerstedt T.[Different levels of work-related stress and the effects on sleep, fatigue and cortisol.](http://www.ncbi.nlm.nih.gov/pubmed/16161710)Scand J Work Environ Health. 2005 Aug;31(4):277-85.

# Söderström M, Ekstedt M, Akerstedt T, Nilsson J, Axelsson J [Sleep and sleepiness in young individuals with high burnout scores.](http://www.ncbi.nlm.nih.gov/pubmed/15586790).Sleep. 2004 Nov 1;27(7):1369-77.

# Kelly JJ, Mangos G, Williamson PM, Whitworth JA.[Cortisol and hypertension.](http://www.ncbi.nlm.nih.gov/pubmed/9809193)Clin Exp Pharmacol Physiol Suppl. 1998 Nov;25:S51-6. Review.

# Sajadinejad MS, Asgari K, Molavi H, Kalantari M, Adibi P.[Psychological issues in inflammatory bowel disease: an overview.](http://www.ncbi.nlm.nih.gov/pubmed/22778720)Gastroenterol Res Pract. 2012;2012:106502. doi: 10.1155/2012/106502. Epub 2012 Jun 21.

# [Alhazzani W](http://www.ncbi.nlm.nih.gov/pubmed?term=Alhazzani%20W%5BAuthor%5D&cauthor=true&cauthor_uid=22354363), [Alshahrani M](http://www.ncbi.nlm.nih.gov/pubmed?term=Alshahrani%20M%5BAuthor%5D&cauthor=true&cauthor_uid=22354363), [Moayyedi P](http://www.ncbi.nlm.nih.gov/pubmed?term=Moayyedi%20P%5BAuthor%5D&cauthor=true&cauthor_uid=22354363), [Jaeschke R](http://www.ncbi.nlm.nih.gov/pubmed?term=Jaeschke%20R%5BAuthor%5D&cauthor=true&cauthor_uid=22354363).Stress ulcer prophylaxis in critically ill patients: review of the evidence. [Pol Arch Med Wewn.](http://www.ncbi.nlm.nih.gov/pubmed/22354363) 2012;122(3):107-14..

# Maniam J, Morris MJ.[The link between stress and feeding behaviour.](http://www.ncbi.nlm.nih.gov/pubmed/22710442)Neuropharmacology. 2012 Jul;63(1):97-110. doi: 10.1016/j.neuropharm.2012.04.017. Epub 2012 Apr 24. Review.

# Wasser SK, Sewall G, Soules MR.[Psychosocial stress as a cause of infertility.](http://www.ncbi.nlm.nih.gov/pubmed/8458480)Fertil Steril. 1993 Mar;59(3):685-9.

# [Soucek M](http://www.ncbi.nlm.nih.gov/pubmed?term=Soucek%20M%5BAuthor%5D&cauthor=true&cauthor_uid=11395876), [Kára T](http://www.ncbi.nlm.nih.gov/pubmed?term=Kára%20T%5BAuthor%5D&cauthor=true&cauthor_uid=11395876). [Stress-induced hypertension and diabetes mellitus].[Article in Czech] [Vnitr Lek.](http://www.ncbi.nlm.nih.gov/pubmed?cmd=historysearch&querykey=17) 2001 May;47(5):315-9.

# . [McCray CJ](http://www.ncbi.nlm.nih.gov/pubmed?term=McCray%20CJ%5BAuthor%5D&cauthor=true&cauthor_uid=21094920), [Agarwal SK](http://www.ncbi.nlm.nih.gov/pubmed?term=Agarwal%20SK%5BAuthor%5D&cauthor=true&cauthor_uid=21094920).Stress and autoimmunity.[Immunol Allergy Clin North Am.](http://www.ncbi.nlm.nih.gov/pubmed?cmd=historysearch&querykey=24) 2011 Feb;31(1):1-18.

# Kouvonen A, Vahtera J, Oksanen T, Pentti J, Väänänen AK, Heponiemi T, Salo P, Virtanen M, Kivimäki M. [Chronic workplace stress and insufficient physical activity: a cohort study.](http://www.ncbi.nlm.nih.gov/pubmed/23014593)Occup Environ Med. 2013 Jan;70(1):3-8

# [Bauer ME](http://www.ncbi.nlm.nih.gov/pubmed?term=Bauer%20ME%5BAuthor%5D&cauthor=true&cauthor_uid=19047801) Chronic stress and immunosenescence: a review. [Neuroimmunomodulation.](http://www.ncbi.nlm.nih.gov/pubmed/19047801) 2008;15(4-6):241-50

# [Albanidou-Farmaki E](http://www.ncbi.nlm.nih.gov/pubmed?term=Albanidou-Farmaki%20E%5BAuthor%5D&cauthor=true&cauthor_uid=18441503), [Poulopoulos AK](http://www.ncbi.nlm.nih.gov/pubmed?term=Poulopoulos%20AK%5BAuthor%5D&cauthor=true&cauthor_uid=18441503), [Epivatianos A](http://www.ncbi.nlm.nih.gov/pubmed?term=Epivatianos%20A%5BAuthor%5D&cauthor=true&cauthor_uid=18441503), [Farmakis K](http://www.ncbi.nlm.nih.gov/pubmed?term=Farmakis%20K%5BAuthor%5D&cauthor=true&cauthor_uid=18441503), [Karamouzis M](http://www.ncbi.nlm.nih.gov/pubmed?term=Karamouzis%20M%5BAuthor%5D&cauthor=true&cauthor_uid=18441503), [Antoniades D](http://www.ncbi.nlm.nih.gov/pubmed?term=Antoniades%20D%5BAuthor%5D&cauthor=true&cauthor_uid=18441503).Increased anxiety level and high salivary and serum cortisol concentrations in patients with recurrent aphthous stomatitis.[Tohoku J Exp Med.](http://www.ncbi.nlm.nih.gov/pubmed/?term=1.%09Increased+anxiety+level+and+high+salivary+and+serum+cortisol+concentrations+in+patients+with+recurrent+aphthous+stomatitis) 2008 Apr;214(4):291-6.

# Homann D, Stefanello JM, Góes SM, Breda CA, Paiva Edos S, Leite N. [Stress perception and depressive symptoms: functionality and impact on the quality of life of women with fibromyalgia.](http://www.ncbi.nlm.nih.gov/pubmed/22641587)Rev Bras Reumatol. 2012 May-Jun;52(3):319-30. English, Portuguese.

# Menzies V, Lyon DE, Elswick RK Jr, Montpetit AJ, McCain NL. [Psychoneuroimmunological relationships in women with fibromyalgia.](http://www.ncbi.nlm.nih.gov/pubmed/22174319)Biol Res Nurs. 2013 Apr;15(2):219-25. doi: 10.1177/1099800411424204. Epub 2011 Dec 15.

# Godbout JP, Glaser R.[Stress-induced immune dysregulation: implications for wound healing, infectious disease and cancer.](http://www.ncbi.nlm.nih.gov/pubmed/18040814)J Neuroimmune Pharmacol. 2006 Dec;1(4):421-7. Epub 2006 Aug 10. Review.

# Glaser R, Kiecolt-Glaser JK.[Stress-induced immune dysfunction: implications for health.](http://www.ncbi.nlm.nih.gov/pubmed/15738954)Nat Rev Immunol. 2005 Mar;5(3):243-51.

# . Felton JS.[Burnout as a clinical entity--its importance in health care workers.](http://www.ncbi.nlm.nih.gov/pubmed/9800422)Occup Med (Lond). 1998 May;48(4):237-50. Review.[Methods Mol Biol.](http://www.ncbi.nlm.nih.gov/pubmed?cmd=historysearch&querykey=32) 2012;934:39-75

# [Nakata A](http://www.ncbi.nlm.nih.gov/pubmed?term=Nakata%20A%5BAuthor%5D&cauthor=true&cauthor_uid=22933140).Psychosocial job stress and immunity: a systematic review.[Methods Mol Biol.](http://www.ncbi.nlm.nih.gov/pubmed?cmd=historysearch&querykey=32) 2012;934:39-75

# [Verhaeghe J](http://www.ncbi.nlm.nih.gov/pubmed?term=Verhaeghe%20J%5BAuthor%5D&cauthor=true&cauthor_uid=22753184), [Van Den Eede F](http://www.ncbi.nlm.nih.gov/pubmed?term=Van%20Den%20Eede%20F%5BAuthor%5D&cauthor=true&cauthor_uid=22753184), [Van Den Ameele H](http://www.ncbi.nlm.nih.gov/pubmed?term=Van%20Den%20Ameele%20H%5BAuthor%5D&cauthor=true&cauthor_uid=22753184), [Sabbe BG](http://www.ncbi.nlm.nih.gov/pubmed?term=Sabbe%20BG%5BAuthor%5D&cauthor=true&cauthor_uid=22753184).[Neuro-endocrine correlates of burnout].[Tijdschr Psychiatr.](http://www.ncbi.nlm.nih.gov/pubmed?cmd=historysearch&querykey=33) 2012;54(6):517-26.

# [Ricou B](http://www.ncbi.nlm.nih.gov/pubmed?term=Ricou%20B%5BAuthor%5D&cauthor=true&cauthor_uid=23346676), [Merlani P](http://www.ncbi.nlm.nih.gov/pubmed?term=Merlani%20P%5BAuthor%5D&cauthor=true&cauthor_uid=23346676) [Burnout in intensive care units].. [Rev Med Suisse.](http://www.ncbi.nlm.nih.gov/pubmed/23346676) 2012 Dec 12;8(366):2400-2, 2404.

# [Ronda E](http://www.ncbi.nlm.nih.gov/pubmed?term=Ronda%20E%5BAuthor%5D&cauthor=true&cauthor_uid=10846188), [Alvarez-Dardet C](http://www.ncbi.nlm.nih.gov/pubmed?term=Alvarez-Dardet%20C%5BAuthor%5D&cauthor=true&cauthor_uid=10846188) Work and health: a call for action.[J Epidemiol Community Health.](http://www.ncbi.nlm.nih.gov/pubmed/10846188) 2000 Jul;54(7):481.

# CHAMOUX A, PARIS C, MERLE JL, Regeard Evaluation du stress professionnel par échelle visuelle analogiqueArch mal prof 2000 61, (7) : 524

# Lesage FX, Chamoux AUtilisation de l’echelle visuelle analogique dans l’évaluation du stress au travail : limites et perspevtives. Revue de la littératureArch mal prof environ 2008 69 (6-7)

# Lesage FX, Chamoux A, Beriot S.Stabilité de l’EVA dans l’évaluation du stress au travailArch mal prof environ. 2009 (70) 6

# LESAGE FX, LESAGE S, BERIOT S, Chamoux Mesure du stress par échelle visuelle analogique EVA : validité externe versus le modèle de karasek et le modèle de stress perçu PSS14Arch Mal Prof Environ 2010 71 (3)

# Lesage FX, Beriot S. Validity of occupational stress assessment using a visual scale analogue OCCup Med 2011 61 (6)

# Hansez I.Evaluation des outils : stress perçu positif et negative (SPPN) INRS : documenst pour le médecin du travail deuxième trimestre 2011 N°126, p.209

# De KEYSER V., Hansez I.Vers une perspective transactionnelle du stress au travail : pistes d’évaluation méthodologiques Cah. Med. Travail 1996 33(3)

# Barbier M., Hansez I. Le questionnaire de stress positif et négatif : évaluation statistique et structurelle. Actes du 15ème congres de psychologie du travail et des organisations de langue française

# Costa, P.T., McCrae, R.R., & Rolland, J-P. (1998). Inventaire de Personnalité Révisé. Paris : Les Éditions du Centre de Psychologie Appliquée (ECPA).
