# Supplementary material for: Stress and salivary cortisol in emergency medical dispatchers: A randomized shifts control trial
Source: PLoS One. 2017 May 15;12(5):e0177094. doi: 10.1371/journal.pone.0177094 (PMC5432068; doi:10.1371/journal.pone.0177094)

**Clinical research protocol**

Favourable opinion issued by the IRB on .../.../...

ANSM approval granted on .../.../...

Version **2.3 of** 13/03/2014

IDRCB no.: 2013-A01725-40

**Descriptive study of biological stress and perceived stress**

**in PHONE OPERATORS AND PHYSICIANS**

**FROM AN EMERGENCY MEDICAL DISPATCHER**

**TABLE OF CONTENTS**

**1. PROCOTOL SUMMARY**

**2. Scientific justification FOR and general description of research**

**3. Research objectives**

**4. Research design**

**5. INCLUSION and exclusion criteria for research participaNTS**

**6. Description of exposure**

**7. Safety evaluation**

**8. Statistics**

**9. Right of access to data and source materials**

**10. Quality control and assurance**

**11. Ethical and regulatory considerations**

**12. Data processing and document and data storage as relates to research**

**13. Funding**

**14. Regulations governing publication**

**15. List of annexes**

**16. BIBLIOGRAPHY**

**List of abbreviations used**

|  |  |
| --- | --- |
|  |  |
| ANSM | French National Medicinal Drug and Healthcare Product Safety Agency |
| CRA | Clinical research associate |
| ARM | Medical dispatch assistant |
| AVC | Stroke |
| GCP | Good clinical practice |
| CNIL | French National Commission for Information Technology and Civil Liberties |
| IRB | Institutional review board (Comité de Protection des Personnes) |
| CRF | Case report form |
| SAE | Serious adverse event |
| Unexpected SAE | Unexpected serious adverse event |
| ICH | International Conference on Harmonisation |
| ECM | External cardiac massage |
| RM | Reference methodology |
| APO | Acute pulmonary oedema |
| SMUR | French Mobile Emergency and Resuscitation Services |
| PNOSI (questionnaire) | Positive and Negative Occupational Stress Inventory |
| SUSAR | Suspected unexpected serious adverse reaction |
|  |  |
|  |  |
|  |  |

**2. Scientific justification FOR and general description of research:**

**Presentation of current scientific data on stress in dispatch centres**

Occupational stress is no longer an abstract concept. A number of studies have been carried out on this subject although few have evaluated stress in Centre 15 (dispatch centre) employees.

This protocol begins with a brief overview of the history and pathophysiology of stress followed by the benefits of using cortisol levels in studies on stress and lastly the link between physiology and pathophysiology.

**2.1 Occupational stress**

Stress has been a constant concern for several years now and even more so in the workplace environment, which has figured more largely in people's lives. Companies have started to realise the losses incurred as a result of stress: employee illness, absenteeism, drops in productivity (2). According to the European Agency for Health and Safety at Work, occupational stress affects 22% of all workers (using the 2005 figures) and is responsible for 50-60% of all instances of work absenteeism (3). Coming to grips with occupational stress is therefore an absolute must in a financial sense, as the cost of stress is currently 20 billion euros for the whole Europe.

Extending the concept of biological stress to include mental stress has meant more studies on working conditions can be conducted (4, 5, 6, 7, 8). Occupational stress and its repercussions remain largely unexplored, particularly as relate to medical and paramedical professionals (9, 10, 11, 12, 13).

**2.1.1 Occupational stress in the emergency services field**

The medical field is a socio-professional environment where stress is a permanent fixture, although this rings even more true for emergency units (14, 15, 16, 17, 18, 19, 20, 21, 22, 23) where doctors and paramedical professionals are given a very important job to perform and have to work 24 hour shifts (for doctors) and more often than not 12 hour shifts for paramedical staff (24).

There are two intrinsic characteristics in the emergency unit workload which give rise to its stressful nature: firstly the fact employees have to deal with emergencies, i.e. time pressure which is stressful enough in itself, but then there is the fact that it is impossible to anticipate or plan for what the workload is going to be (25). One of the most stressful areas in an emergency unit or as a paramedical professional is the management of dispatch calls in Centre 15.

When calls from Centre 15 come in, doctors and paramedical staff have to deal with acute stress. They firstly have to expend mental energy to retain all of the major information regarding the support services they are to provide and then have to decide how to best handle the situation at hand and what resources should be used, such as dispatching a private ambulance or getting an SMUR (French Mobile Emergency and Resuscitation Services) team involved.

Studies have been conducted with the aim of assessing the level of occupational stress in current hospital practitioners (12, 13, 14), whilst in more recent years a number of studies have been more focused on stress in emergency unit medical and paramedical professionals (17, 18), with said studies frequently using saliva cortisol testing to quantify the exact level of stress (22, 25, 26, 27).

**2.1.2 Stress as relates to dispatch work**

Studies on stress in dispatch centres are even less common. To the authors' knowledge only one single study has been conducted on paramedical professionals and helpline advisers. This study focused mostly on stress incurred over the working shift, with a comparison drawn between cortisol assays carried out during working days and days off (1).

To the authors' knowledge no studies are available on acute stress in dispatch centre emergency call assistants during dispatch calls. Furthermore no studies have been conducted on acute or chronic stress in emergency unit doctors.

No study has provided a subjective or objective means of gauging stress for emergency dispatch workers. However stress causes both a physiological and psychological reaction in organisms and as such psychological and physiological approaches would appear to be both inseparable and complementary to the reliable assessment of stress response levels.

The purpose of this study is therefore to assess the biological and psychological response to occupational stress in Centre 15 paramedical professionals and doctors. This evaluation is both objective and subjective and is based on biological measurements taken as well as responses to questionnaires. Age is introduced as an explanatory variable based on the assumption that seniority and experience are capable of influencing the response to stress.

**2.2. Introduction to understanding stress mechanisms**

**2.2.1. History and definition of stress**

**History of stress🡪🡪**

Stress is an ancient concept, having been described in antiquity in the annuals kept by Greek and Latin chroniclers.

In the 17th century, "stress" in English came to mean "a state of distress" and conveyed an idea of suffering, oppression, the hardship of life, deprivation, fatigue and more generally speaking the concept of adversity.

It was not until after the 18th century that the word took on its more modern definition of force or pressure or even a strong influence acting on a physical object or person.

During the 19th century, current reasoning saw the introduction of the concept of aggressive living conditions (stress) as capable of producing negative physical and mental effects (strain).

In the 1850s the French physiologist Claude Bernard, a pioneer in experimental medicine, showed that one of the key properties to living creatures was their ability to maintain the stability of their internal environment regardless of their external environment and changes happening thereto (29).

In 1910 a cardiologist reported that hard labour combined with a heavy sense of responsibility were the cause of torment and anxiety which could lead to medical problems (Angina pectoris - The Lancet, 1910).

In 1928 the American neurophysiologist Walter Bradford Cannon (1871-1945) emphasised the role emotional factors played in the development of diseases and viewed the word "stress" as representative of both a physiological and psychological concept. Cannon was the first to draw attention to how important the length of the attack was when differentiating between what he called "fixed stress" and "variable stress" (30).

However it was Hans Selye who was the first to use the word "stress" in medicine with his discovery of general adaptation syndrome (31). “Occupational stress” as a concern in the literature is even more recent, being first noted in 1965 (32).

**Defining stress**

Etymologically speaking, the word "stress" comes from the Latin “stringer”: to place under tension. Stress can be defined in a number of ways depending on the source consulted:

The Encyclopædia Universalis:

An organism's reaction to any sort of stressor or trauma. In fact, "stress" has gradually become synonymous with the emotion, the meaning of the latter having being considerably weakened. It is also more specifically used to refer to tension created by unresolved internal conflicts or insoluble situations causing feelings of anxiety or even neurotic states.

The Larousse Dictionary:

An organism's response to physiological and psychological stressors as well as emotions (both pleasant and/or otherwise) which would require adaptation.

The Flammarion medical dictionary:

Aggression against a living organism; by extension: the biological and psychological reaction an organism experiences faced with a new situation, regardless of whether this situation is dangerous or pleasant.

French Wikipedia entry:

**Stress** (from the old French *déstresse* via English) is, in biology, the whole of the response an organism experiences when subjected to pressure or constraints from its surrounding environment. These responses are always based on the individual's perceptions of the pressures they are faced with. The medical definition of stress is a complex sequence of events which cause physiological and psychosomatic responses [in an organism]. By extension all of these events can be qualified as a form of stress. In everyday language there is positive stress (eustress) and negative stress (distress). Stress is different to anxiety - an emotion – as it is a response mechanism which may cause various emotions, one of which is anxiety.

1. **Selye's definition of stress**

This concept was introduced in the 1930s, individualising common physiological reactions to all types of aggression. The whole of the responses to stress was, according to Selye, a non-specific response caused by physical stressors. These reactions were stereotyped regardless of the stress factor in question.

The organism's response was called the general adaptation syndrome (GAS), which over time was developed to include three phases:

- The reaction to the alarm or alert,
- The resistance or adaptation stage
- And the exhaustion stage.

Taking Selye's definition, stress is "the organism's response to any stimulation it experiences." It is characterised by a linear, non-specific physiological reaction to the stressor.

The evolution behind the concept of stress over time can therefore be seen, starting off as a triggering event per Cannon and developing to something much more subsequent per Selye.

Since the start of the 1990s Selye's theory of stress has been revised and expounded upon by a number of scientists. The non-specificity of the stress response has been challenged by demonstrating the vast number of variations in the stress response itself based on the stressor, but mostly in the experience itself and what the individual is going through. The first person to contradict Hans Selye was John Mason, who demonstrated the importance of emotional activity as part of the intensity of stress responses (34).

Coping theories presented by neuropsychologists then showed that cognitive assessment is crucial for determining emotional activation and the resulting physiology. These advances have led to the current definition of stress (35) which takes into account the vast inter-individual variability noted during stress responses (36) both with respect to genetic heritage (38) and personal history (39, 40, 41).

As such stress may be deemed to be a fundamentally psychobiological concept, with stressors acting via cognitive and emotional processes with all mental phenomena effectively having a cerebral and biological connection (42).

1. **The current definition of stress**

The most frequently used definition of stress is as follows: "stress is a dynamic state reflecting a psychophysiological imbalance between estimated resources and perceived demands during testing situations" (42).

1. **Definition of occupational stress**

On 8 October 2004 a European agreement defined occupational stress for the purposes of raising awareness within the workplace, developing anti-stress measures and defining both employers' and employees' responsibilities in this regard.

The definition given is as follows: "stress is an imbalance between constraints imposed by [an individual's] surrounding environment and their perceptions of the resources they have to cope with [the demands of] said environment."

The full definition was provided by Schaufeli in 1998 (43): "stress is a temporary state of functional maladjustment in which mental, physiological and behavioural symptoms manifest a not entirely successful attempt to adapt, which are then presented over the short/medium term in a wide variety of symptoms - with burnout occurring as the final stage - and in the long term resulting in a complete inability to take action.

**2.3 The physiology of the stress response**

Each individual reacts differently to stressful stimuli as a result of the intensity of the stress factors and their ability to cope with said factors. Stress occurs whenever there is an imbalance between perceived demands and available resources. The physiological response to stress is an adaptive response enabling increased resource mobilisation. Pathologies in this sense refer to the disturbance of the balance between an individual's capacity for adaptation and the demands of their surrounding environment.

It should be noted that there are two types of stress - acute stress and chronic stress - which lead to the development of pathologies.

**2.3.1. Acute stress:**

This refers to the adaptive component.

There are multiple biological mediators involved in this respect (37).

An isolated event perceived to be a threat or an uncertain or unpredictable situation leads to acute stress and the search for striking a new balance. The three stages involved here are the alarm stage followed by adaptation and then the resistance stage.

**The alarm stage**

In this stage the brain is submitted to a stressful, disruptive element which stimulates the hypothalamus and which is responsible for the double activation of hormones and neurotransmitters associated with a stressful feeling: catecholamines.

- **In the peripheral nervous system:**

This action occurs via the sympathetic autonomous nervous system (ANS) with adrenal medulla cells synthesising adrenalin and noradrenalin, the hormonal messengers responsible for cardiovascular stimulant effects as part of the organism's reaction to stress.

These peripheral catecholamines then activate the solitary tract via the vagus nerve and the locus coeruleus of the rostral medial region of the pons.

Neurotransmitters (noradrenalin, serotonin, dopamine) and peptides (CRH, AVP) act in the minutes following the appearance of a stressful event and end just as quickly to heighten alertness, an ability to size up the situation and decision-making skills.

- **Central nervous system:**

The locus coeruleus activates the hypothalamus by stimulating the limbic and prefrontal regions of the brain which are rich in adrenergic alpha and beta receptors.

The locus coeruleus, by interacting with the amygdala, plays a key role in orchestrating behavioural and biological responses to stress. The activation of the amygdala is modulated by its neuronal connections: on the one hand there is the prefrontal cortex (the main region controlling the cognitive assessment of information received) whilst on the other hand there is the hippocampus (a key structure for recalling similar experiences from the past) (44).

More particularly the stimulation of the locus coeruleus activates the prefrontal cortex via multiple non-adrenergic projections. The stimulation of the amygdala results in the quick release of neurotransmitters such as dopamine, acetylcholine, serotonin and adrenalin as well as peptides such as corticotropin (or CRH: cortisol releasing hormone) (36).


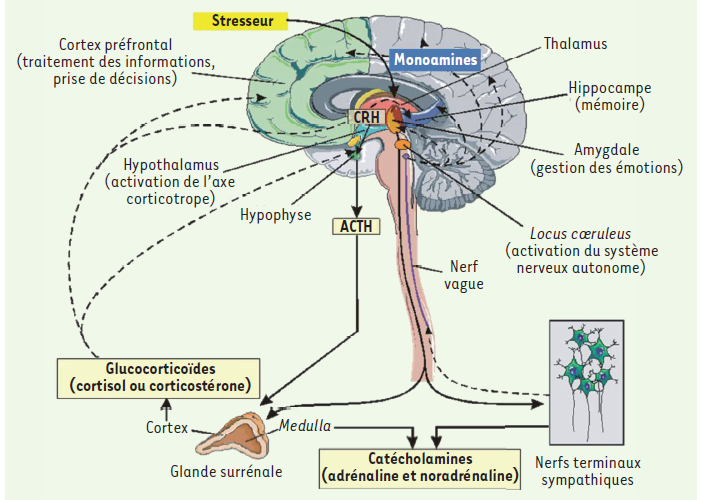


**Figure 1: Biological mediators involved in the stress response.**

**Adaptation stage**:

If the stimulus lasts too long energy from the whole body will be required, with the endocrine system duly taking over here.

The HPA axis is activated by CRH synthesis in the hypothalamus which then leads to the production of adrenocorticotropic hormone (ACTH) in the blood. ACTH then stimulates the synthesis and release of glucocorticoid hormones - namely cortisol - from the adrenal gland (cortex) (50).

Glucocorticoid action occurs later, within the next half hour, with effects lasting for several hours for an extended adaptive response aiding memory consolidation of stressor-related information (46).

The physiological response caused by catecholamines is as follows:

- increased heartbeat

- increased respiratory rate

- increased oxygen fixation and transport

- increased glycaemia

- increased amounts of fatty acids in the blood.

The effects of glucocorticoids are as follows:

- reduced growth

- weakening of the immune system

- reduced reproduction system function

- increased glycaemia

- increased amounts of fatty acids in the blood.

These physiological effects allow the body to save tremendous amounts of energy (reduced growth, reduced immune and reproduction system function) and increase the availability of energy substrates.

Adaptive redistribution is observed to cope with stressful situations.

**The resistance stage**

The above-mentioned biological changes should be limited over time so as not to affect the organism.

**2.4. The adrenal glands**

These glands are located anatomically above the kidneys and consist of a cortex and medulla.

**2.4.1. The adrenal medulla**

The adrenal medulla is populated by chromaffin cells, the function of which is storing and secreting catecholamines. Each cell stores and manufactures a single type of hormone, with 80% dedicated to adrenalin, 16% to noradrenalin and 4% to dopamine. These cells are controlled by the sympathetic nervous system.

Catecholamines have a very short half-life once released - around one minute or so and are regulated by the liver, which is capable of secreting an adrenalin-inhibiting substance and/or capturing noradrenalin.

**2.4.2. The adrenal cortex**

These cells are where glucocorticoids are manufactured and serve as a precursor to cholesterol; as such these hormones can be considered under the steroid category. They circulate in the blood by means of transportins. The underlying mechanism behind cortisol is detailed further below.

These various hormones are mainly regulated by the liver.

Cortisol contains a feedback loop as part of its control mechanism which plays a vital role in the response to stress.


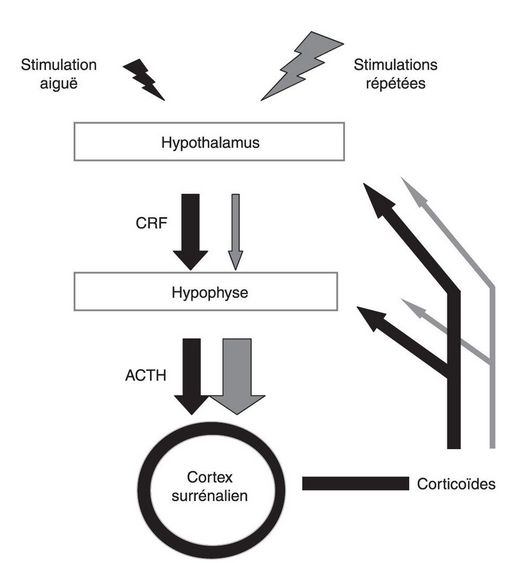


Figure 2: the cortisol feedback mechanism.

It has been observed that two categories of hormones are involved in stress response. As such the HPA axis will now be considered in great detail, as the assays used as part of this study are based around salivary cortisol levels.

**2.4.3. The HPA axis and cortisol**

1. **HPA axis physiology**

The stimulation of the HPA axis (HPA standing for hypothalamic-pituitary-adrenal) enables the hypothalamus to synthesis a hormone - CRH (cortisol releasing hormone) - giving the order to the pituitary gland to secrete ACTH (adrenocorticotropin hormone), amongst others. The cortisol and glucocorticoid secreted by the adrenal glands is controlled by ACTH. Cortisol regulates its own secretion using feedback mechanisms, which play a vital role in the organism adapting to the stress response (48).

Cortisol synthesis occurs in the zona fasciculata of the adrenal cortex and is made using endogenous and exogenous cholesterol (49). Cholesterol is firstly converted to pregnenolone and then progesterone. Progesterone undergoes two consecutive hydroxylation processes to then be converted into corticosterone or three consecutive hydroxylation processes to yield cortisone or cortisol (51). The aforementioned biosynthesis of adrenal cortex hormones occasionally involves both mitochondrial (desmolase and 11-beta-hydroxylase) as well endoplasmic reticulum enzymes (3-beta-dehydrogenase, 17- and 21-hydroxylase).


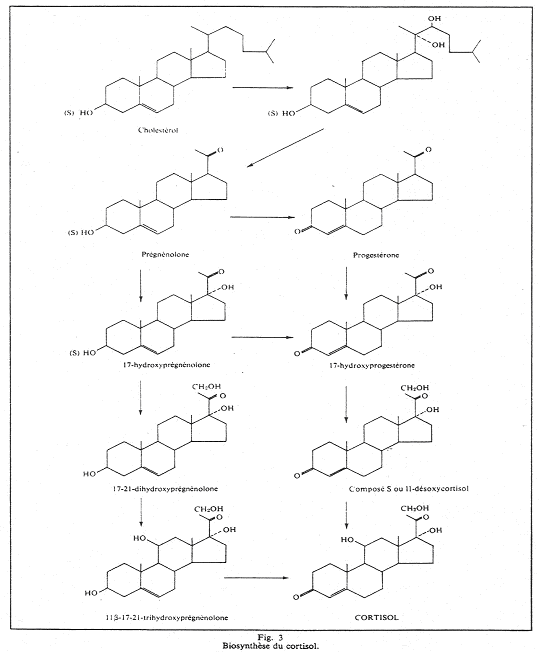


90% of plasma cortisol is bound to CBG (corticosteroid binding globulin), although the binding is reversible and the active part of cortisol is the part which has not been bound.

Both parts which are free and bound in the plasma can be measured, however for saliva only the free parts can be measured (50, 52, 53, 54, 55, 56, 57, 58).

This correlation provides a significant advantage for studies carried out in the field (59).

1. **Applications and use of cortisol**

The main function of corticoids is regulating stress-induced responses. To date multiple studies have shown that cortisol - and more specifically salivary cortisol - is a highly reliable biomarker in terms of measuring physiological and mental stress levels (54, 57, 58, 59, 60).

The spontaneous variation for cortisol is around 15 minutes (61).

For the optimum usage of salivary cortisol levels (62, 63), all spontaneous or induced variations should be recorded over 24 hours. Like a good number of other biological variables, cortisol levels regularly fluctuate over this period.

Cortisol levels respond to Circadian processes (61). The rhythm cortisol adheres to is as follows: plasma concentrations are very weak in the evening, followed by a gradual build-up of concentrations with the acrophase occurring in the morning and peak cortisol levels 45 minutes following the CAR (cortisol awakening response) (64, 65, 67) after which levels drop over the rest of the day (66, 68).

Other factors of change which play a part in cortisol levels are:

- whether the individual is standing (69, 78)

- sex (70, 74, 75, 84, 87, 88, 89, 90, 91)

- mealtimes (72, 80)

- consuming tobacco products (73, 76, 84)

- consuming coffee (74, 75)

- consuming alcohol (84)

- light levels (79, 77)

- taking medication (84, 92, 90)

- the menstrual cycle (70, 92, 90)

- pregnancy and nursing (84)

- age (84, 85)

- weariness/fatigue (86)

- psychiatric pathologies (82, 93)

- doing sport (71, 81, 94)

Accurate control over environmental, behavioural and endogenous factors involved in the regulation of cortisol secretion would therefore appear to be an inevitable methodological pre-requisite in terms of studying salivary cortisol. Measuring cortisol levels at one given point in time over the course of the day would not constitute an absolute reference value as levels could double or triple at another given point in time, being influenced by Circadian or behavioural components.

**2.4.4. From chronic stress to pathologies**

**The physiopathology behind the generation of chronic stress**

Chronic stress is associated with increased cortisol production. The first person to put forward this hypothesis was R.M. Sapolsky (95), who studied the flow of glucocorticoids to explain changes in brain function as a result of prolonged exposure to glucocorticoids.

The prolonged secretion of glucocorticoid hormones damages hippocampus neurons in particular and can lead to the tonic hippocampus’s inhibition effect being lifted with respect to the activation of the HPA axis.


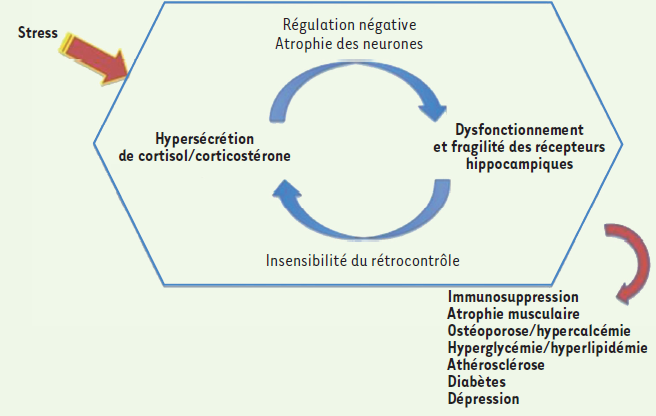


**Figure 3: The mechanism behind the deleterious effects of chronic stress.**

This leads to morphological changes in the brain in the prefrontal cortex with altered attention span, working memory and behavioural flexibility. Changes to the hippocampus are associated with spatial memory disturbances and reduced learning capacity whereas in the amygdala there is an increased memory of fear, anxiety and aggression (96, 97).

**B. HPA function and pathologies**

The HPA axis appears to be a major system in terms of the development of adaptive behavioural and physiological processes. However the toxic effects the prolonged release of glucocorticoids has on the brain (98, 99) as well as the unpredictable effects of prolonged depression on immune defences leads to the assumption that an abnormally high level of glucocorticoid secretion may make their function non-adaptive.

At this point all of the various pathologies can be linked to a disturbance of the HPA axis and can therefore be presumably linked to stress.

1. **At the psychiatric level**

Major depression may be the result of a counter-regulation anomaly in general stress responses arising from CRH and/or central catecholamine hypersecretion (100, 101).

Major symptoms of melancholic depression indicate this pathology is not associated with a state of inactivation as the name would lead some to believe, but rather pathological hypervigilance (102, 103, 104).

Stress would likewise increase the chances of sleeping disorders occurring (111, 112, 113).

1. **At the cardiovascular level**

Stress is a secondary cardiovascular risk factor in its own right (107).

Stress, as a result of hyperactivity in the HPA axis, is responsible for the development of metabolic syndrome (103, 109, 110). It likewise plays a role in the development of coronary calcification via the same mechanism (104, 105) and fosters the onset of acute coronary syndrome (108). Cortisol likewise fosters the onset of high blood pressure (114) via a sodium retention and volume expansion mechanism.

1. **At the digestive level**

Stress has been observed as playing a major role in chronic inflammatory bowel disease, altering motility, levels of sensitivity and the secretory function of the gastrointestinal tract (115).

For a long time now stress has been known to be the cause of "stress ulcers" during intensive care (116).

1. **At the endocrine level**

Connections have been made between stress and increased consumption of food (117) as well as stress and reduced physical activity (121).

Stress may also be a factor behind infertility (118).

It plays a readily understandable role in altered glycaemic function as well as the onset of diabetes (119). It may also affect thyroid function.

In addition to the above stress may also play a role in cellular aging (122).

1. **At the haematological level**

Stress may play a role in rheumatoid arthritis flare-ups (123). Stress may also have an impact on fibromyalgia (125,126).

1. **At the dermatological level**

Stress may play a role in causing disorders such as eczema, pruritus and hives (124).

1. **At the immunological level**

Stress, as a result of increased levels of plasma cortisol, may play a role in reduced immune defences and - directly or indirectly - impact on the formation of viral diseases or tumours (127, 128, 129).

1. **Professionally:**

In the workplace and more particularly in the healthcare sector, chronic stress may lead to professional exhaustion syndrome or burnout (130, 131, 132), which is a state of emotional, physical and mental exhaustion.

**2.5. Population studied**

The purpose of this study is to examine stress during emergency dispatch scenarios.

The population studied was CHR Metz-Thionville (Hôpital de Mercy) Centre 15 staff, including ARMs (emergency medical assistants), emergency doctors and general practitioners who met the eligibility criteria for inclusion.

**2.5.1. ARMs - their general mission**

ARMs are hospital employees who carry out a specific task in managing emergency medical assistance and continuous care requests which reach the SAMU (French Emergency Medical Assistance System) Centre 15 call dispatch centre.

These employees jointly carry out the missions they have been assigned under the legislative and regulatory framework and are under the operational supervision of dispatch and hospital doctors and general practitioners. ARMs continuously report their actions to the foregoing.
They are responsible for control room equipment and keeping all SAMU Centre 15 documentation - such as the patients identified, emergency plans, rotas, operational directories, etc. - up to date.

**2.5.2. ARMs – general skillset**

ARMs must be able to:

- Understand the organisational structure and mission behind emergency medical assistance.
- Receive all calls.
- Weed out - via brief yet specific questioning - non-medical calls, processing the relevant enquiries and prioritising them based on the degree of emergency.
- Detect life-threatening emergency situations.
- Manage communications and adapt to callers' reactions and levels of stress.
- Pass on all relevant information to dispatch doctors.
- Apply decisions per response procedure and as indicated by dispatch doctors.
- Understand the whole of operational procedures and documentation involved for daily dispatch scenarios as well as for crisis events.
- Understand what resources are available at the pre-hospital and hospital level for the French department (similar to a British county), region and the zone of defence.
- Understand the criteria used to diagnose the severity of major emergency pathologies and emergency care actions and procedures.
- Have a full command over information, communications and IT tools.
- Be capable of completing the operational and administrative documents linked to the incidents being processed as well as ensuring the operational maintenance of the dispatch centre.

**2.5.3. The role of dispatch doctors**

**The role of emergency doctors:**

- Identifying P0 and P1 (call priority) incidents
- Identifying B assessments
- Dealing with requests for inter-hospital transfers
- Dealing with requests from other SAMUs
- Dealing with SMUR requests
- Operations on public roads
- SMUR assessment

**The role of general practitioners or non-emergency care hospital doctors**

- Identifying P2 incidents
- Responding to requests for medical advice
- Dealing with calls related to continuous outpatient care

**3. Research objectives:**

**3.1. Main objective:**

Describe biological stress via cortisol levels as well as perceived stress via a visual analogue evaluation for stress in ARMs and doctors when processing a call at Centre 15.

**3.2. Secondary objectives:**

- Compare levels of both perceived and biological stress in ARMs amongst the various shifts

- Study the difficulty of the tasks required of the various ARM shifts

- Study the correlation between perceived and biological stress

- Study the correlation between the severity of the situation being dealt with over the phone and stress

**4. Research design**

This was a controlled physiological study carried out in healthy human beings, with each subject acting as their own witness.

Four experimental conditions were used for ARMs whilst two experimental conditions were used for emergency care doctors and private practitioners.

**4.1. Clear statement of the** **main evaluation criteria AND, where applicable, secondary evaluation criteria**

- Main evaluation criteria

**Objective measurement of stress: salivary cortisol assay under various experimental conditions.**

4 experimental conditions should be applied to ARMs: "call, action, evaluation, witness"

2 experimental conditions should be applied to doctors: "call, witness"

- Secondary evaluation criteria

**Subjective measurement of stress:**

**- ASA: acute stress assessment**

Acute stress assessment via the ASA form should be carried out concomitantly to each series of salivary cortisol samples being taken. This assessment form requires each subject to rate their level of stress on a scale of 0 to 10. The ASA has been validated for studies on stress (133, 134, 135, 136, 137).

**- PNOSI questionnaire: Positive and negative occupational stress inventory: chronic stress assessment**

The PNOSI chronic stress questionnaire should be completed only once by each subject. It will be distributed at 10:00 in the morning in Centre 15 (at the start time of the study) to the whole of participants that day and prior to taking any salivary cortisol samples (138, 139, 140).

**- NEO PI-R personality test: (the revised NEO personality inventory) (141): evaluating subjects' individual personality components.**

The NEO PI-R personality test should be completed only once by each subject. It will be distributed during working hours, with participants being given time off duties to complete the inventory.

This questionnaire contains 180 questions for participants to answer and is supervised by the Mercy CHR occupational psychologist. Once completed questionnaires will then be analysed by said occupational psychologist.

## 4.2. Description of the research methodology including a schematic presentation covering scheduled visits and testing.

- **Pre-inclusion visit**

The pre-inclusion visit should take place between *30 days* and no later than *15 days* before the inclusion visit.

Information on study progress and objectives will be presented by the investigator.

All PARMs (medical emergency dispatch centre call assistants) and doctors should attend this meeting.

- **Inclusion visit**

A questionnaire containing study eligibility criteria will be distributed to every ARM and unit doctor in addition to an information note on the study itself and an informed consent form.

After this visit only eligible individuals who have signed and submitted an informed consent form may participate in the study.

- Monitoring visits

**During these visits salivary cortisol assays will be performed and the relevant questionnaires (ASA and PNOSI) will be completed.**

**4.2.1. CHR METZ-THIONVILLE Centre 15 organisational structural and experimental conditions being assessed**

ARMs work 12-hours shifts every night with rotation happening every two hours, followed by three days off for a total of 36 hours a week.

Emergency doctors also work 12 hour shifts for a total of 48 hours a week, however they rotate between the medicine department, dispatch and SMUR/resuscitation.

Private practitioners more often than not work shifts of 12 hours.

The operational structure of Metz Centre 15 is as follows: medical dispatch assistants (ARMs) are assigned to one of three areas:

- **Call ARMs** provide the first line of support, answering all incoming calls. They assess the severity of the situation at hand and, based on this information, transfer the call to either an emergency doctor or a general practitioner. This position is the most stressful of the three.

- **Action ARMs** provide various means of intervention. This area organises patient transport and is responsible for ringing up ambulances, the fire brigade or SMUR teams based on what is required for the matter at hand. They only act once they have received due instruction from either a general practitioner or emergency doctor.

- **Assessment ARMs** are in charge of collecting reports from the various dispatch assignments.

The way the doctors work however is quite different. Call ARMS send independent practitioners (who are more often than not general practitioners) less serious calls which still require medical advice or just an ambulance. Emergency doctors manage the more severe situations which require more in the way of support; they are the only ones who can authorise an SMUR dispatch, as an example.

This study will be conducted by including the same number of experienced ARMs (i.e. those with over five years of experience) as younger ARMs (i.e. those with less than five years' experience). The same dispatch assistant will also be studied whilst working in each of the three areas.

During this study one call will firstly be studied, which will act as the control or reference. A series of salivary cortisol samples will then be taken from the call ARM at that time. The first sample will be taken when the ARM hangs up the phone, which is the T0 sample. Additional samples will then be taken every 15 minutes over 90 minutes, with the final sample being taken at two hours from T0. Once the call is transferred to the doctor - either the emergency doctor or SMUR doctor - samples will be taken from the doctor in question, with T0 being the moment when the doctor ends the call and takes the swab. The same call will be studied in exactly the same way for action ARMs and assessment ARMs.

All calls will be studied in this way.

**4.2.2. Practical organisation of the study**

.

***SALIVARY CORTISOL ASSAYS***

***Materials***

The assay will be carried out using Biomnis or Sarstedt Salivette swabs in combination with ASA subjective stress measurements.

Swabs will be provided by either Biomnis or Sarstedt and consist of a cotton tip which must be thoroughly soaked with saliva, which is achieved by biting down on for two or three minutes (see the annexes for more information). Samples will then be rendered anonymous and coded for confidentiality purposes. The swabs will then be stored in a Mercy Hospital laboratory freezer at -4 degrees Celsius for local analysis later. Samples may be stored at a temperature of -4 degrees Celsius for one year.

***Experimental conditions***

Salivary cortisol will be collected for each ARM over three working days in three different positions: calls, resource deployment or action, and assessment.

Participants will be notified of proper sampling conditions for the salivary cortisol assay. Once enrolled on the study participants shall undertake not to drink or eat anything for half an hour prior to the sample being collected as well as during the two hours when samples are taken. Subjects must also agree not to smoke thirty minutes prior to samples being taken as well as during the sample collection period. Regarding other variation factors which affect salivary cortisol levels, light levels will not vary over the working day and subjects will be working in a seated position, so standing will not be accounted for.

Calls assays will be taken for will be for the same time period to prevent any bias arising in terms of inter- and intra-individual circadian variations in salivary cortisol levels.

The first incoming call for the ARM shall be used as the reference, however accidental or mistaken calls will be excluded from the study. During the call, a salivary control assay will be taken for the call ARM, with the first assay being marked T0. Additional assays will be conducted for call ARMs every 15 minutes after T0 for 1 hour and 30 minutes, followed by a final assay at two hours after the call, which translates to eight assays which will be used to generate the acute stress curve. Cortisol assays will all be carried out at the same time to prevent nycthemeral variations. Each sample will be taken as soon as the subject ends the call and hangs up the phone.

The same salivary cortisol assay following the same principle will then be carried out on the doctor the call was transferred to. The same assay will also be carried out on the action ARM and the assessment ARM. The next day the salivary cortisol assay will be carried out on the same ARMs but as assigned to different areas.

Three medical dispatch assistants should therefore have salivary cortisol assays administered every day as should a doctor - either an emergency doctor or independent practitioner - based on the severity of the call.

Each additional call will be noted when taking salivary cortisol measurements. Each event over the two hour sampling period will likewise be recorded to prevent any bias arising and for a better understanding and analysis of the curves generated.

Salivary cortisol assays should likewise be administered on days off (the day after a working night shall not be deemed to be a day off) to obtain reference values and limit intra-individual variation.

This assay will be carried out per a different methodology as the purpose here is not studying acute events but rather for the generation of a reference curve. Eight samples shall be taken at regular intervals over the course of the day. The first sample will be taken when the subject wakes up and shall be used as T0, followed by samples taken every two hours. Samples should be stored at room temperature by participants and then taken to the CHR the next day. Each participant will be provided with an explanatory note.

Saliva samples will be made anonymous. Each subject shall be assigned a number for each time an individual sample was taken.

Samples shall be stored at -4 degrees Celsius in a dedicated freezer, which has been kindly provided by the Mercy Hospital laboratory. Samples will then be analysed in the Mercy CHR laboratory at a later date.

**4.2.3. Completing the stress evaluation questionnaires**

* PNOSI (chronic stress): this should be completed once by each subject at Centre 15 at 10:00 in the morning (when the study officially starts) and prior to having the first salivary cortisol samples taken.

* ASA (acute stress): this questionnaire shall be completed concomitantly with each cortisol assay. Each subject shall rate their level of stress on a scale of 1 to 10.

* NEO PI-R (personality test): this should be completed once by each subject at Centre 15 and under the supervision of the Mercy CHR occupational psychologist.

**4.2.4 Other data collected**

Intervening events (such as other calls, specific contexts, etc.) shall be recorded during periods where salivary cortisol assays are being taken (i.e. over 2 hours per each subject).

Furthermore during each call, the gravity of the situation at hand will be assessed using the following classification to look for a correlation between physiological stress levels and the seriousness of the call.

For ARMs:

Three levels of priority shall be applied when transferring the call to a dispatch doctor:

P0: procedural response of an SMUR team being sent followed by priority dispatch

P1: priority dispatch

P2: dispatch that can be put on hold without risk to the patient until other dispatches which are currently out can arrive

For dispatch doctors:

Dispatch doctors make their decisions based on four levels of emergency:

R1: a patent or latent life-threatening emergency requiring a resuscitation team (SMUR) being dispatched.

R2: a real emergency although not life-threatening which would require the attention of a nearby doctor, ambulance or the fire brigade within a period of time contractually agreed to between the dispatcher, the acting party and the caller.

R3: access to continuous care required although the time it takes to do so presents no immediate risk; a medical prescription for later may be suggested.

R4: medical advice or drug prescription can be provided over the phone.

For assessment ARMs:

Three levels of priority are applied by ARMs when forwarding assessments to dispatch doctors:

B0: assessment with signs of distress which were not apparent during the call. This call should be answered as a priority by the emergency dispatch doctor.

B1: assessment providing additional information which was not present during the initial dispatch but absent of any signs of distress or seriousness.

B2: an assessment in line with the data collected during the call to dispatch.

## 4.3. Description of measures taken to reduce and prevent biases from arising, more specifically for the purposes of ensuring random draws and blinding methods

This study will be conduct openly and does not require a random draw.

Each subject will be their own witness. Subjects will be assessed under identical experimental conditions which are specific to their position (ARMs and medical personnel).

See point 4.2. for more information in this regard.

## 4.4. Expected length of individuals' participation

Inclusion period length: 1 month

Length of each individual's participation: four instances of two hours for ARMs and two instances of two hours for doctors.

ARMs: four instances of 8 samples each / Doctors: Two instances of 8 samples each

Total length of the study: 6 months

**4.5. Identification of all data to be recorded directly in case report forms which shall then deemed to be source data.**

Not applicable.

**5. Selection and exclusion criteria for research participaNTS**

**5.1. Inclusion criteria**

- Centre 15 medical and paramedical employees (medical dispatch assistants, emergency doctors, independent practitioners):
- Who have received information about the study
- Who have signed a consent form to participate in the study
- Who are on a compulsory membership with a social security scheme

**5.2. Exclusion criteria**

- Chronic alcoholics
- Women who are on combination oestrogen plus progestin treatment
- Pregnant women
- Highly athletic individuals
- Individuals on steroid-based medical treatment
- Individuals being monitored for psychiatric disorders (mainly depression)
- Individuals presenting with fever on the day samples are taken
- Volunteer participants who have endocrine diseases

**5.3. Early withdrawal from the study or exclusion (END OF MONITORING)**

**Criteria and procedures for ending monitoring**

- *Definitively*

Upon request of the subject

- *Temporarily*

Not applicable

**5.4. Recruitment procedures**

Metz-Thionville CHR Centre 15 medical and paramedical employees (emergency call staff, emergency doctors, independent practitioners) shall be notified this study is being carried out. Each individual employee will then be asked to participate in the study as long as they are eligible to do so per the criteria above.

*If over 20 individuals express their desire to participate in the study a random draw will be held to decide who the participants will be.*

**6. DESCRIPTION OF EXPOSURE**

*See point 4.2. above.*

**7. Safety evaluation:**

**7.1. Description of safety evaluation parameters**

The sole constraints placed on subjects participating in this study are with respect to the salivary cortisol assays which will be taken using a swab, i.e. a small cotton bud subjects will have to chew on to thoroughly soak through with saliva (see Annex 1).

Within the framework of this study the only foreseeable risks are those connected to the use of the swabs, namely an intolerance to the strain of cotton used or misuse of the swab.

**7.2. Planned methods and timetable to be used in terms of measuring, collecting and analysing safety evaluation parameters**

Subjects participating in the study shall be interviewed regarding the risks listed above in section 7.1.

**7.3. Procedures in place for the recording and reporting of adverse events**

**7.3.1. Definitions**

An **adverse event** refers to a harmful event affecting a person undergoing biomedical research regardless of whether said event is related to research, the product being researched or any other factor.

An **adverse effect** refers to a harmful, undesirable reaction related to the experimental drug being taken which occurs after taking said drug at the dosage normally recommended for humans.

A **serious adverse event or effect (SAE)** is an event or effect which may contribute to the patient's death, endangerment of their life, the hospitalisation of the patient or extended hospitalisation, a significant or lasting disability or an event of effect which leads to a birth defect.

**The list of expected adverse effects** related to this study has been drawn up using the reference document.

An SAE is deemed to be unexpected (unexpected SAE) whenever it is not included in this list.

**A new discovery** may be: the unexpected frequency of an unexpected SAE, severe adverse events related to study procedures, insufficient effectiveness in treating life-threatening diseases and/or non-clinical data.

**7.3.2. Reference document to be used to define the unexpected nature of an SAE**

*Not applicable within the framework of this study*

**7.3.3. List of expected adverse events**

*Not applicable within the framework of this study*

**7.3.4. Communication of SAEs and new discoveries**

As soon as an investigator is made aware of an SAE or new discovery, he/she should let the promoter of the study know by faxing him/her the SAE declaration form.

- If the SAE is unexpected or if notification is for a new discovery, the promoter should contact the investigator for the purposes of preparing an initial report which will be sent to the ANSM, the IRB and the principal investigator within 7 days in the event of a death. For instances of endangerment of the patient's life this timeframe shall be extended to 15 days.

When the event has not been resolved by the date the fax is sent, the investigator shall be under obligation to send an additional report for the purposes of documenting the patient's progress or provide an update on missing data.

- For expected serious adverse events all files shall be compiled by the promoter when drafting annual safety reports.

**7.3.5. Notification of non-serious adverse events**

These should be briefly described by the investigator in the summary sheet included for this purpose in the case report form.

**7.4. Monitoring procedures and timescales following the appearance of an adverse event**

Whenever a serious adverse event persists, including after the end of the study, the investigator shall continue monitoring the patient until the event is deemed to have been resolved and shall then provide the promoter with all data collected during monitoring.

**7.5. Specific research committees (may vary based on the protocol)**

**Steering committee**

This committee shall be made up of the clinician initiators of the project, the biostatistician responsible for the project and promoter representatives.

This committee shall define the general organisation and procedural methods for research and co-ordinate all information.

It shall be responsible for initially determining the methodology to be used and shall decide, over the course of research, what steps to take in the event of unforeseen circumstances and oversee research progress, particularly in terms of tolerance and adverse events.

**7.6. Safety reports**

- Annual safety reports: the promoter shall draft annual safety reports and send these to the ANSM, the IRB and the principal investigator. The principal investigator shall provide the promoter with all data required to prepare this report.
- Final report: this report shall be prepared by the promoter and principal investigator within one year of the end of the study. All investigators shall be notified of study results. A summary shall be sent to the ANSM by the promoter.

**8. Statistics**

**8.1. Description of anticipated statistical methods to be used, including a timetable for any anticipated interim analyses**

**Description of sampling**: qualitative variables shall be described as a percentage, using the absolute frequency and a 95% confidence interval, with quantitative variables being described as an average and standard deviation or as medians over a range and with interquartile intervals.

**Description of biological stress**: this shall be described by calculating the difference between cortisol peak(s) observed and cortisol levels measured on the day off (as an average and standard deviation or as medians over a range and with interquartile intervals).

**Description of perceived stress**: the ASA score shall be described as a median and standard deviation.

**For exploratory purposes**: a comparison of the delta peak/cortisol base rate from the three ARM posts using a non-parametric Kruskal Wallis test, with the same being carried out based on ARM seniority, the seriousness of the call and the doctor's status (emergency doctor or independent practitioner); a comparison of the delta peak/cortisol base rate per chronic stress levels (PNOSI questionnaire) and personality (NEO PI-R questionnaire).

The ASA score (perceived stress) shall be compared to the delta peak/cortisol base rate (biological stress) using the Spearmann correlation coefficient.

**8.2. Planned number of individuals to be included in the study with the statistical grounds for the number given**

This is a study where the main objective is descriptive; additionally it may serve a purpose as a feasibility study with respect to the comparative (secondary) objectives. 20 ARMs and 20 doctors (emergency doctors or independent practitioners) are expected to be included.

**8.3. Planned degree of statistical significance**

Again, this is a descriptive study. Statistical tests shall be carried out for exploratory purposes with alpha risk set at 5%.

- 1. **Statistical criteria for ending the research**

Not applicable

**8.5. Method used to account for missing, unused or non-valid data**

Missing data will not be extrapolated. Subjects for which half or more of data are missing shall be excluded from analysis.

**9. Right of access to data and source materials**

All data and information on voluntary participants shall be held in the strictest of confidentiality. Individuals who are entitled to a right of access in accordance with current legal and regulatory provisions, namely articles L.1121-3 and R.5121-13 of the French Public Health Code (i.e. the investigators, individuals responsible for quality control, monitors, clinical research assistants, auditors and anyone requested to help in the conduct of the assays) shall take all necessary precautions to protect the confidentiality of study-related information and people participating therein, particularly with respect to the latter's identity and results. Data collected by these individuals when carrying out quality control procedures or audits shall therefore at all times be anonymous.

Subjects shall be identified using a chronologically assigned number based on the order of their inclusion in the study. Data shall be collected such that it would be impossible to indirectly identify participants, only noting: the participant's sex, their length of employment with the service (greater than or less than five years) and age in ranges of 10 years.

**10. Quality control and assurance**

**10.1. Monitoring**

The CRAs (clinical research assistants) representing the promoter shall make visits to the centre under investigation at the frequency described in the subject monitoring plan in the protocol:

- An initial visit to the Centre: this shall occur prior to inclusion to establish the protocol to be used and to meet the various individuals working on the biomedical research side of the study.

- During the following visits case report forms shall be reviewed by the CRAs as research steadily progresses. The whole of investigators enrolling subjects in the study or who are carrying out monitoring shall undertake to receive visits from the CRAs at regular intervals.

Said visits shall be carried out in accordance with Good Clinical Practice, with the following elements being reviewed during this time:

- Fulfilment of the protocol and procedures defined for research purposes,
- Verification of subjects' informed consent forms,
- The examination of source documents and comparison of data reported in the case report forms, with respect to the exactitude thereof, missing data and data consistency.

- Closing visit: this is the last visit for regulatory compliance - where applicable - and also covers preparation for the filing of study documents.

To this end the investigator shall undertake to provide CRAs with the following during each of their monitoring visits:

⮚ The source files for subjects (questionnaires, salivary assays, etc.)

⮚ Data collection files (case report forms)

⮚ Consent forms for subjects included in the study

**10.2 Transfer of data to the case report forms**

All information required under the protocol should be provided in the case report forms with an explanation given by the investigator for each instance of missing data.

Data should be recorded in case report forms as they are received with respect too clinical or paraclinical data. Data should be wholly and legibly copied over in black ink (for the purposes of easy duplication and digitalisation).

Any erroneous data detected in the case report forms should be cleanly struck through, initialled and dated by the investigational team member making the correction, with the new, correct data being copied over onto the form.

Subject anonymity shall be ensured by using a code number and the initials of the individual being researched on all documents required for research or by deleting - using the appropriate means - of all of the personal data on the copies of source documents to be used as research documentation.

Computerised data held on file should be reported to the CNIL (French National Commission for Information Technology and Civil Liberties) per the procedure to be used for this instance (see section 11.4 below).

**11. Ethical and regulatory considerations**

The promoter and investigators shall undertake to ensure research is carried out in accordance with French Law no. 2004-806 of 9 August 2004 as well as in accordance with Good Clinical Practice (I.C.H. version 4 of 1 May 1996 and the Decision of 24 November 2006) and the Declaration of Helsinki (Ethical principles for medical research involving human subjects, Seoul 2008).

Research shall additionally be conducted in accordance with this protocol. The investigators shall undertake to comply with every single point included in the protocol, particularly with respect to obtaining subject consent and the notification and monitoring of severe adverse events.

**11.1. Request for ANSM approval**

Prior to undertaking research the promoter should submit a request for approval to the relevant authority, i.e. the ANSM (French National Medicinal Drug and Healthcare Product Safety Agency). This relevant authority, as defined in article L. 1123-12, shall make a decision with respect to the safety of the individuals undergoing biomedical research, namely taking into account the safety and quality of the products used over the course of research in accordance with - where applicable - current applicable benchmarks, conditions of use and personal safety for the acts being carried out and the methods used as well as the planned individual monitoring procedures.

**11.2. Request for an opinion from the Institutional Review Board (IRB)**

Per article L. 1123-6 of the French Public Health Code, the research protocol should be submitted to the Institutional Review Board (Comité de Protection des Personnes) by the promoter. The promoter should notify the relevant authority of the opinion issued by this committee prior to undertaking research.

**11.3. Changes**

Changes made to the study protocol should be categorised as either substantial or non-substantial.

A substantial change is deemed to be a change which may - one way or another - affect the guarantees made to individuals undergoing biomedical research (such as changes to the inclusion criteria, an extension of the inclusion window, the participation of other centres, etc.).

Once research has started any substantial changes made by the promoter shall require - prior to implementation - the issuance of a favourable opinion by the institutional review board as well as approval by the relevant authority. In this case and where necessary the board shall ensure a new consent form for individuals participating in research has been drafted and collected.

Any substantial change made shall be subject to issuance of a request for approval with the ANSM and/or a request for an opinion from the IRB by the promoter.

**11.4. Declaration for the CNIL**

This study falls under the "Reference methodology" (MR-001) framework in application of the provisions of article 54, section 5 of the amended French Law of 6 January 1978 on data processing, files and freedoms. Amendments were approved in a decision dated 5 January 2006. The Metz-Thionville CHR has signed an undertaking of compliance with said "Reference methodology."

Only data required for research shall be collected. Subjects are nevertheless entitled to oppose data concerning themselves which may be subject to automatic processing. Subjects shall at all times be entitled to access to their own data and shall likewise be entitled to request the rectification of any inaccurate data. They shall be entitled to exercise their rights at any point in time against the principal investigator, Dr Michel Aussedat.

Subjects' rights may be exercised directly or via the doctor of their choice with respect to all information of a medical nature.

**11.5. Information note and informed consent**

Prior to receiving the subject's consent the investigator shall undertake to provide the former with clear and full information - inasmuch as possible - for the study in question; the investigator should likewise provide the subject with the information note.

The consent form should be signed in duplicate by the subject and investigating doctor:

⮚ With one copy kept by the individual participating in the research.

⮚ The other copy shall be kept and filed away by the investigator.

The procedures for providing information and taking the subject's informed consent within the framework of this study is as described above in section 4.2.

**11.6. Final report**

The final report from research shall be drafted by the principal investigator alongside the biostatistician assigned to the study. This report shall be submitted to each of the investigators for their comments and opinion. Once a consensus has been reached the final version should be signed by each of the investigators and sent to the promoter as soon as possible following the research phase effectively ending. A written report which fulfils the requirements set out in the relevant authority's reference plan should be sent to said authority as well as the IRB within one year of research ending; "research ending" shall be understood to be the date of the last monitoring visit of the last subject included in the study. This period shall be reduced to 90 days in the event of the study ending early.

**12. DATA PROCESSING AND DOCUMENT AND DATA STORAGE AS RELATES TO RESEARCH**

Documents for research studies falling under the legal category of biomedical research should be kept and stored by all parties for a period of 15 years once research has ended.

*(See GPC, chapter 8: essential documents)*

This indexed file should include:

- Copies of ANSM approval letters and the required opinion issued by the IRB
- All versions of the protocol (as identified by the version no. and date)
- Mail correspondence with the promoter
- Consent forms signed by the subjects in a sealed envelope with the inclusion list/register for comparison's sake
- Completed and validated case report forms for each subject included in the study
- All annexes which are specific to the study
- The final report involving statistical analysis and quality control for the study (submitted in duplicate to the promoter)
- Any audit certificates as were issued over the course of research

The database used for statistical analysis should also be kept and stored by the individual in charge of analysis (in hard or soft copy).

**13. Funding and insurance**

**13.1. Insurance**

The promoter has contracted insurance via the SHAM (French Hospital Mutual Insurance Company) covering their own civil liability as well as the civil liability of any stakeholder involved in the study, regardless of the nature of the ties between stakeholders and the promoter for the whole of the term of the study.

### 13. 2 Funding

This study has been submitted to various calls for projects for the purposes of covering the specific expenses arising as a result of its implementation.

**14. Regulations governing publication**

Metz-Thionville CHR is the owner of the data produced as part of this study, which may not be used or provided to a third party without prior consent.

Metz-Thionville CHR should be mentioned as the promoter of this biomedical study as well as the provider of financial support for said study, where applicable.

Publications should include *Sarah Bedini, Laurence Weibel and Michel Aussedat based on their involvement in the drafting of these articles* in the list of authors.

**15. List of annexes**

*Annex 1: instructions for use for the Salivette*

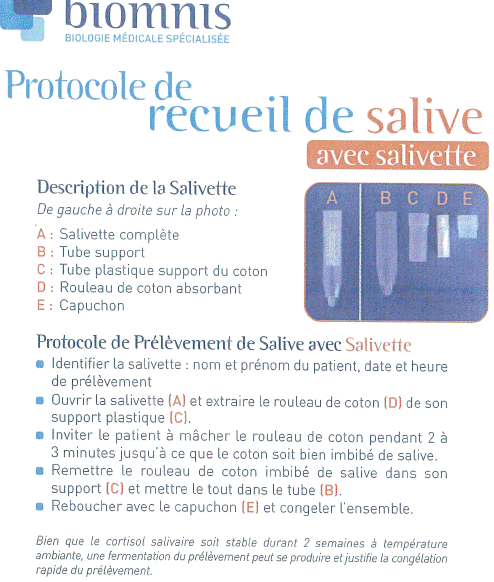


Annex 2: ASA scale


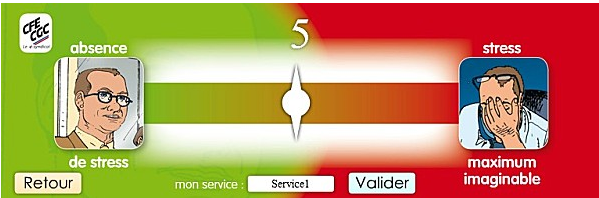


Annex 3: PNOSI questionnaire


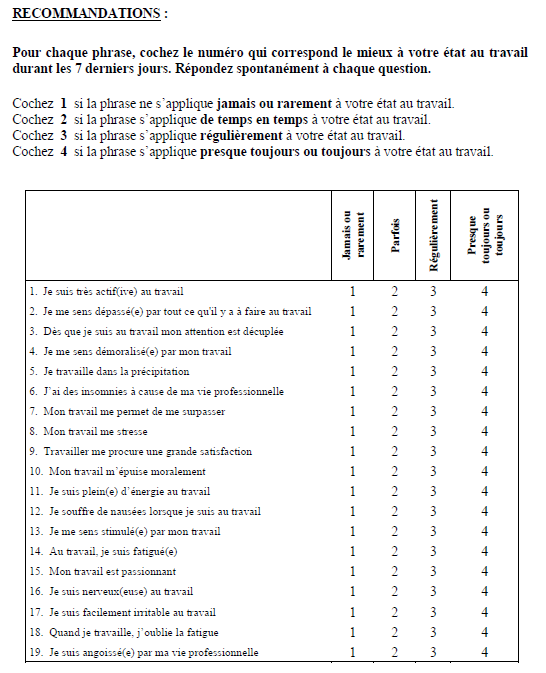

Supplement: S2 Protocol — (DOCX) [file pone.0177094.s003.docx]
